# Polymer-like tetramer acceptor enables stable and 19.75% efficiency binary organic solar cells

**DOI:** 10.1038/s41467-025-57118-9

**Published:** 2025-02-20

**Authors:** Jianxiao Wang, Cheng Sun, Yonghai Li, Fuzhen Bi, Huanxiang Jiang, Chunming Yang, Xichang Bao, Junhao Chu

**Affiliations:** 1 Key Laboratory of Photoelectric Conversion and Utilization of Solar Energy, Qingdao Institute of Bioenergy and Bioprocess Technology, Chinese Academy of Sciences, Qingdao, China; 2 Laboratory of Solar Energy, Shandong Energy Institute, Qingdao, China; 3 Qingdao New Energy Shandong Laboratory, Qingdao, China; 4 College of Textiles and Clothing, State Key Laboratory of Bio-fibers and Eco-textiles, Qingdao University, Qingdao, China; 5 Shanghai Synchrotron Radiation Facility Shanghai Advanced Research Institute, Chinese Academy of Sciences, Shanghai, China

**Keywords:** Solar cells, Polymers, Solar energy and photovoltaic technology

## Abstract

Limited by large batch differences and inferior polymerization degree of current polymer acceptors, the potential high efficiency and stability advantages of all-polymer solar cells (all-PSCs) cannot be fully utilized. Alternatively, largely π-extended and structurally definite oligomer acceptors are effective strategies to realize the overall performance of polymer acceptors. Herein, we report a linear tetramer acceptor namely 4Y-BO with identical molecular skeleton and comparable molecular-weight relative to the control polymer acceptor PY-BO. The structurally definite tetramer shows refined film-forming kinetics and improved molecular ordering, offering uniform crystallinity with polymer donor and hence well-defined fibrous heterojunction textures. Encouragingly, the PM6:4Y-BO devices achieve an efficiency up to 19.75% (certified efficiency:19.58%), largely surpassing that of the control PM6:PY-BO device (15.66%) and ranks the highest among solar cells based on oligomer and polymer acceptors. More noticeably, thermal stability, photostability and mechanical flexibility are collectively enhanced for PM6:4Y-BO devices. Our study provides an important approach for fabricating high performance and stable organic photovoltaics.

## Abstract (web summary)

The performance of all-polymer solar cells has been limited by batch-to-batch variations of polymer acceptors. Here, the authors report a linear tetramer acceptor with improved molecular ordering and uniform crystallinity with polymer donor, achieving certified device efficiency of 19.58%.

## Introduction

Organic solar cells (OSCs) emerge as promising photovoltaic technologies to utilize solar energy^1–4^. Among them, all-polymer solar cells (all-PSCs) have attracted widespread attention due to the potential of flexibility and long-term stability^5–9^. In particular, beneficial from the continuous development of polymer donors and polymerized small molecule acceptors (PSMAs), all-PSCs have achieved over 19% power conversion efficiencies (PCEs) recently^10–12^. However, one should note that most of the polymer acceptors, particularly the popular Y-analogs, exhibit a low degree of polymerization with number-average molecular weights (*M*_n_) of about 10 kDa^12–14^. This dilemma puts current polymer acceptors in a rather awkward position, this is, batch differences, inferior molecular ordering and electron mobilities compared to small molecular acceptors, and feeble intermolecular entanglement compared to highly polymer donors in all-PSCs^15^. The drawbacks of current polymer acceptors not only account for the underperforming efficiencies of all PSCs, but also the unsatisfied operation stability and mechanical flexibility^16–18^. Although there are some impressive strategies of molecular design and device engineering to enhance the performance of all-PSCs^13,19–21^, the inherent problems discussed above were not fundamentally solved. In particular, it is believed that the inferior degree of polymerization of Y-analog polymer acceptors stems from the banana-shaped molecular configurations of the monomers, which could lead to spatial passivation of the reaction sites and termination of chain propagation^22–24^. Therefore, developing approaches to realize or even outperform the merits of all PSCs should be of vital importance to the advances of efficient and stable OSCs.

Encouragingly, oligomer acceptors demonstrate the enormous potential to combine the fundamental advantages of both small molecular and polymer acceptors. (1) Oligomers possess definite conjugated molecular structures, which provide orderly molecular packing and avoid batch differences, conducing to improve photovoltaic performance and industrial compatibility^25^. (2) The high molecular-weight oligomers are usually equipped with high glass transition temperatures (*T*_g_) and thus low diffusion coefficients, which are necessary to delay morphology degradation for stable photovoltaic devices^26–28^. (3) Oligomers with largely π-extended molecular skeletons, demonstrate potential polymer-like interchain entanglement effect^21,29–31^. These provide feasibility for building high-performance, stable, and mechanically flexible devices. According to the number of polymerization units, most of the current oligomer acceptors belong to dimerized acceptors or trimerized acceptors. From the point of connecting units, the oligomer acceptors can be divided into fully π-fused oligomers, π-bridge linked oligomers, and σ-bridge linked oligomers. Besides, considering the molecular configurations, linear and star-shaped oligomers can be included^31^. Among those structures, π-bridge linked linear oligomer acceptors have drawn considerable attention owing to their molecular diversity, straightforward synthetic approaches, and strong intermolecular interactions arising from the flat and directionally extended π-backbones. Taking an example, Sun and coworkers recently reported a high-performance linear heterotrimer acceptor TQT through amalgamating two different Y-family monomers based on benzothiadiazole and quinoline units^32^. The PM6:TQT based solar cells achieved a high PCE of 18.52% with good photostability and thermal stability. Besides, the π-linkers are critical to regulating the aggregation property of oligomer acceptors. For instance, Kim and coworkers employed a planar acetylene linker for dimerization of the Y-analogs monomer and designed an oligomer DYA-I^33^. The resultant OSCs afforded an impressive PCE of up to 18.83% with an extrapolated *T*_80%_ lifetime of over 5000 h. These exciting discoveries highlight the great promise of linear oligomer acceptors in the construction of high-efficiency and stable OSCs. In spite of these achievements, one should note that on the one hand, the photovoltaic efficiencies of oligomer-based OSCs remain lagging behind, and on the one hand, the device stability, in particular mechanical flexibility as highlighted in all-PSCs still lacks deep exposition.

As we know, most of the Y-family polymer acceptors reveal limited *M*_n_s, which are equivalent to an average of four or five polymerization units and also significant batch-to-batch differences^25,34^. Further extending the π-conjugation of defined structure oligomer acceptors to tetramer or pentamer may attain more comparable physical and chemical properties with polymer counterparts especially the notable interchain entanglement and tensile property. However, in spite of the few cases related to linear tetramer or greater oligomer acceptors up to date, the resultant device efficiencies are yet very low (< 17.50%)^15,25,26^. Therefore, developing efficient tetramer oligomers remains a great challenge, which, however, could be a promising approach to fulfill or even outperform the potential advantages of all PSCs.

In this study, we report a linear tetramer oligomer acceptor, namely 4Y-BO, and a counterpart polymer acceptor, namely PY-BO, which have exactly the same molecular skeleton including monomer moiety, π-linker, and side groups. In order to disclose the great potential of the tetramer oligomer, we conduct comprehensive comparisons between 4Y-BO and PY-BO from the perspectives of molecular aggregation, photovoltaic performance, heterojunction texture, and thermal/mechanical stabilities of OSCs. Results show that compared to the excessive aggregation behavior of PY-BO, the tetramer with a defined molecular structure receives moderate pre-aggregation property, affording homogeneous crystallinity kinetics with better-organized molecular orientation. Consequently, the bulk-heterojunction blend of PM6:4Y-BO delivers a well-defined fibrous texture with uniform donor and acceptor crystallinity, offering a PCE of up to 19.75%. This impressive efficiency not only largely exceeds the PCE of counterpart PM6:PY-BO (15.66%), but also ranks the highest performance among OSCs based on oligomer acceptors so far. Besides of good photovoltaic performance, the PM6:4Y-BO devices also demonstrate notably enhanced thermal stability (*T*_80%_ lifetime of 2125 h) after continuous heated at 80 ^o^C, and photostability (*T*_92.6%_ lifetime of 150 hrs) after continuous illumination. More attractively, the PM6:4Y-BO blend film exhibits more notable elastic deformation and elastic modulus than the PM6:PY-BO blend, yielding good bending stability and performance rebound characteristics for the resulting flexible OSCs. These discoveries clearly demonstrate the great promise of tetramer 4Y-BO over polymer acceptors not only on photovoltaic performance but also on the critical device thermal stability and mechanical flexibility.

## Results

### Molecular design, synthesis and characterizations

**Fig. 1:**
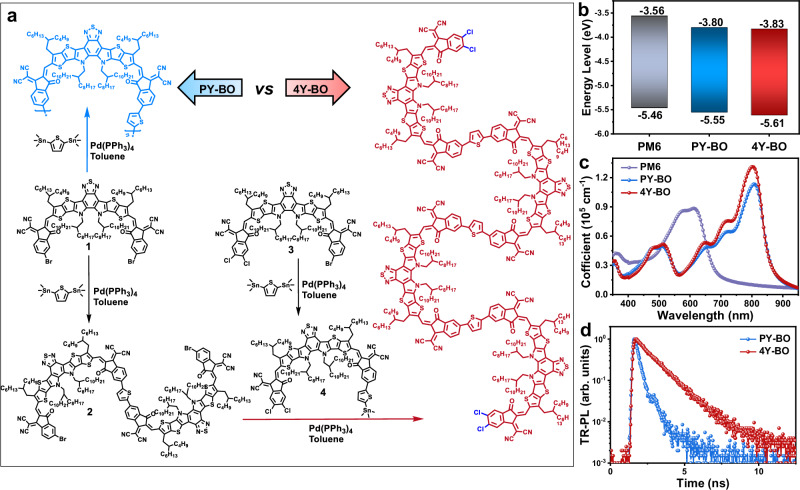
Synthetic approaches and basic characteristics of polymer PY-BO and tetramer 4Y-BO. **a** Synthesis route of polymer acceptor PY-BO and tetramer acceptor 4Y-BO. **b** Highest occupied molecular orbital (HOMO) and lowest unoccupied molecular orbital (LUMO) energies of PY-BO and 4Y-BO. **c** Absorption coefficients of donor PM6, acceptors PY-BO and 4Y-BO films. **d** Time-resolved photoluminescence (TR-PL) spectra of PY-BO and 4Y-BO films.

The synthetic approaches of polymer acceptor PY-BO and tetramer acceptor 4Y-BO are displayed in Fig. 1a. The details of the analysis are provided in Supplementary Methods. The polymer acceptor PY-BO was acquired through Stille polymerization between the monomer 1 and thiophene linker, followed by Soxhlet extraction and obtained by concentrating the chloroform component. Finally, PY-BO was synthesized with a moderate yield of 63%, attributed to the large fraction of low molecular weight components extracted by hexane solvent. As expected, the *M*_n_ of PY-BO based on high-temperature gel permeation chromatography (HT-GPC) measurement is determined to be 9.96 kDa with a polydispersity of 1.88 (Supplementary Fig. 1), both of which are in line with the previous literatures of typical PSMAs. The tetramer acceptor 4Y-BO was obtained by three Stille coupling steps, as depicted in Fig. 1a. Initially, we synthesized the brominated dimer 2 by coupling monomer 1 and thiophene linker with a largely unbalanced molar ratio. As a result, dimer 2 was isolated with a yield of 65% and the excessive monomer 1 can be readily recycled. Likewise, the asymmetrical intermediate compound 4 was received by Stille coupling between compound 3 and the thiophene linker and was employed for the next procedure without further purification. At last, the tetramer 4Y-BO was acquired by coupling the dimer 2 and intermediate 4 with a notable yield of about 80%. The chemical structures and molecular weight of 4Y-BO are confirmed by nuclear magnetic resonance (NMR) spectrum and MALDI-TOF mass spectrometry (Supplementary Figs. 2–4). The identical molecular backbones and side chains of the polymer and tetramer allow their comparison more reliable. We subsequently extract the tetramer component of polymer PY-BO (chemical structure in Supplementary Fig. 5) and the oligomer 4Y-BO for theoretical simulations. As displayed in Supplementary Fig. 6, both the two molecules show comparable and basically flat molecular configurations, enabling potentially decent molecular packing and charge hoping capacity. The 4Y-BO π-skeleton shows more positive electrostatic potential (ESP) distribution than the counterpart (Supplementary Fig. 7), which is conducive to strengthen the interactions of the acceptor with a donor with globally negative ESP distributions (Supplementary Fig. 8). Internal reorganization energy (*λ*_int_), which is a metrics of energetic cost for structural relaxation after ionization, are calculated and collected in Table 1. It is notable that the 4Y-BO receives collectively reduced electron internal reorganization energy (*λ*_int,e_) and hole internal reorganization energy (*λ*_int,h_) than those of PY-BO, revealing faster charge transfer (particular for electrons) rate and larger charge mobilities according to the Marcus-Levich-Jortner’s formalism^35^. Additionally, based on average local ionization energy (ALIE) maps (Supplementary Fig. 9), the 4Y-BO is equipped with a mildly higher vertical ionization energy (VIE) of 6048 meV compared to PY-BO (6008 meV), favoring more efficient electron acquisition from a donor. These results support the favorable semiconductor property of 4Y-BO serving as an electron acceptor.

**Table 1:** Optical properties, frontier energy levels, and simulated results of PY-BO and tetramer 4Y-BO

Acceptor	Molecular weight	*λ*_max_ (nm)		*E*_g_^opt^	*ε*_film_	HOMO/LUMO	*λ*_int,e_	*λ*_int,h_	VIE
	(kDa)	solution	film	(eV)	(10^5^ cm−^1^)	(eV)^CV^	(meV)	(meV)	(meV)
PY-BO	9.96	795	807	1.41	1.13	− 5.55/− 3.80	28.50	41.72	6008
4Y-BO	7.36	765	804	1.41	1.31	− 5.61/− 3.83	27.38	40.91	6048

*λ*_max_ represents the wavelength of the absorption peak. *E*_g_^opt^ calculated from the onset wavelength of the film absorption spectra. *ε*_film_ represents the maximum extinction coefficient of the films. HOMO/LUMO levels obtained from CV measurements. *λ*_int,e_ and *λ*_int,h_ represent electron and hole internal reorganization energy, respectively. VIE represents vertical ionization energy.

Frontier molecular orbitals of the polymer and tetramer are investigated by cyclic voltammetry (CV) measurements (Supplementary Fig. 10). As suggested in Fig. 1b, the 4Y-BO exhibits collectively and slightly deeper highest occupied molecular orbital (HOMO) and lowest unoccupied molecular orbital (LUMO) energy levels than those of PY-BO. Subsequently, we used density functional theory (DFT) to calculate the orbital distribution as shown in Supplementary Table 1. The tetramer contains four occupied frontier orbitals with similar energy, distributed on different Y-units. Similarly, four unoccupied frontier orbitals with similar energy are distributed on different repeat units. The molecular orbital distribution shows that the frontier orbitals of the monomer mix with each other to form the additional molecular orbitals following the formation of the tetramer, which shows a consistent trend with the experimental results.

Absorption profiles in dilute chloroform solutions and as thin films are given in Supplementary Fig. 11 and Fig. 1c, respectively, with relevant data summarized in Table 1. It is interesting that the two solutions show dramatically different absorption profiles with the maximum wavelengths of PY-BO and 4Y-BO determined to be 795 and 765 nm, respectively. This uncovers the remarkable pre-aggregation behavior of the polymer PY-BO stemming from significant interchain entanglements. In contrast, the well-structured tetramer molecules are distributed uniformly in the dilute solution. Interestingly, both the polymer and tetramer receive comparable absorption profiles in neat films, sharing the identical optical bandgap (*E*_g_^opt^) of 1.41 eV. The limited variation of the maximum wavelength of PY-BO (12 nm) further confirms its excessive pre-aggregation property in solution. Notably, the 4Y-BO offers a large bathochromic shift of the maximum wavelength by 39 nm from solution to film absorptions. This firstly suggests the limited pre-aggregation of 4Y-BO in solution, and secondly the remarkably enhanced molecular ordering in film state. As Fig. 1c depicts, the 4Y-BO film also shows a greater maximum extinction coefficient (*ε*) of 1.31  × 10^5 ^cm^−1^ than that of the PY-BO (1.13 × 10^5 ^cm^−1^), offering a stronger capacity to harvest solar photons. In comparison, the typical polymer donor PM6 exhibits a moderate maximum *ε*_film_ of 0.90 × 10^5 ^cm^−^^1^. We further investigated the excitation properties of PY-BO and 4Y-BO based on the CAM-B3LYP/6-311 g(d,p)/polarizable continuum model method. As shown in Supplementary Fig. 12, both the absorption spectra of PY-BO and 4Y-BO are dominated by the S0 → S1 transition. The oscillator strength (*f*) of the S0 → S1 transition for PY-BO and 4Y-BO are 7.7890 and 8.8248, respectively, which indicates better light absorption of 4Y-BO than that of PY-BO. To analyze the essential characteristics of electronic transitions, we further examined the contribution of each pair of molecular orbital transitions to the S0 → S1 transition (Supplementary Fig. 13 and Supplementary Table 2). Results show that all four occupied frontier orbital (H-3, H-2, H-1, H) and the four unoccupied frontier orbital (L, L + 1, L + 2, L + 3) contribute to the electron transition from S0 to S1 state, and the contribution ranges from 2 to 20%. The total contribution accounts for over 70%. The detailed frontier orbital distribution was further investigated. Sr (ranges from 0 to 1) represents the overlap between the electron and hole, and the Sr closer to 1 indicates the greater overlap. As the results confirmed, the Sr of S0 → S1 transition for 4Y-BO is slightly higher than that of PY-BO, and both of them are close to 0.75. The large overlap means high transition density between the involved molecular orbitals, thus the large transition oscillator strength. Therefore, the tetramer should have improved light absorption ability and consistent well with our experimental results. We further investigated the steady-state fluorescence spectra of the polymer and tetramer acceptors, demonstrating similar spectra with a maximum wavelength located at ∼844 nm (Supplementary Fig. 14). Interestingly, 4Y-BO has notably stronger emission with a maximum intensity of 3.80 folds higher than that of PY-BO. Based on time-resolved photoluminescence (TR-PL) spectra (Fig. 1d), the 4Y-BO film exerts a significantly delayed decay plot than that of PY-BO, yielding a greater fluorescent lifetime (*τ*) of 1.15 ns than the PY-BO film with a small *τ* of 0.28 ns. The longer excitation lifetime of 4Y-BO could be helpful in suppressing the nonradiative recombination pathway, according to previous studies^36–38^, and should be partly attributed to its restrained molecular disorder.

### Molecular aggregation and film formation dynamics

**Fig. 2:**
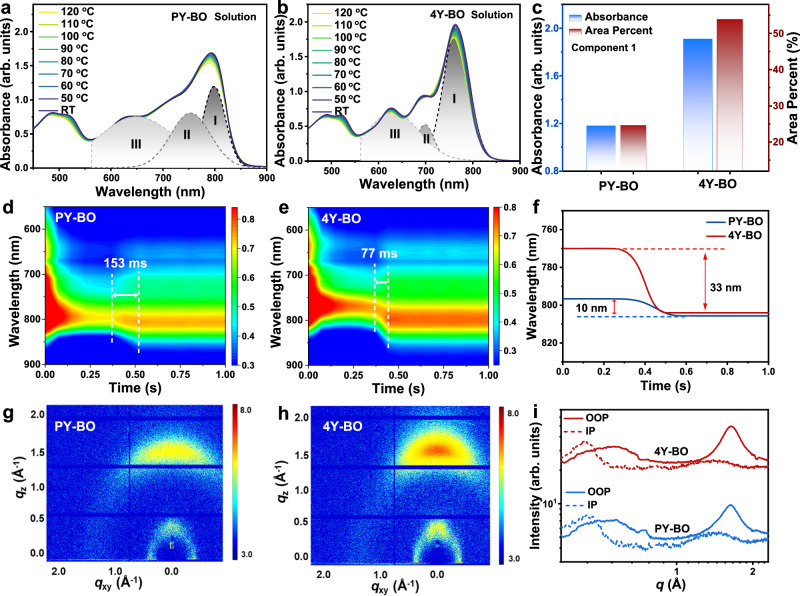
Film-forming kinetics studies of the polymer and tetramer acceptors. **a**–**c** Absorption spectra of PY-BO and 4Y-BO in Chlorobenzene (CB) dilute solutions (1 × 10^−3 ^mg ml^−^^1^) at varied temperatures. **d**–**f** 2D time-resolved in situ absorption spectra and maximum peak variations of PY-BO and 4Y-BO during the solution-to-film transformation stage. **g**–**i** 2D Grazing-incidence wide-angle X-ray scattering (GIWAXS) patterns and corresponding line-cut profiles of PY-BO and 4Y-BO films, solid and dotted curves stand for out of plane (OOP) and in-plane (IP) directions, respectively.

In order to systematically present the differences in aggregation and stacking characteristics of the polymer and tetramer acceptors, we conducted a detailed comparison of the two materials in dilute solutions, solution-to-film transformation stage, and the final film stages, respectively. Firstly, the pre-aggregation property was investigated by temperature-dependent absorption measurements at room temperature to 120 ^o^C in dilute chlorobenzene solutions. As depicted in Fig. 2a and b, both the two solutions receive negligible variations of absorption profiles at varied temperatures, suggesting equally strong pre-aggregation capacity and should be reasonable given the inherent entanglements of polymer acceptor and fairly extended π-conjugation of the tetramer with an overall decent planar configuration. Besides, the 4Y-BO shows mildly enlarged variations of wavelength and intensity of the (0-0) absorption peaks than those of PY-BO (Supplementary Fig. 15), which indicates a moderately reduced pre-aggregation behavior and basically supports the discovery in chloroform solutions as discussed above. The slightly stronger pre-aggregation of PY-BO can be attributed to the excessive entanglement of larger molecular-weight components. In addition, the absorption profiles at between 550–850 nm can be generally divided into three separate absorptions as annotated in Fig. 2a and b. Among them, components I with maximum absorption wavelengths of 793 nm (PY-BO) and 764 nm (4Y-BO) should be attributed to the contributions of *J*-aggregations^39–41^, which are preferable to afford compact molecular packing in resultant film states. Figure 2c compares the maximum absorbance and area percent of component I for the polymer and tetramer profiles, with relevant data summarized in Supplementary Table 3. In the premise of identical mass concentration, component I of 4Y-BO delivers 1.62 folds of maximum absorbance relative to that of PY-BO. Meanwhile, the area percents of component I exhibit a conspicuous boost from PY-BO to 4Y-BO, with percents dramatically increased from 24.56% to 53.93%. These results suggest a larger fraction of advantageous *J*-aggregates in the tetramer solution, in spite of the more intense pre-aggregation property of the polymer. The significantly different pre-aggregation propensity should be attributed to the more orderly molecular structure of 4Y-BO and, hence organized self-assembly property.

In situ, absorption spectra were further measured to gain an in-depth investigation about the film-forming kinetics of the polymer and tetramer acceptors. 2D time-resolved absorption patterns of PY-BO and 4Y-BO spin-coating from chloroform solutions are provided in Fig. 2d and e, respectively. In general, the film-forming procedure includes three individual stages including solution state, solution-to-film transformation state, and post-film state^42–44^. In the transformation stage, the solution reaches the critical concentration through continuous solvent evaporation, and the molecules begin to aggregate and gradually grow into ordered aggregates on the substrate. During this period, the absorption intensity would receive a first decrease and then a significant increase along with a redshift for the spectrum. The polymer PY-BO affords a transformation duration of 153 ms with a small maximum peak variation of 10 nm. In stark comparison, the tetramer 4Y-BO demonstrates a notably reduced transformation duration of 77 ms along with a remarkable peak variation of 33 nm (Fig. 2f). The distinguishing absorption variations during the transformation stage should be correlated with the intrinsically different pre-aggregation behaviors of the polymer and tetramer, as supported by absorptions in solutions (Supplementary Fig. 11) and films (Fig. 1c). Based on the transformation duration, the solution-to-film transformation speed of the PY-BO and 4Y-BO can be determined to be 6.54 and 12.99 s^–1^, respectively. Intuitively, the stronger pre-aggregation of polymer PY-BO should facilitate the transformation process, which, however, is seriously delayed in this context. The prolonged deposition duration of PY-BO should be dominated by the complex components included in the polymer with a low polymerization degree. The heavily blended oligomers with different molecular weights could result in contrasting crystallization kinetics, leaving an overall extended transformation duration. On the contrary, the well-defined molecular structure of tetramer 4Y-BO promotes homogeneous crystallization in concentrated solution, which can be partly assisted by the halogen modification of 4Y-BO terminals as well. During the film-forming process from solution to film state, the intermolecular spacing decreases. Under this circumstance, intermolecular interactions can have a dominating impact on film formation and crystallinity. To further clarify this point, we calculated the intermolecular interactions for the polymer and tetramer. As depicted in Supplementary Fig. 16, the complexation energy between 4Y-BO (− 39.92 kcal mol^−^^1^) and 4Y-BO is notably higher than that between PY-BO and PY-BO (− 35.50 kcal mol^−^^1^), which could provide the greater driving force for molecule self-assembly and thus larger absorption redshift. The widely divergent solution-to-film transformation kinetics could generate different molecular aggregations in the following post-film stage. Therewith, we conducted grazing incidence wide-angle X-ray scattering (GIWAXS) measurements to explore the molecular stacking and orientations of PY-BO and 4Y-BO films. Figures 2g and h display the 2D-GIWAXS patterns, with corresponding line-cut profiles shown in Fig. 2i. Both the polymer and tetramer films show dominating face-on orientation. Apparently, the 4Y-BO possesses a more noticeable (010) π-π diffraction along the out-of-plane (OOP), which is conducive to transporting free charges more efficiently along the vertical azimuth. The *q* locations, stacking distances (*d*), and crystal coherence lengths (CCLs) of the (010) diffraction along the OOP direction and the (100) diffractions along the in-plane (IP) direction are summarized in Supplementary Table 4. In spite of the similar stacking distances with PY-BO, the 4Y-BO delivers collectively improved CCLs from 41.34 to 54.17 Å for (100) diffraction, and 19.16 to 24.35 Å for (010) diffraction. These data support the stronger crystallization property of 4Y-BO, which is notably attributed to its film-forming characteristic as described above. Additionally, we measured the electron mobilities (*μ*_e_) of the two neat films based on the space-charge-limited current (SCLC) method^45^. As provided in Supplementary Fig. 17 and Supplementary Table 5, the 4Y-BO film gains markedly greater *μ*_e_ of 1.02 × 10^−^^4 ^cm^2 ^V^−^^1^ s^−^^1^ than that of PYBO (0.69 × 10^−^^4 ^cm^2 ^V^−^^1^ s^−^^1^). The preponderant electron transport capacity of 4Y-BO should be attributed to the synergy of the molecular itself (lower *λ*_int,e_) and preferable molecular orientation.

### Photovoltaic properties of OSCs

**Fig. 3:**
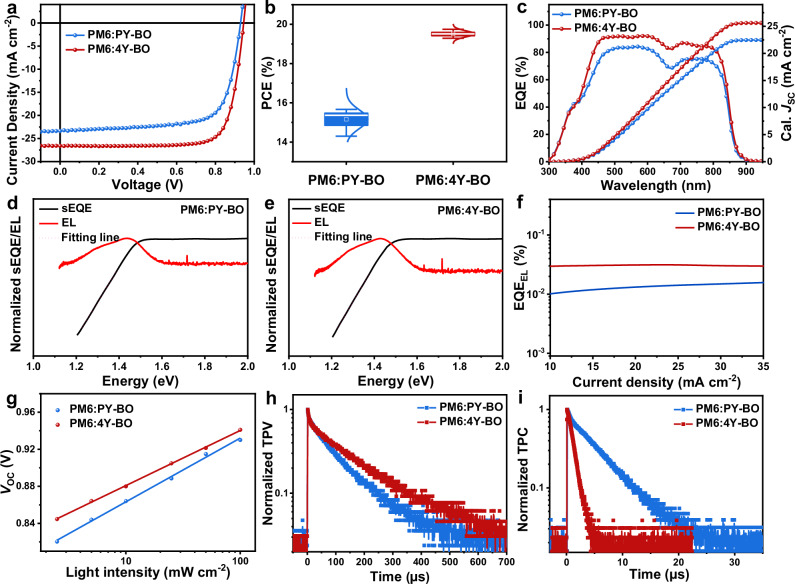
Photovoltaic performance of solar cells. **a** *J*-*V* plot of PM6:PY-BO and PM6:4Y-BO optimal OSCs. **b** Statistical distributions of PCEs based on no less than ten individual devices. **c** External quantum efficiency (EQE) profiles of the corresponding optimal devices. **d**–**f** Normalized highly-sensitive external quantum efficiency (sEQE), electroluminescence (EL) spectra, and electroluminescence quantum efficiency (EQE_EL_) of the PM6:PY-BO and PM6:4Y-BO-based devices. **g** Plots of *V*_OC_ *versus* light intensity. **h**, **i** Transient photovoltage (TPV) and transient photocurrent (TPC) plots of solar cells.

We then investigated the photovoltaic performance of the polymer and tetramer based on the conventional device structure of ITO/PEDOT: PSS/active layer/PDINN/Ag, where polymer PM6 was employed as the donor. The detailed device manufacturing and optimizing processes were described in Supplementary Methods, with detailed data collected in Supplementary Table 6. Figure 3a displays the current density-voltage (*J*-*V*) plots of optimized OSCs. As shown in Table 2, the PM6:PY-BO based devices yield a moderate PCE of 15.66%, with open circuit voltage (*V*_OC_) of 0.929 V, *J*_SC_ of 23.33 mA cm^−2^, and FF of 72.26%, and the efficiency is in line with those based on typical polymer acceptors in previous literatures^46–49^. Excitingly, the PM6:4Y-BO based OSC showcase an impressive champion PCE up to 19.75%, with collectively improved *V*_OC_ of 0.944 V, *J*_SC_ of 26.59 mA cm^−^^2^, and FF of 78.63%. More attractively, the efficiency of PM6:4Y-BO ranks the highest among OSCs based on oligomer acceptors so far (Supplementary Table 7). Notably, the champion efficiency of the PM6:4Y-BO device has been certified by the National Center of Inspection on Solar Photovoltaic Products Quality (CPVT), China. The certified PCE of PM6:4Y-BO solar cell reached 19.58%, with *V*_OC_ of 0.940 V, *J*_SC_ of 26.94 mA cm^−^^2^, and FF of 77.33% (Table 2 and Supplementary Fig. 18). The greater performance of PM6:4Y-BO should be attributed to its good phase-separations and hence better exciton/charge management, which will be discussed in detail later. Figure 3b illustrates the statistical distributions of PCEs based on at least ten individual cells. Compared with PM6:PY-BO, the PM6:4Y-BO based devices show significantly narrowed batch-to-batch variations, highlighting the reliability of device parameters and should be correlated with the defined molecular structure of 4Y-BO. External quantum efficiency (EQE) spectra (Fig. 3c) suggest a global enhancement of photon utilization in PM6:4Y-BO solar cells, well-supporting the more preferable *J*_SC_ compared to that of PM6:PY-BO, which is basically consistent with the absorption spectra of the blend films (Supplementary Fig. 19). The integrated *J*_EQE_s are calculated to be 22.47 mA cm^−^^2^ and 25.62 mA cm^−^^2^ for PY-BO and 4Y-BO based devices, respectively, both of which are consistent with the recorded *J*_SC_s with limited deviations of about 4%.

**Table 2:** Photovoltaic parameters of PM6:PY-BO and PM6:4Y-BO based OSCs under the illumination of AM 1.5 G (100 mW cm^–^^2^)

Active layer	*V*_OC_(V)	*J*_SC_ (mA cm^–^^2^)	*J*_EQE_ (mA cm^–^^2^)	FF (%)	PCE^a^ (%)	*E*_loss_ (eV)
PM6:PY-BO	0.929 (0.926 ± 0.003)	23.33 (23.19 ± 0.41)	22.47	72.26 (70.25 ± 0.73)	15.66 (15.16 ± 0.57)	0.556
PM6:4Y-BO	0.944 (0.943 ± 0.003)	26.59 (26.58 ± 0.21)	25.62	78.63 (77.96 ± 0.52)	19.75 (19.55 ± 0.23)	0.534
PM6:4Y-BO^b^	0.940	26.94	–	77.33	19.58	–

^a^Average parameters with standard deviations obtained from at least ten devices.

^b^Certificated by the National Center of Inspection on Solar Photovoltaic Products Quality (CPVT).

*J*_EQE_ represents the integral current of EQE curves. *E*_loss_ represents the energy loss of the devices.

In addition, we highlight the greater *V*_OC_ of the PM6:4Y-BO device in spite of the slightly deeper LUMO level of 4Y-BO (Table 1). In order to clarify this point, we move to explore the energy loss (*E*_loss_) of solar cells. Highly sensitive external quantum efficiency (sEQE), electroluminescence (EL) spectra, and electroluminescence quantum efficiency (EQE_EL_) were measured as depicted in Fig. 3d–f, with detailed parameters collected in Supplementary Table 8. The *E*_g_s of PM6:PY-BO and PM6:4Y-BO devices are determined to be 1.485 eV and 1.478 eV, respectively. The *E*_loss_ can be described as three parts based on the detailed balance theory: ${E}_{{loss}}=\Delta {E}_{1}+\Delta {E}_{2}+\Delta {E}_{3}$, where Δ*E*_1_ is constrained by the Shockley-Queisser limit, Δ*E*_2_ is the inevitable radiative energy loss, and Δ*E*_3_ is the non-radiative energy loss^50^. In this scenario, the Δ*E*_1_ of PM6:PY-BO and PM6:4Y-BO-based devices are 0.271 and 0.270 eV, and Δ*E*_2_ are 0.058 and 0.057 eV, respectively. It is worth noting that the Δ*E*_3_ of the two solar cells, obtained from EQE_EL_ spectra, are markedly different with 0.227 and 0.207 eV for PM6:PY-BO and PM6:4Y-BO devices, respectively. Urbach energy (*E*_U_) is further calculated by fitting the sEQE spectra, yielding a smaller *E*_U_ of 23.3 meV for the PM6:4Y-BO device than that of PM6:PY-BO (24.3 meV). This result uncovers that the improved molecular stacking of 4Y-BO ulteriorly suppresses energetic disorder, which affords fewer energy traps and scattering centers, and effectively reduces non-radiative recombination for higher EQE_EL_. In order to further expound the reduced non-radiative energy loss of the PM6:4Y-BO device, we measured the photoluminescence quantum yield (PLQY) of PY-BO and 4Y-BO neat films. As shown in Supplementary Fig. 20, the PLQY of PY-BO and 4Y-BO are determined to be 1.53% and 8.26%, respectively. The increased PLQY of 4Y-BO should be one important factor for the reduction of Δ*E*_nr_^51^. Besides, the lower Δ*E*_nr_ of the PM6:4Y-BO device should be partly attributed to the more ordered molecular stacking and appropriate phase separations, which promote charge transfer and separation and reduce charge accumulation and recombination at the interfaces. As a result, the overall *E*_loss_ of PM6:4Y-BO device (0.534 eV) is smaller than that of PM6:PY-BO (0.556 eV), well supporting the greater *V*_OC_ of PM6:4Y-BO solar cells.

### Charge generation and transport properties

In the following, we focus on the investigation of exciton and charge behaviors. Photocurrent density (*J*_ph_) *versus* effective voltage (*V*_eff_) plots were carried out to explore the exciton dissociation, charge generation, and collection properties. As depicted in Supplementary Fig. 21 and Supplementary Table 9, the PM6:4Y-BO-based device affords simultaneous increases of exciton dissociation probability (*P*_diss_) and charge collection probability (*P*_coll_) than those of PM6:PY-BO, further supporting the achievement of higher *J*_SC_ and FF. Subsequently, we measured the relationships of *J*_SC_ and *V*_OC_ *versus* light illumination intensity to probe the charge recombination mechanism. As indicated in Supplementary Fig. 22, both the two OSCs reveal equally negligible bimolecular charge recombination considering the uniform pre-exponential factor (*s*) of 0.98 and 0.99^52,53^. Whereas, according to the equation of ${V}_{OC}\propto \frac{{{{\rm{n}}}}{kT}}{q}{{{{\mathrm{ln}}}} ({P}_{light})}$, where *k* represents the Boltzmann constant, *T* represents the Kelvin temperature, and *q* represents the elementary charge^54^, the PM6:4Y-BO device shows a much smaller *n* value (1.02 *kT*/*q*) than that of PM6:PY-BO (1.17 *kT*/*q*) (Fig. 3g). This result suggests largely inhibited trap-assisted recombination occurred in PM6:4Y-BO device. TR-PL measurements were applied to probe the charge transfer in blend films (Supplementary Fig. 23). As a result, the PM6:4Y-BO shows a slightly smaller fluorescence lifetime of 175 ps compared to the PM6:PY-BO blend (*τ* = 192 ps), indicating accelerated charge transfer between PM6 and 4Y-BO. In order to elucidate the charge extraction and recombination dynamics, we investigated transient photovoltage (TPV) and transient photocurrent (TPC) decays. By fitting the TPV decays (Fig. 3h), the PM6:4Y-BO based device affords noticeably prolonged charge carrier lifetimes than the PM6:PY-BO device (138.7 μs *vs* 93.5 μs), which is conducive to retarding the charge recombination. Meanwhile, the PM6:4Y-BO device achieves ultrafast charge extraction with an observably shorter charge extraction time of 1.02 μs than that of PM6:PY-BO (5.03 μs), obtained by fitting the TPC decays (Fig. 3i). The accelerated charge extraction kinetics should be partly attributed to the more ordered molecular packing of 4Y-BO molecules. Profited from the collectively improved charge generation, extraction, and recombination properties, the PM6:4Y-BO device recorded greater and more uniform hole and electron charge transport capacity, as supported by mobilities obtained based on the SCLC method (Supplementary Fig. 24 and Supplementary Table 5).

**Fig. 4:**
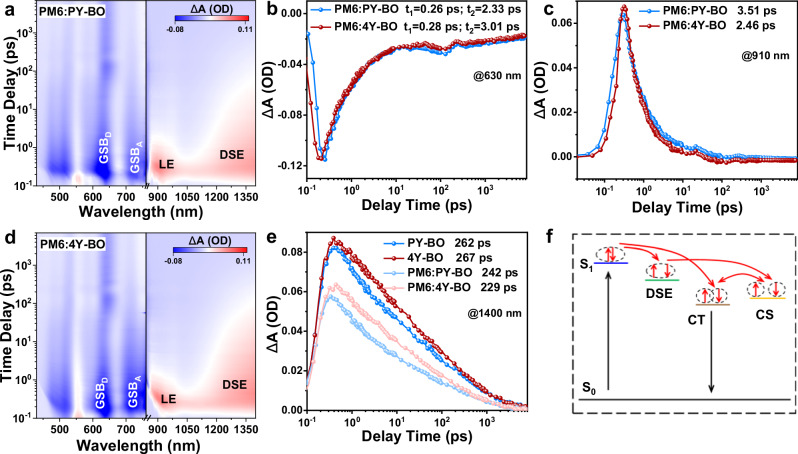
fs-TA studies and charge transfer kinetics. **a** 2D pattern of fs-transient absorption (fs-TA) spectra of PM6:PY-BO blend films. **b** TA traces of ground-state-bleach (GSB) species probed at 630 nm. **c** TA traces of singlet localized exciton (LE) species probed at 910 nm. **d** 2D patterns of fs-TA spectra of PM6:4Y-BO blend films. **e** TA traces of delocalized singlet exciton (DSE) species of the neat acceptors and blend films probed at 910 nm. **f** Proposed charge transfer pathways in the employed solar cells, CS and CT represent charge separation generation and charge transfer state.

Femtosecond transient spectroscopy (fs-TA) was further applied to set forth the charge transfer kinetics, where the acceptors were selectively excited with a pump beam at 780 nm. Figures 4a and d describe the 2D patterns of fs-TA spectra of PM6:PY-BO and PM6:4Y-BO blend films, with line-cut profiles at indicated decay times provided in Supplementary Figs. 25 and 26. In general, the two blends show comparable absorption patterns with the negative signals at ∼750 nm belong to ground-state-bleach (GSB) absorptions of acceptor PY-BO and 4Y-BO. The negative species that appeared at ∼630 nm can be assigned to the GSB absorption of donor PM6. This affirms the efficient charge transfer (HT) from acceptor to donor, in that the acceptor is solely excited in the blend. By fitting the GSB of PM6 with a biexponential function, the ultrafast exciton dissociation duration (t_1_) at donor/acceptor heterojunction and the relatively large exciton diffusion time (t_2_) toward the interface can be extracted. As depicted in Fig. 4b, both the two blends have an equally ultrafast exciton dissociation process. However, the PM6:4Y-BO receives a moderately prolonged exciton diffusion duration, which is probably correlated with the enhanced film crystallinity and larger domains as suggested in the following morphology studies. As noted, both the two blends give rise to a positive signal at ∼910 nm, which should be attributed to the absorption of singlet localized exciton (LE). Figure 4c shows the decay kinetics of LE in the two systems. It is evident that the LE of the PM6:4Y-BO blend reveals an accelerated decay compared to that of PM6:PY-BO, affording LE lifetimes of 2.46 and 3.51 ps, respectively. Taking into account the lower geminate recombination inside the PM6:4Y-BO device (Fig. 3d), the reduced LE lifetime of the blend should be attributed to more efficient charge separation (CS) generation through charge transfer (CT) state or other intermediate states. Besides of the LE species, there is a more prominent positive signal with a considerable timescale that emerged at ∼1350 nm. According to the previous report, this notable absorption should be arising from delocalized singlet exciton (DSE), which is a pivotal intermediate to split excitons and yield CS species without the participation of heterojunction CT state. As displayed in Fig. 4e, the DSE states can be formed at ultrafast timescale of 0.3 ∼ 0.4 ps, about 0.5 ∼ 1.0 ps behind the generation of LE states. Whereas, the DSE exhibits an apparently long-lived characteristic compared to the LE. In order to explore the evolutions of DSE in neat acceptors and blend systems, the fs-TA spectra of neat PY-BO and 4Y-BO are further measured with results collected in Supplementary Figs. 27–29. Figure 4e depicts and compares the DSE decay kinetics in different films. Apparently, the neat acceptors of PY-BO and 4Y-BO have more conspicuous DSE signals with longer lifetimes of 262 and 267 ps, respectively. From neat acceptor to blend film, the notable DSE species are sharply quenched, suggesting the participation of DSE in the period of charge generation. In particular, the PM6:4Y-BO blend offers a more efficient DSE involvement with a smaller DSE lifetime of 229 ps compared to that of PM6:PY-BO (242 ps). Figure 4f describes the proposed exciton splitting and charge generation pathway. The short-lived Frenkel excitons upon photoexcitation can follow routine LE → CT → CS charge generation mechanism. Alternatively, they can also undergo the LE → DSE → CS pathway to generate free charges without the intervention of interfacial CT states^55–57^. The self-promoted charge generation mechanism can reduce the disadvantageous CT relaxation mediated by ^1^CT/^3^CT internal conversion and resultant molecular triplet formation.

### Blend film morphology characterization

**Fig. 5:**
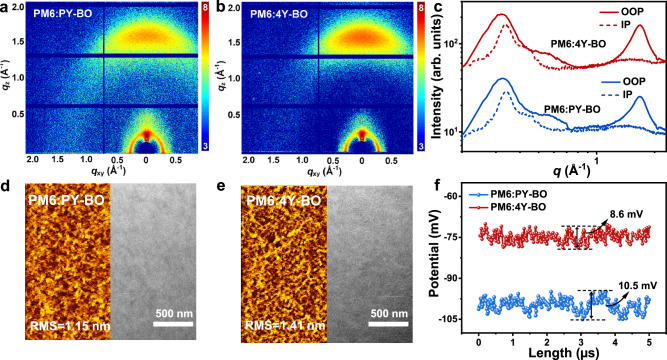
Heterojunction textures of blend films. **a**, **b** 2D GIWAXS patterns of PM6:PY-BO and PM6:4Y-BO blend films. **c** Line-cut profiles of GIWAXS studies, solid and dotted curves stand for out-of-plane (OOP) and in-plane (IP) directions, respectively. **d**, **e** Atomic force microscopy (AFM) (2.5 × 5.0 μm), and transmission electron microscope (TEM) photos of the blend films, RMS represents root mean square. **f** Surface potential distributions along indicated lines extracted from Kelvin probe force microscopy (KPFM) maps.

As well-acknowledged, nanoscale heterojunction textures play a crucial factor in regulating the exciton and charge behaviors. The crystalline property and molecular arrangement of PM6:PY-BO and PM6:4Y-BO blend films are investigated through GIWAXS studies, with 2D diffraction patterns and corresponding line-cut profiles presented in Fig. 5a–c. Both the two blends take on dominating face-on molecular orientation with a notable (010) diffraction plot appeared along the OOP direction. In spite of the comparable *q*_z_ location at ∼1.68 Å^−1^, the (010) diffraction intensity of PM6:4Y-BO is largely stronger than that of PM6:PY-BO, accompanied by a greater CCL of 38.1 Å than that of the latter blend (32.2 Å). The improved film crystallinity is expected to provide a more orderly transport channel for free charges, as supported by SCLC measurements (Supplementary Fig. 24). Considering that both PM6 and acceptors show almost overlapped *q*_z_ locations of ∼1.70 Å^−^^1^ for their intrinsic (010) diffractions (Fig. 2g–i and Supplementary Fig. 30), we employed the well-defined (100) diffractions along IP direction for probing the crystallinity property of the donor and acceptor components separately. As Supplementary Fig. 31 shows, the (100) diffractions of both blends can be perfectly resolved into two splitting diffraction components, with relevant data including *q*_xy_ locations and CCLs summarized in Supplementary Table 10. Based on the diffraction data of neat PM6 and acceptors, the split components located at *q*_xy_ = 0.337 Å^−1^ and *q*_xy_ ∼ 0.40 Å^−^^1^ can be assigned to donor PM6 and PY-BO/4Y-BO, respectively. As described in Fig. 5c, the PM6 components inside the two blends exhibit similar and equally considerable CCL of 119 and 105 Å. However, the polymer PY-BO and tetramer 4Y-BO show contrasting crystallinity in their respective blend film. The 4Y-BO affords a notably larger CCL of 86 Å, in relative to the PY-BO (31 Å). Thus, we can conclude that the improved blend crystallinity of PM6:4Y-BO is primarily contributed by the acceptor 4Y-BO, which has more ordered stacking than polymer PY-BO. Based on the CCLs of the donor and acceptor, apparently, the PM6:4Y-BO demonstrates a more uniform crystallinity with CCL_PM6/4Y-BO_ of 1.22, which partly contributes to the more balanced hole and electron mobilities, relative to PM6:PY-BO blend with a large CCL_PM6/PY-BO_ of 3.84. In order to further understand the evolutions of molecular packing in blend films, we performed DFT calculations on the intermolecular interactions of PM6/PY-BO and PM6/4Y-BO pairs. As shown in Supplementary Fig. 32, the PM6/4Y-BO pair shows a greater complexation energy of − 57.50 kcal mol^−^^1^ than that of PM6/PY-BO (− 55.10 kcal mol^−^^1^), which should be beneficial for appropriate phase-separations and thus improved charge generation property. In addition, by combining the strong complexation energy within the 4Y-BO and 4Y-BO, the crystallinity of the donor and acceptor in blend film can be improved, which is consistent with the experimental results.

The global morphology and phase separations are studied through atomic force microscopy (AFM) and transmission electron microscope (TEM), with results displayed in Fig. 5d and e. Both the two blend films show fibrous surface morphology. However, a mildly higher root mean square (RMS) roughness of 1.41 nm observed from PM6:4Y-BO than that of PM6:PY-BO (RMS = 1.15 nm), which should be attributed to the enhanced molecular aggregation of 4Y-BO than the PY-BO component as confirmed above. TEM photos compare the phase separations more evidently. The PM6:4Y-BO shows an astonishing microstructure with plenty of well-defined crystalline fibers, which are highly preferred for bulk heterojunctions to efficiently transport free charges. Flory-Huggins interaction parameters (χ) are further calculated based on the surface energies (*γ*_s_) of PM6, PY-BO, and 4Y-BO, obtained from contact angles measurements. As Supplementary Fig. 33 and Supplementary Table 11 suggest, the 4Y-BO receives improved compatibility with PM6 compared to that between PY-BO and PM6, which should be partly attributed to the greater intermolecular interactions of PM6/4Y-BO pair. The better compatibility between 4Y-BO and PM6 is beneficial for more uniform and appropriate heterojunction textures as disclosed above.

Given the contrasting heterojunction morphologies, we further applied Kelvin probe force microscopy (KPFM) to probe the surface contact potential (SCP) for a better understanding of the high *J*_SC_ and efficiency of PM6:4Y-BO based OSCs. KPFM maps of the two blend films are provided in Supplementary Fig. 34, with corresponding SCP distributions along indicated lines presented in Fig. 5f. For one thing, the PM6:4Y-BO yields a higher average contact potential of − 75 mV compared to that of PM6:PY-BO (− 99 mV), which is conducive to enhancing built-in potential and driving force for exciton dissociation and charge generation^58,59^. In addition, the contact potential of PM6:4Y-BO is more uniformly distributed with a fluctuation of ∼8.6 mV than the PM6:PY-BO blend with a larger fluctuation of ∼10.5 mV. In spite of the greater RMS of PM6:4Y-BO surface, the more uniform SCP distribution should be attributed to the improved film crystallinity with larger CCLs. In situ absorption spectra of the two blend films (Supplementary Figs. 35 and 36) afford comparable evolutions with those of neat acceptors (Fig. 2d–f), which further confirms the dominating regulation of acceptor components on blend textures.

### Thermal and mechanical stability characteristics

**Fig. 6:**
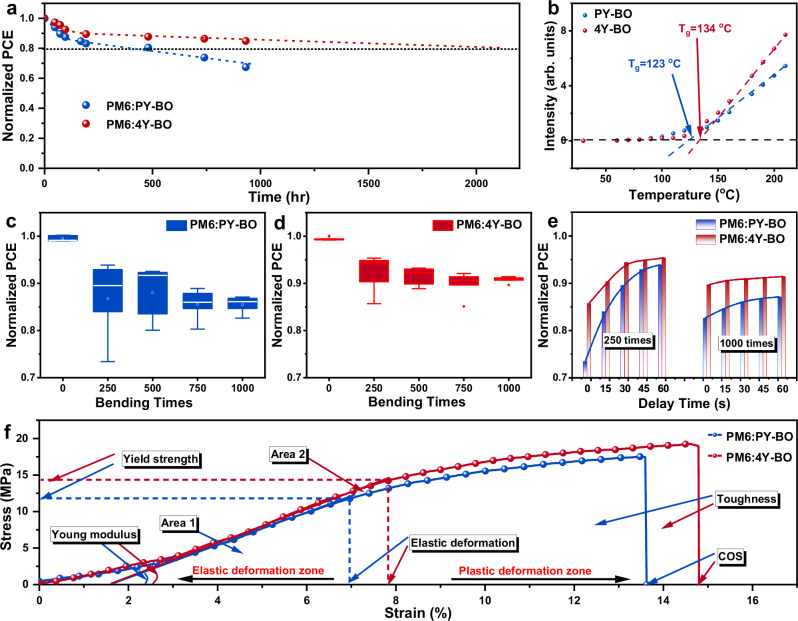
Thermal and mechanical stability characteristics of solar cells. **a** Thermal stability of PM6:PY-BO and PM6:4Y-BO based OSCs. **b** Deviation metric (DM_T_) plots of PY-BO and 4Y-BO neat films as a function of annealing temperature. **c**, **d** Bending stability of solar cells at a bending radius of 5 mm. **e** Performance rebound characteristics after 250 and 1000 bending cycles. **f** Stress-strain testing of PM6:PY-BO and PM6:4Y-BO blend films.

In the above studies, we have highlighted the high photovoltaic performance of tetramer acceptors surpassing the polymer acceptor and disclosed the critical differences from the perspectives of molecular aggregation, exciton/charge management, and heterojunction morphology. Besides of photovoltaic efficiency, device stability and mechanical flexibility are other pivotal parameters to evaluate solar cells, particularly for the all-PSCs. In the following part, we focus on the device stability, including thermal stability, photostability and mechanical stability. Figure 6a display the efficiency evolutions of PM6:PY-BO and PM6:4Y-BO based OSCs under continuous annealing at 80 ^o^C. Intriguingly, the PM6:4Y-BO based OSCs exhibited more robust thermal stability (*T*_80%_ lifetime of 2125 hr) compared with the devices based on PM6:PY-BO (*T*_80%_ lifetime of 512 hr). The improved device stability based on tetramer 4Y-BO should be attributed to the absence of low molecular-weight fractions, which can effectively suppress molecular creep and delay the morphology degradation. Glass transition temperatures (*T*_g_) of PY-BO and 4Y-BO films are measured based on deviation metric (DM_T_) plots of absorption profiles as a function of annealing temperature. As displayed in Fig. 6b, the *T*_g_s of PY-BO and 4Y-BO films are determined to be 123 ^o^C and 134 ^o^C, respectively, partly supporting the more preferred device stability based on 4Y-BO. It is worth noting that within the range of 75 ^o^C to *T*_g_, PY-BO film exhibits more significant state changes, which may be due to the aggregation variations caused by the peristaltic behavior of low molecular-weight fragments. With a uniform and high molecular weight, the tetramer 4Y-BO effectively inhibited molecular motion (below *T*_g_) and thus reduced the burn-in loss. Moreover, the photostability of PM6:PY-BO and PM6:4Y-BO based devices were performed and provided in Supplementary Fig. 37. After continuous illumination for 150 hrs under a solar simulator, it can be seen that the PM6:4Y-BO based OSC maintains 92.6% of its initial PCE, significantly higher than that of the PM6:PY-BO device (83.4%). The improvement in photostability should be correlated with the ordered molecular stacking of the PM6:4Y-BO blend, which enhances charge transport and reduces carrier recombination to retard material aging and degradation caused by energy accumulation. Meanwhile, stronger intermolecular interactions may help to stabilize the material structure and suppress the generation of free radicals^60,61^.

Bending stability of the PM6:PY-BO and PM6:4Y-BO based flexible OSCs were subsequently investigated, with results shown in Fig. 6c and d, respectively. Generally, the PCEs gradually decrease with increasing the bending cycles. After 1000 cycles of bending with a bending radius of 5 mm, the PM6:4Y-BO based flexible devices receives a slightly higher PCE preservation of 91% than that of PM6:PY-BO solar cells (87%). The improved mechanical flexibility of PM6:4Y-BO should be partly attributed to the greater intermolecular interactions in acceptor phases and the stronger interfacial interactions between the donor and acceptor phases (Supplementary Figs. 16 and 32). Of greater importance, we wondrously observed that the flexible devices exhibit significant performance rebound within one minute after bending. As indicated in Fig. 6e, the PM6:4Y-BO based flexible OSCs demonstrate a more linear efficiency rebound pattern compared to PM6:4Y-BO based devices, which should be correlated with the larger elastic modulus of the active layer. In order to gain an in-depth study of the mechanical properties of active layers, we measured the stress-strain curves of the PM6:PY-BO and PM6:4Y-BO blend films (Fig. 6f). The crack onset strains (COS) of PM6:PY-BO and PM6:4Y-BO based blend films are determined to be 13.57% and 14.80%, respectively. Owing to the generally high polymerization degrees of donor material, the longer elongation at break of PM6:4Y-BO blend film should be contributed by the absence of low molecular-weight fragments, which strengthens the stress-bearing capacity to reduce the tearing of the blend film. Before reaching fracture state, the film typically undergoes two processes of elastic deformation and plastic deformation, and the elastic deformation of PM6:PY-BO and PM6:4Y-BO based blend films are determined to be 5.35% and 6.22%, respectively. Our previous study suggested that elastic deformation is the most reliable to reflect bending stability of OSCs^17^. Elastic modulus refers to the speed at which film recovers to its original shape after removing external forces, determined by the ratio of yield strength to elastic deformation. Results show that the PM6:4Y-BO blend film presents a greater elastic modulus of 232 MPa than that of PM6:PY-BO (221 MPa), which can well explain the more favorable performance rebound process (Fig. 6e). Therefore, high-quality flexible OSCs require both high elastic deformation and elastic modulus. In addition, the modulus of resistance takes into account both elastic deformation and elastic modulus parameters, which can be used to describe the ability of films to store elastic potential energy during elastic deformation. The modulus of resistance of the PM6:PY-BO and PM6:4Y-BO based blend films are 0.32 MPa and 0.45 MPa, respectively, which further demonstrate the great promise of tetramer 4Y-BO over polymer PY-BO in the field of flexible photovoltaic wearables.

## Discussion

In order to solve the dilemma faced by polymer acceptors, in this study, we report a linear tetramer oligomer acceptor, namely 4Y-BO, with a considerable molecular weight of 7.36 kDa and partial polymer-like characteristics. Comprehensive comparisons between 4Y-BO and the counterpart polymer acceptor PY-BO suggest that the tetramer acceptor receives homogeneous crystallinity kinetics and, more notably molecular ordering, which enable preferable fibrous heterojunction morphology with decent and uniform crystallinity between the polymer donor and tetramer acceptor. Beneficial from the advantageous phase-separations, the PM6:4Y-BO based OSCs achieve more efficient exciton and charge properties, realizing a very competitive PCE up to 19.75% and greatly surpasses that of PM6:PY-BO (15.66%). Of greater importance, the PM6:4YBO based OSCs exhibited improved thermal stability (*T*_80%_ lifetime of 2125 hrs) after continuous heated at 80 ^o^C and photostability (*T*_92.6%_ lifetime of 150 hrs) after continuous illumination. Besides, the PM6:4Y-BO based flexible OSCs afford enhanced bending stability and efficiency rebound characteristics compared to those of PM6:PY-BO, which further confirms the tetramer acceptor over polymer acceptor on mechanical stability. Our study highlights the great potential of linear oligomer acceptors with considerable molecular weights for highly efficient and stable solar cells, which should be helpful to further proceed with the development of state-of-the-art organic photovoltaics.

## Methods

### Materials

Donor material PM6 and PDINN were purchased from Solarmer Materials Inc. PEDOT: PSS (Clevios AI 4083) was purchased from Heraeus, Germany. The intermediates of acceptors are purchased from Nanjing Zhiyan Technology Co., Ltd. Other reagents were purchased from Alfa Aesar, Sigma-Aldrich, et al., which were utilized directly unless stated otherwise. The synthesis and characterization details of PY-BO and 4Y-BO are provided in the Supporting Information.

### Molecular structure and property characterization Techniques

^1^H NMR and ^13^C NMR spectra were recorded on Bruker AVANCE III 600 MHz spectrometer at 298 K. The absorption spectra were recorded using a Hitachi U-4100 UV-Vis scanning spectrophotometer. were recorded using HORIBA Nanolog. Cyclic voltammetry (CV) measurements were performed on a CHI660D electrochemical workstation equipped with a three-electrode cell consisting of a platinum working electrode, a saturated calomel electrode (SCE) as reference electrode, and a platinum wire counter electrode. CV measurements were carried out in anhydrous acetonitrile containing 0.1 M *n*-Bu_4_NPF_6_ as a supporting electrolyte under an argon atmosphere at a scan rate of 100 mV s^−1^ assuming that the absolute energy level of Fc/Fc^+^ was − 4.80 eV. Thin films of three acceptors were deposited from CHCl_3_ solutions for the measurements of UV-vis absorption spectra. The molecular weight and polydispersity index (PDI) of the polymer acceptor were determined by high-temperature (150 ^o^C) gel permeation chromatography (GPC) using 1, 3, 5-trichlorobenzene as the eluent and polystyrene as the standard.

In situ absorptions were carried out from the dynamic spectrometer DU-300, with chloroform as the working solvent. Femtosecond transient spectroscopy (fs-TA) spectra were measured by Excipolar Spinon Optoelectronics with a pump wavelength of 780 nm, pump energy of 200 nJ, and time delay ranges of 10 ps-7.6 ns. Grazing incidence wide-angle X-ray scattering (GIWAXS) patterns were acquired from the Shanghai Synchrotron Radiation Facility at the beam BL6B1. Transmission electron microscopy (TEM) images were obtained by using a HITACHI H-7650 electron microscope with an acceleration voltage of 100 kV. Atomic force microscopy (AFM) images were obtained using Agilent 5400 scanning probe microscope in tapping mode with MikroMasch NSC-15 AFM tips. Kelvin probe force microscopy (KPFM) images were also obtained from the Agilent 5400 scanning probe microscope. Contact angles are measured by the contact angle measuring instrument CSCDIC-200S.

### Device fabrication and evaluations

All the solar cells were fabricated with a conventional device structure of ITO/PEDOT:PSS/active layer/PDINN/Ag. The patterned ITO glass (sheet resistance = 15 Ω/ square) was pre-cleaned in an ultrasonic bath of acetone and isopropyl alcohol and treated in an ultraviolet-ozone chamber (PREEN II-862) for 6 min. Then a thin layer (about 30 nm) of PEDOT:PSS was spin-coated onto the ITO glass at 4000 rpm and baked at 150 ^o^C for 15 min. PM6:PY-BO/PM6:4Y-BO based solutions (PM6 concentration: 7 mg/ml in chloroform) with 100 wt% 2-ethoxynaphthalene (2-EN) or 1 vol% 1-chloronaphthalene (CN) were stirred for 2 h at 50 ^o^C before spin-coating on the PEDOT:PSS layer. The spin-coating speeds are in the range of 2500–3500 rpm to modulate the film thickness. All films were subsequently annealed at 80 ^o^C for 5 min. The thickness of the active layer was about 110 ± 20 nm, measured using a Veeco Dektak 150 profilometer. After completing the active layer fabrication, then PDINN (in CH_3_OH, 1 mg/mL) was spin-coated at 3000 rpm to form the electron transfer layer. Finally, the Ag (60 nm) metal electrode was thermal evaporated under about 5 × 10^−4^ Pa, and the device area was 0.0936 cm^2^ defined by the shadow mask.

The current density–voltage (*J*–*V*) characteristics were recorded with a Keithley 2400 source measurement unit under simulated 100 mW cm^−^^2^ irradiation from a Newport solar simulator in high-purity nitrogen-filled glove box (H2O < 0.01 PPm; O2 < 0.01 PPm). The solar simulator is calibrated by a standard silicon cell (SRC-00178) before device testing. The standard silicon cell was last certified in September 2022. The dark current test is in an opaque box. The J-V curves were measured in forward scan mode (from − 0.2 V to 1.2 V) with a scan step length of 0.02 V and dwell time of 50 ms. The pre-sweep delay is 0.5 s. The external quantum efficiencies (EQEs) were analyzed using a certified Newport incident photon conversion efficiency (IPCE) measurement system. The hole mobility and electron mobility were measured by the space-charge-limited current (SCLC) method with a device configuration of ITO/PEDOT:PSS/active layer/MoO_3_/Al and ITO/ZnO/active layer/PDINN/Al structure, respectively.

### Theoretical calculations

The geometry optimization of the 4Y-BO and PY-BO were comprehensively optimized by employing the B3LYP function with 6–31 g(d,p) basis sets. The counterpoise correction (CP) method was used to account for the basis set superposition error (BSSE) when calculating the intermolecular interaction energy. The molecular electrostatic potential (ESP), averaged local ionization energy (ALIE), and electron and hole distributions analysis were implemented using Multiwfn 3.8, and outputs are visualized using VMD 1.9.3. The excitation properties calculation of the 4Y-BO and PY-BO were performed by TD-DFT method with CAM-B3LYP/6-311 g(d,p)/polarizable continuum model.

### Reporting summary

Further information on research design is available in the Nature Portfolio Reporting Summary linked to this article.

## Supplementary information

Supplementary Information

Solar Cells Reporting Summary

Transparent Peer Review file

## Source data

Source data

## Data Availability

All the data generated in this study are provided in the Article file, Supplementary Information file, and Source Data file. Source data are provided in this paper.
