# Supplementary material for: Polymer-like tetramer acceptor enables stable and 19.75% efficiency binary organic solar cells
Source: Nat Commun. 2025 Feb 20;16:1784. doi: 10.1038/s41467-025-57118-9 (PMC11840019; doi:10.1038/s41467-025-57118-9)
Supplement: Supplementary file 1 — Supplementary Information [file 41467_2025_57118_MOESM1_ESM.pdf]

Supplementary Information

Polymer-Like Tetramer Acceptor Enables Stable and 19.75% Efficiency Binary Organic Solar Cells

Jianxiao Wang[1,2,3], Cheng Sun[1,2,3], Yonghai Li[1,2,3]*, Fuzhen Bi[1,2,3], Huanxiang Jiang[4], Chunming Yang[5], Xichang Bao[1,2,3]*, Junhao Chu[1,2,3]

[1]Key Laboratory of Photoelectric Conversion and Utilization of Solar Energy, Qingdao Institute of Bioenergy and Bioprocess Technology, Chinese Academy of Sciences, Qingdao 266101, China

[2]Laboratory of Solar Energy, Shandong Energy Institute, Qingdao 266101, China

[3]Qingdao New Energy Shandong Laboratory, Qingdao 266101, China

[4]College of Textiles and Clothing, State Key Laboratory of Bio-fibers and Eco-textiles, Qingdao University, Qingdao 266071, China

[5]Shanghai Synchrotron Radiation Facility Shanghai Advanced Research Institute, Chinese Academy of Sciences, Shanghai 201204, China

*E-mails: baoxc@qibebt.ac.cn; liyh@qibebt.ac.cn

Supplementary Methods

Materials and Characterization Techniques

Donor material PM6 was purchased from Solarmer Materials Inc. The intermediates of acceptors are purchased from Nanjing Zhiyan Technology Co., Ltd. Other reagents were purchased from Alfa Aesar, Sigma-Aldrich, et al., which were utilized directly unless stated otherwise.

^{1}H NMR and ^{13}C NMR spectra were recorded on Bruker AVANCE III 600 MHz spectrometer at 298 K. The absorption spectra were recorded using a Hitachi U-4100 UV-Vis scanning spectrophotometer. Cyclic voltammetry (CV) measurements were performed on a CHI660D electrochemical workstation, equipped with a three-electrode cell consisting of a platinum working electrode, a saturated calomel electrode (SCE) as reference electrode and a platinum wire counter electrode. CV measurements were carried out in anhydrous acetonitrile containing 0.1 M n-Bu_4NPF_6 as a supporting electrolyte under an argon atmosphere at a scan rate of 100 mV s^{-1} assuming that the absolute energy level of Fc/Fc^+ was -4.80 eV. Thin films of three acceptors were deposited from $CHCl_3$ solutions for the measurements of UV-vis absorption spectra. The molecular weight and polydispersity index (PDI) of the polymer acceptor were determined by high-temperature (150 ^oC) gel permeation chromatography (GPC) using 1, 3, 5-trichlorobenzene as the eluent and polystyrene as the standard.

In-situ absorptions were carried out from the dynamic spectrometer DU-300, with chloroform as the working solvents. Femtosecond transient spectroscopy (fs-TA) spectra were measured Excipolar Spinon Optoelectronics with a pump wavelength of 780 nm, pump energy of 200 nJ, and time delay ranges of 10 ps-7.6 ns.

Grazing incidence wide-angle X-ray scattering (GIWAXS) patterns were acquired from Shanghai Synchrotron Radiation Facility at the beam BL6B1.

Transmission electron microscopy (TEM) images were obtained by using a HITACHI H-7650 electron microscope with an acceleration voltage of 100kV. Atomic force microscopy (AFM) images were obtained using Agilent 5400 scanning probe microscope in tapping mode with MikroMasch NSC-15 AFM tips. Kelvin probe force microscopy (KPFM) images were also obtained from Agilent 5400 scanning probe microscope.

Contact angles are measured by the contact angle measuring instrument CSCDIC-200S. Contact angle test: The two liquid method is used to measure the contact angles. The films were coated onto

the glass substrate. Subsequently, H_2O and CH_2I_2 droplets contact the surface of the film and quickly disengage. By using the video recording function of the instrument, the continuous image of the drop, contact and stability of the droplets on the surface of films can be obtained, so as to accurately judge the contact angle image of CH_2I_2 droplets.

The geometry optimization of the 4Y-BO and PY-BO were comprehensively optimized by employing the B3LYP function with 6-31g(d,p) basis sets. The counterpoise correction (CP) method was used to account for the basis set superposition error (BSSE) when calculating the intermolecular interaction energy. The molecular electrostatic potential (ESP), averaged local ionization energy (ALIE), and electron and hole distributions analysis were implemented using Multiwfn 3.8 [1, 2]and outputs are visualized using VMD 1.9.3.[3] The excitation properties calculation of the 4Y-BO and PY-BO were performed by TD-DFT method with CAM-B3LYP/6-311g(d,p)/polarizable continuum model.

Device Fabrication and Evaluations

All the solar cells were fabricated with a conventional device structure of ITO/PEDOT:PSS/active layer/PDINN/Ag. The patterned ITO glass (sheet resistance = 15 Ω/ square) was pre-cleaned in an ultrasonic bath of acetone and isopropyl alcohol and treated in an ultraviolet-ozone chamber (PREEN II-862) for 6 min. Then a thin layer (about 30 nm) of PEDOT:PSS was spin-coated onto the ITO glass at 4000 rpm and baked at 150 °C for 15 min. PM6:PY-BO/PM6:4Y-BO based solutions (PM6 concentration: 7 mg/ml in chloroform) with 100 wt% 2-ethoxynaphthalene (2-EN) or 1 vol% 1-chloronaphthalene (CN) were stirred for 2 hrs at 50 °C before spin-coating on the PEDOT:PSS layer. The spin-coating speeds are in the range of 2500 to 3500 rpm to modulate the film thickness. All films were subsequently annealed at 80 °C for 5 minutes. The thickness of the active layer was about 110±20 nm, measured using a Veeco Dektak 150 profilometer. After completing the active layer fabrication, then PDINN (in CH_3OH, 1mg/mL) was spin-coating at 3000 rpm to form the electron transfer layer. Finally, Ag (60 nm) metal electrode was thermal evaporated under about 5×10^{-4} Pa and the device area was 0.0936 cm^2 defined by shadow mask.

The current density–voltage (*J–V*) characteristics were recorded with a Keithley 2400 source measurement unit under simulated 100 mW cm^{-2} irradiation from a Newport solar simulator in high-purity nitrogen-filled glove box (H2O < 0.01 PPm; O2 < 0.01 PPm). The solar simulator is calibrated by standard silicon cell (SRC-00178) before device testing. The standard silicon cell was last certified

in September 2022. The dark current test is in an opaque box. The J-V curves were measured in forward scan mode (from -0.2 V to 1.2 V) with a scan step length of 0.02 V and dwell time of 50 ms. The pre sweep delay is 0.5 s. The external quantum efficiencies (EQEs) were analyzed using a certified Newport incident photon conversion efficiency (IPCE) measurement system. The hole mobility and electron mobility were measured by space-charge-limited current (SCLC) method with a device configuration of ITO/PEDOT:PSS/active layer/MoO_3/Al and ITO/ZnO/active layer/PDINN/Al structure, respectively. The SCLC is described by the Mott–Gurney law:

$J = 9\varepsilon\mu V^2/(8L^3)$, where ε represents the dielectric constant of the metal, and μ is the carrier mobility, V is the voltage drop across the device and L is the thickness of the active layer.

The thermal stability was performed under 80 ºC in a nitrogen filled glove box, which possesses oxygen content below 0.1 ppm, and water content below 0.1 ppm. The device was placed under dark to exclude the light factor, and the light was only turned on when measuring the *J-V* plots. Every data point was averaged from at least five independent devices. The photostability was performed under continuous illumination in a solar simulator with light intensity of 100 mW cm^{-2} in nitrogen filled glove box, which possesses oxygen content below 0.1 ppm, and water content below 0.1 ppm.

Materials Synthesis

Synthesis of compound 2

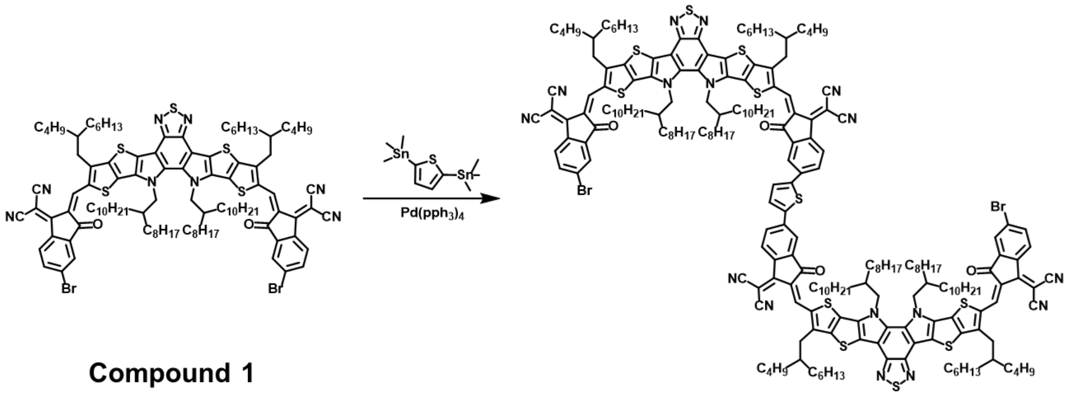

Compound 1 **Compound 2**

Compound **1** (500 mg, 0.263 mmol), 2,5-bis(trimethylstannyl)thiophene (27 mg, 0.066mmol), and $Pd(PPh_3)_4$ (3 mg, 0.0026 mmol) were combined in a 100 mL two-necked flask. Anhydrous toluene (30 mL) was added under the argon atmosphere. The mixture was reacted for 12 h at 100 °C. After removing residual solvents at low pressure (< 300 mbar) by a rotary evaporator, the product was purified by silica-gel column chromatography using hexane/dichloromethane (1:1) as eluent to give compound **2** as black solid (160 mg, 65%). ^{1}H NMR (600 MHz, Chloroform-*d*) δ 9.13 (d, *J* = 31.8 Hz, 4H), 8.76 (d, *J* = 8.1 Hz, 2H), 8.56 (d, *J* = 8.2 Hz, 2H), 8.11 (s, 2H), 8.06 (d, *J* = 63.2 Hz, 2H), 7.92 – 7.74 (m, 4H), 7.65 (s, 2H), 4.84 (t, *J* = 7.8 Hz, 8H), 3.25 – 3.02 (m, 8H), 2.21 (dd, *J* = 12.8, 6.6 Hz, 4H), 2.17 – 1.99 (m, 4H), 1.45 – 0.96 (m, 192H), 0.92 – 0.76 (m, 48H).

Synthesis of compound 4

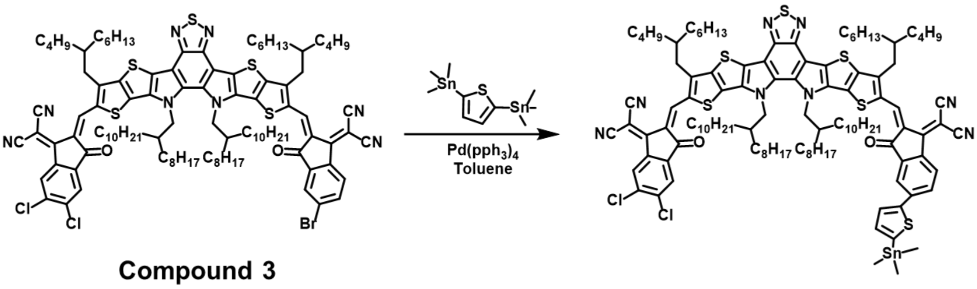

Compound 3 **Compound 4**

Compound **3** (500 mg, 0.264 mmol), 2,5-bis(trimethylstannyl)thiophene (1.08 g, 2.64 mmol), and $Pd(PPh_3)_4$ (5 mg, 0.0043 mmol) were combined in a 100 mL two-necked flask. Anhydrous toluene (50 mL) was added under the argon atmosphere. The mixture was reacted for 3 h at 70 °C. The crude product was precipitated in methanol and washed with methanol for three times, Compound **4** was used for next step without further purification.

Synthesis of compound 4Y-BO

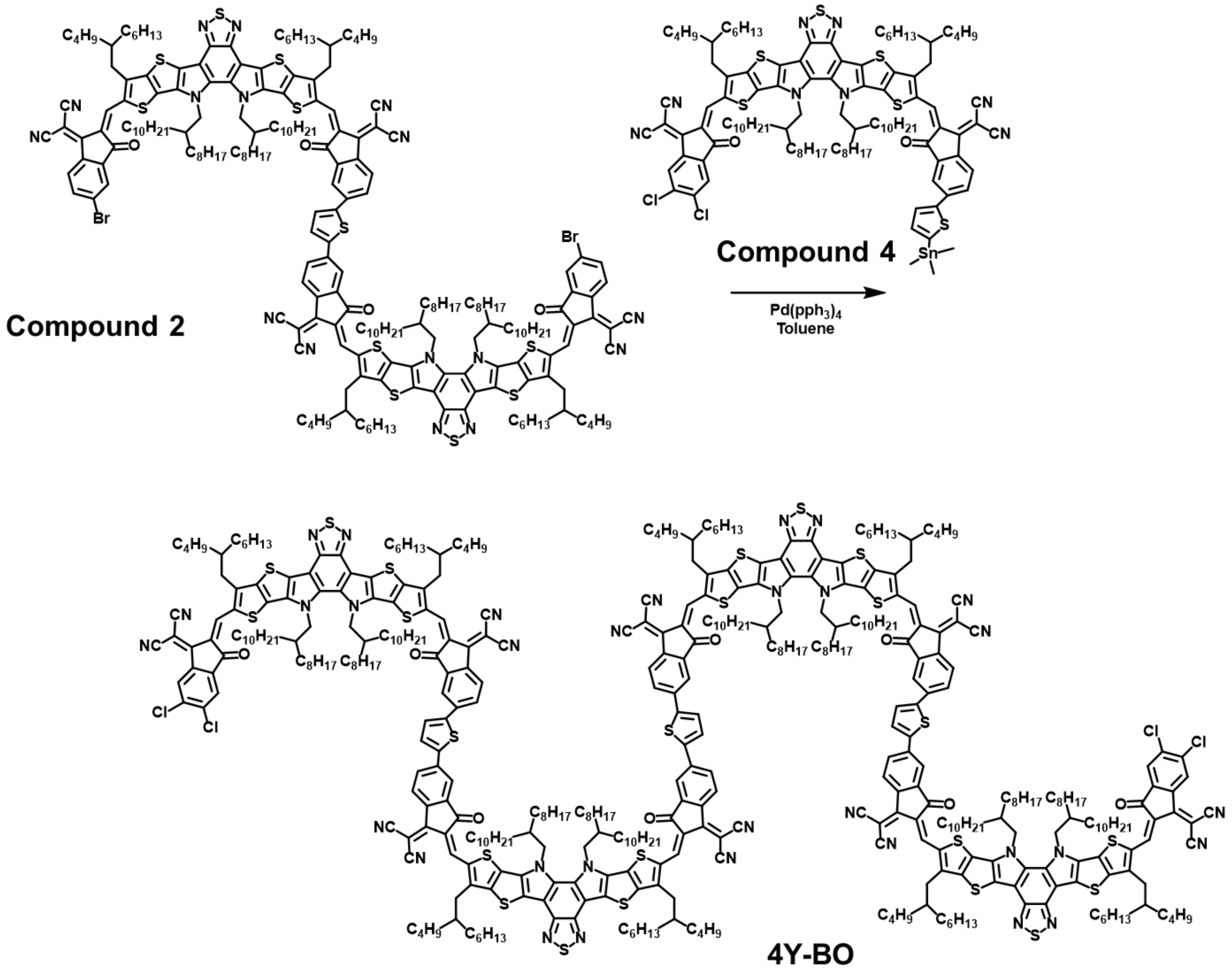

Compound **2** (100 mg, 0.027 mmol), Compound **4** (165 mg, 0.08 mmol), and $Pd(PPh_3)_4$ (5mg, 0.0043 mmol) were combined in a 100 mL two-necked flask. Anhydrous toluene (50 mL) was added under the argon atmosphere. The mixture was reacted for 12 h at 110 °C. After removing residual solvents at low pressure (< 300 mbar) by a rotary evaporator, the product was purified by column chromatography using hexane/ chloroform (1:2) as eluent to give 4Y-BO as black solid (159 mg, 80%). ^{1}H NMR (400 MHz, Chloroform-d) δ 9.34 – 8.72 (m, 16H), 8.51 – 7.54 (m, 16H), 7.54 – 7.34 (m, 4H), 5.26 – 4.58 (s, 16H), 3.40 – 2.11 (m, 32H), 1.49 – 1.04 (m, 384H), 0.93 – 0.65(m, 96H). MS (MALDI-TOF) m/z: $[M + H]^+$ calculated for $C_{444}H_{564}Cl_4N_{32}O_8S_{23}$, 7347.70, found: 7348.71.

Synthesis of compound PY-BO

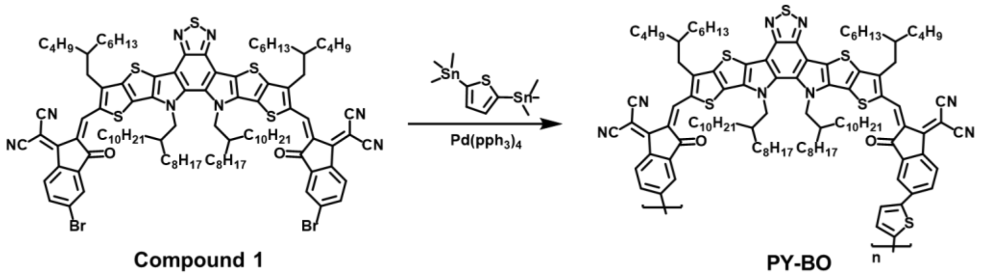

Compound **1** (200 mg, 0.105 mmol), 2,5-bis(trimethylstannyl)thiophene (43 mg, 0.105 mmol), and $Pd(pph_3)_4$ (3.6 mg, 0.0032 mmol) were combined in a 10 mL two-necked flask. Anhydrous toluene (6 mL) was added under the argon atmosphere. The mixture was reacted 24 h at 110 °C, after being cooled down, the reactant mixture was poured into methanol. The precipitate was filtered and Soxhlet extracted with methanol, hexane, and chloroform sequentially. The ingredient extracted from chloroform was concentrated, precipitated into 200 mL methanol, filtered and dried under vacuum to give the dark solid. (130 mg, 63%). Mn=9.96 kDa, Mw=18.7 kDa, PDI=1.88.

Supplementary Figures

MW Averages

Peak No	Mp	Mn	Mw	Mz	Mz+1	Mv	PD
1	19091	9962	18712	29599	41167	17131	1.87834

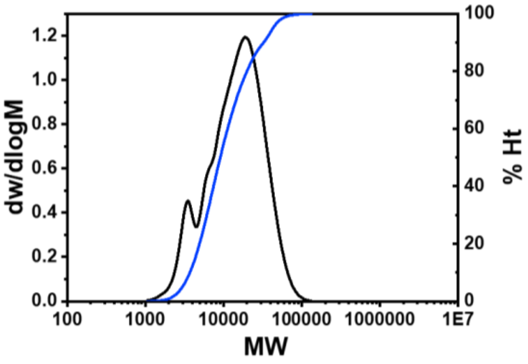

Supplementary Fig. 1 | GPC diagram of the polymer acceptor PY-BO.

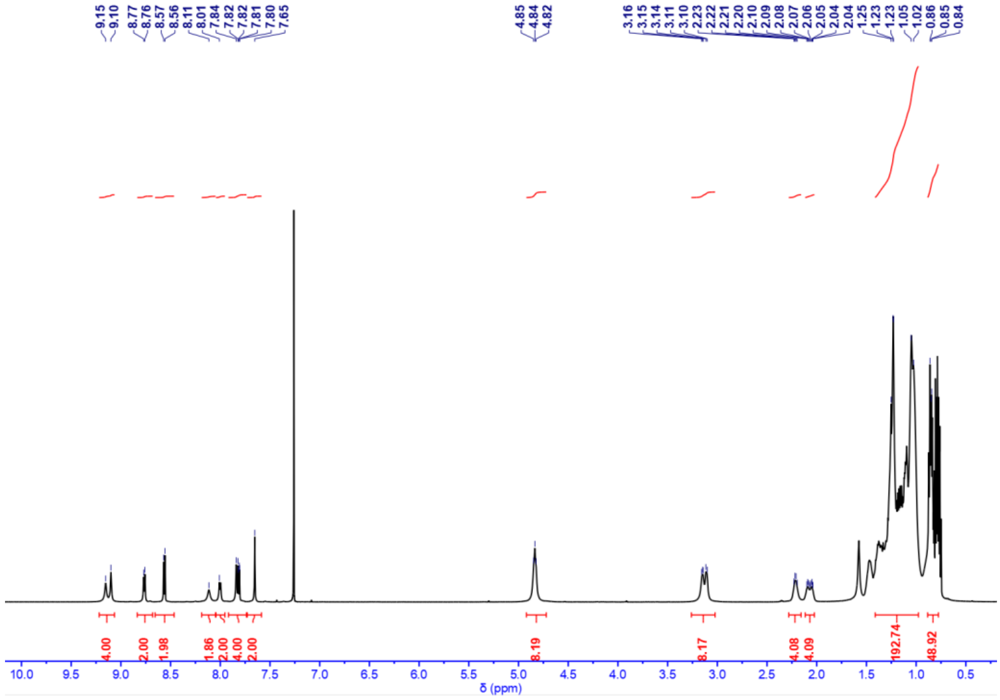

Supplementary Fig. 2 | ^{1}H NMR spectrum of compound **2**.

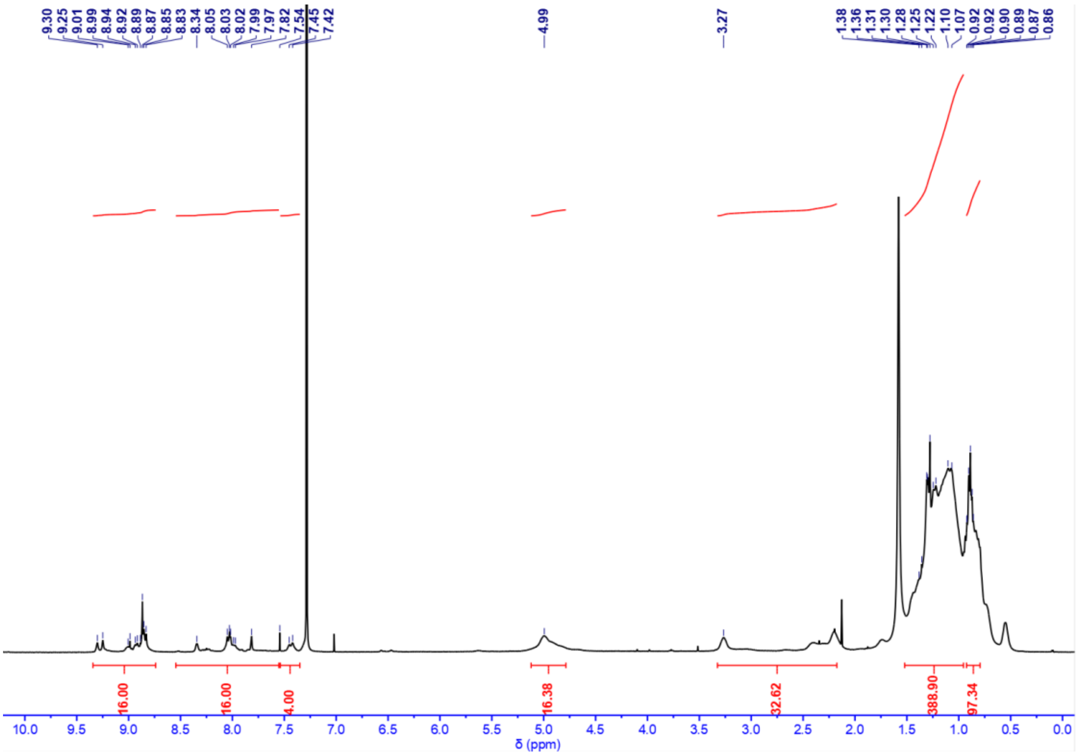

Supplementary Fig. 3 | ^{1}H NMR spectrum of tetramer acceptor 4Y-BO.

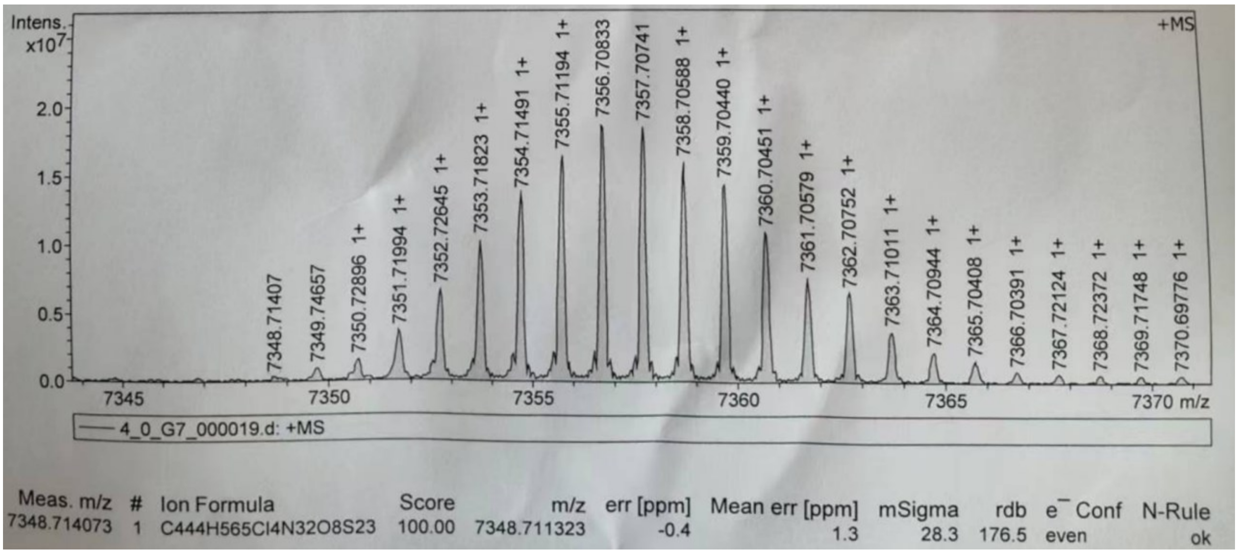

Supplementary Fig. 4 | MALDI-TOF spectrum of 4Y-BO.

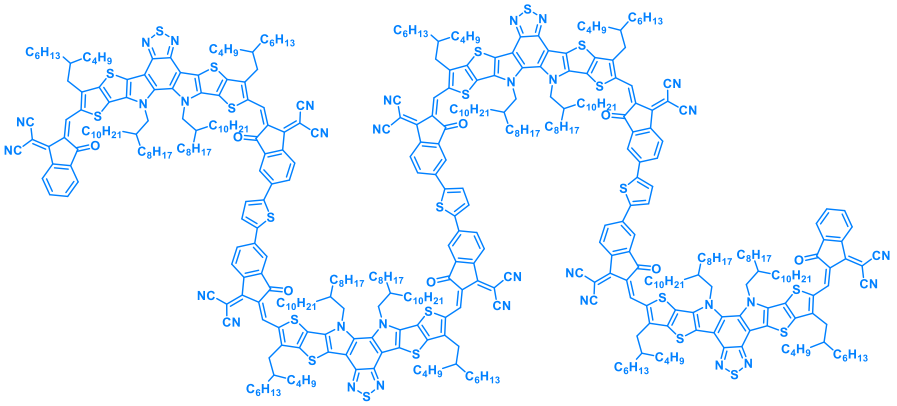

Supplementary Fig. 5 | Chemical structure of the tetramer fragment of PY-BO employed for simulation study.

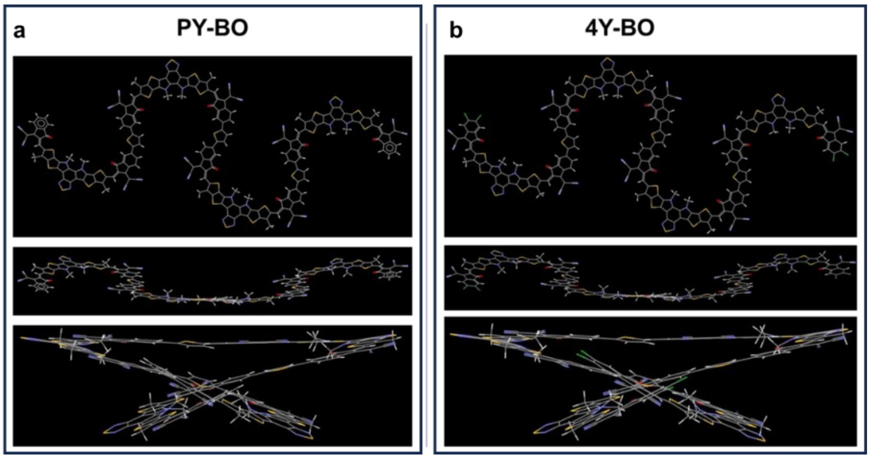

Supplementary Fig. 6 | The optimized molecular conformations of (a) PY-BO (four repeated units) and (b) 4Y-BO from different views.

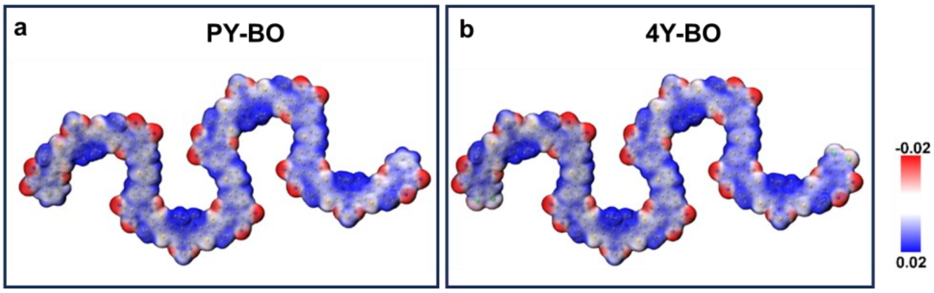

Supplementary Fig. 7 | ESP maps of the optimized molecular conformations of (a) PY-BO (four repeated units) and (b) 4Y-BO.

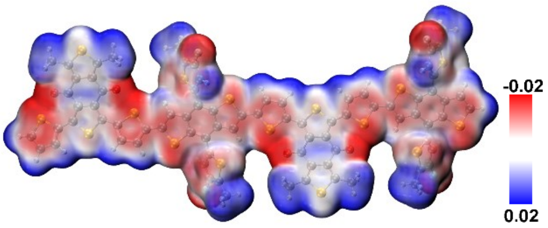

Supplementary Fig. 8 | ESP map of the optimized molecular conformation PM6 (two repeated units).

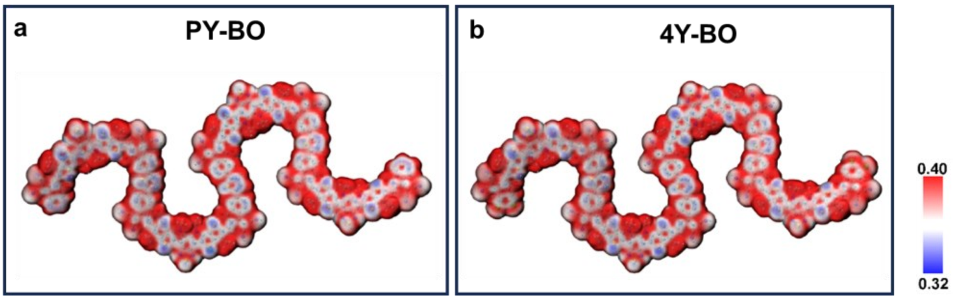

Supplementary Fig. 9 | ALIE maps of the optimized molecular conformations of (a) PY-BO (four repeated units) and (b) 4Y-BO.

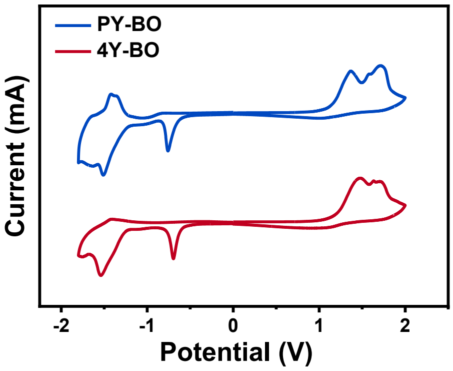

Supplementary Fig. 10 | The CV plots of PY-BO and 4Y-BO deposited on glassy carbon electrodes in 0.1 M Bu_4NPF_6-CH_3CN at a scan rate of 100 mV s^{-1}.

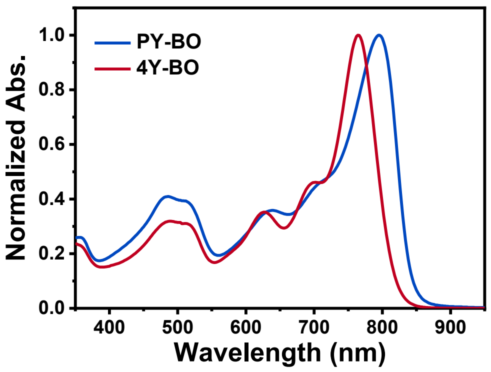

Supplementary Fig. 11 | Uv-vis absorption spectra of PY-BO and 4Y-BO in dilute chloroform solutions.

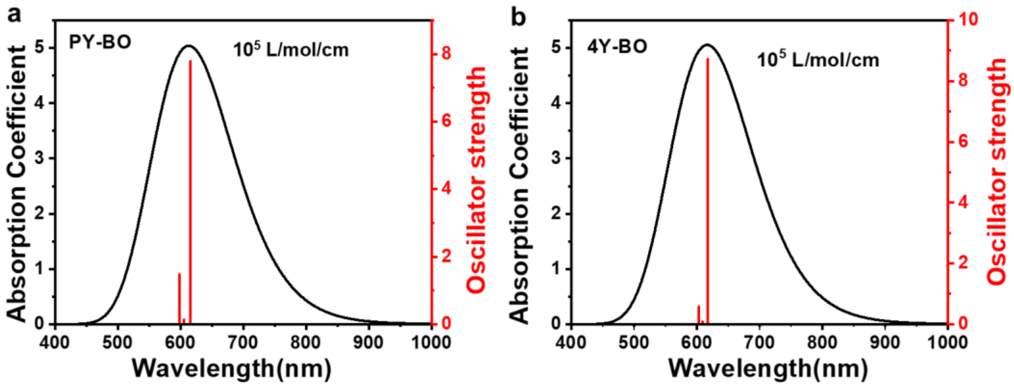

Supplementary Fig. 12 | Simulated absorption spectra of (a) PY-BO and (b) 4Y-BO in dibutylether solution (ε=3.0).

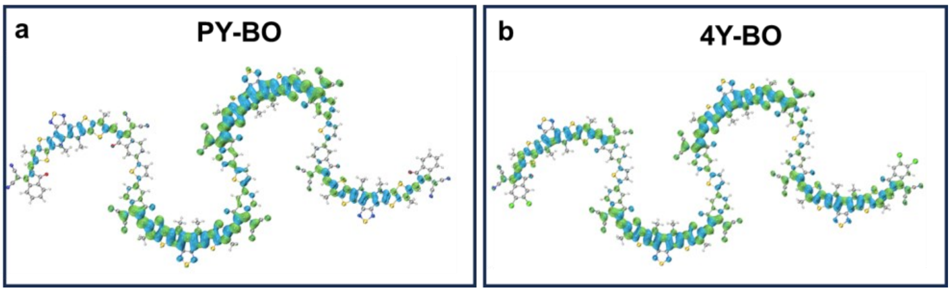

Supplementary Fig. 13 | Electron and hole distributions of S0→S1 for the (a) PY-BO and (b) 4Y-BO level. The green and blue contours correspond to the distribution of electron and hole, respectively.

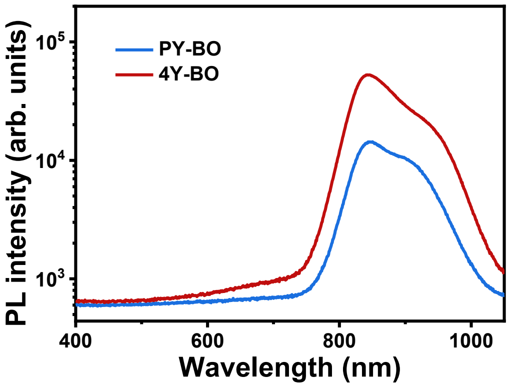

Supplementary Fig. 14 | PL spectra of PY-BO and 4Y-BO neat films.

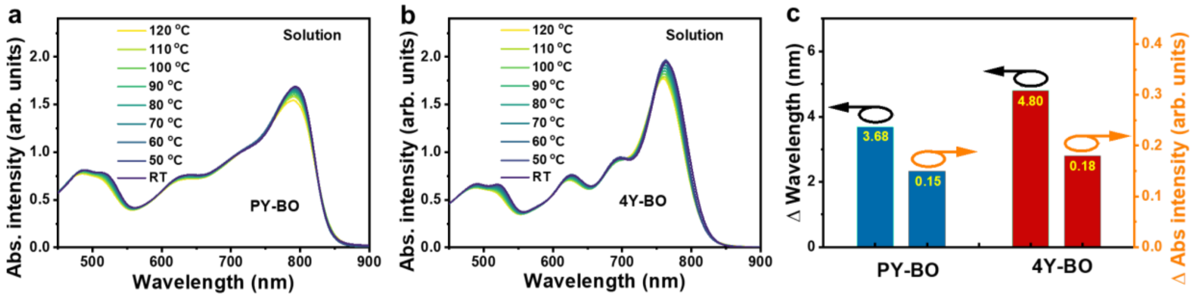

Supplementary Fig. 15 | Absorption spectra of (a) PY-BO and (b) 4Y-BO in CB solutions at different temperatures. (c) the variations of the intensity and position of the maximum absorption peaks.

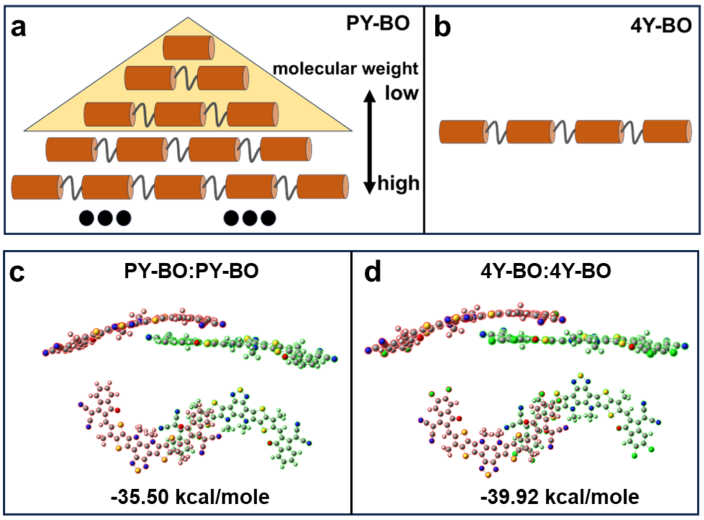

Supplementary Fig. 16 | Schematic diagram of molecular distribution of (a) PY-BO and (b) 4Y-BO. The optimized intermolecular packing diagrams of (c) PY-BO:PY-BO and (d) 4Y-BO:4Y-BO by density functional theory simulation. Single Y unit of 4Y-BO or PY-BO (with Cl or H end groups) employed for simplification.

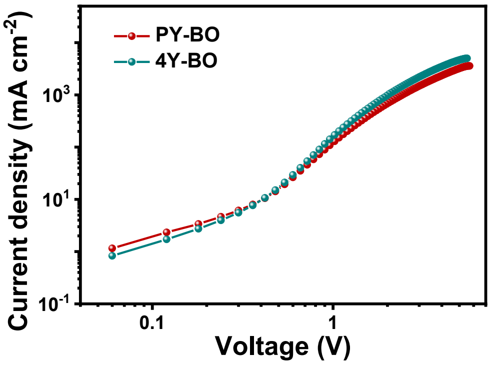

Supplementary Fig. 17 | The SCLC curves of PY-BO and 4Y-BO based hole-only and electron-only devices.

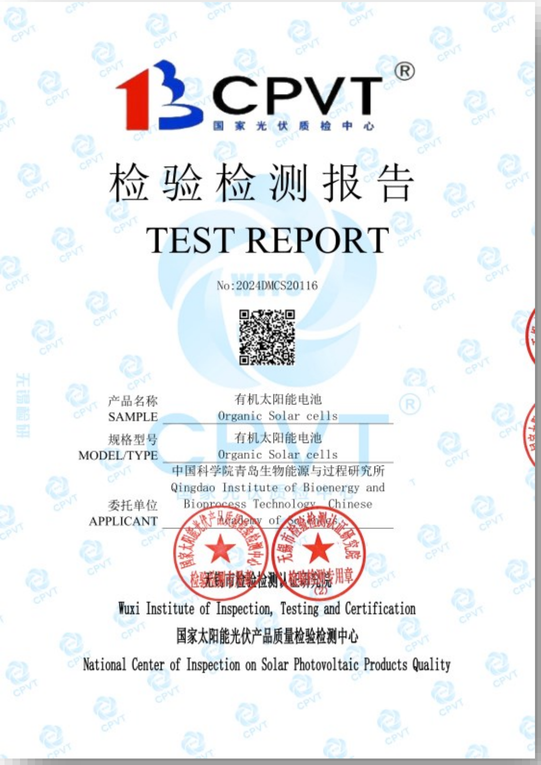

CPVT®
国 家 光 伏 质 检 中 心

检验检测报告

TEST REPORT

No:2024DMCS20116

产品名称 SAMPLE	有机太阳能电池 Organic Solar cells
规格型号 MODEL/TYPE	有机太阳能电池 Organic Solar cells
委托单位 APPLICANT	中国科学院青岛生物能源与过程研究所 Qingdao Institute of Bioenergy and Bioprocess Technology, Chinese Academy of Sciences

Wuxi Institute of Inspection, Testing and Certification

国家太阳能光伏产品质量检验检测中心

National Center of Inspection on Solar Photovoltaic Products Quality

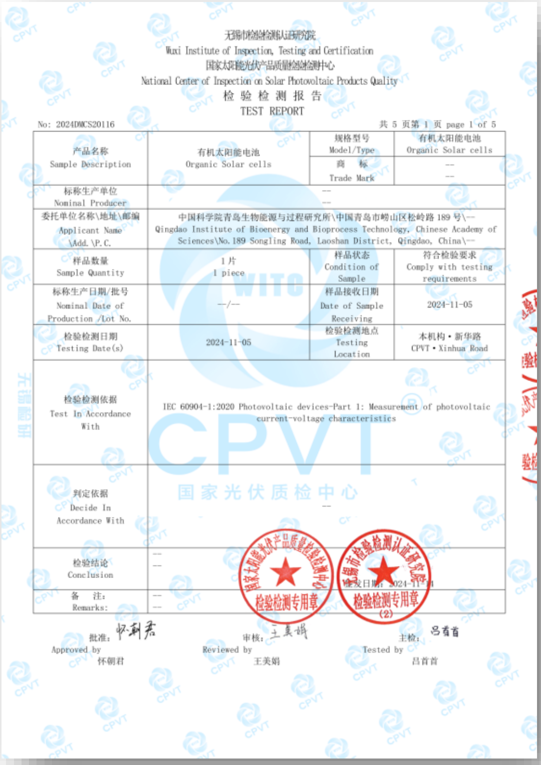

无锡市检验检测认证研究院

Wuxi Institute of Inspection, Testing and Certification

国家太阳能光伏产品质量检验检测中心

National Center of Inspection on Solar Photovoltaic Products Quality

检 验 检 测 报 告

TEST REPORT

No: 2024DMCS20116 共 5 页第 1 页 page 1 of 5

产品名称 Sample Description	有机太阳能电池 Organic Solar cells	规格型号 Model/Type	有机太阳能电池 Organic Solar cells
		商 标 Trade Mark	-- --
标称生产单位 Nominal Producer	-- --		
委托单位名称\地址\邮编 Applicant Name \Add. \P.C.	中国科学院青岛生物能源与过程研究所\中国青岛市崂山区松岭路 189 号\-- Qingdao Institute of Bioenergy and Bioprocess Technology, Chinese Academy of Sciences\No.189 Songling Road, Laoshan District, Qingdao, China\--		
样品数量 Sample Quantity	1 片 1 piece	样品状态 Condition of Sample	符合检验要求 Comply with testing requirements
标称生产日期/批号 Nominal Date of Production /Lot No.	--/--	样品接收日期 Date of Sample Receiving	2024-11-05
检验检测日期 Testing Date(s)	2024-11-05	检验检测地点 Testing Location	本机构•新华路 CPVT•Xinhua Road
检验检测依据 Test In Accordance With	IEC 60904-1:2020 Photovoltaic devices-Part 1: Measurement of photovoltaic current-voltage characteristics		
判定依据 Decide In Accordance With	--		
检验结论 Conclusion	-- --		
备 注: Remarks:	-- --		

签发日期: 2024-11-14

批准: Approved by 怀朝君 审核: Reviewed by 王美娟 主检: Tested by 吕首首

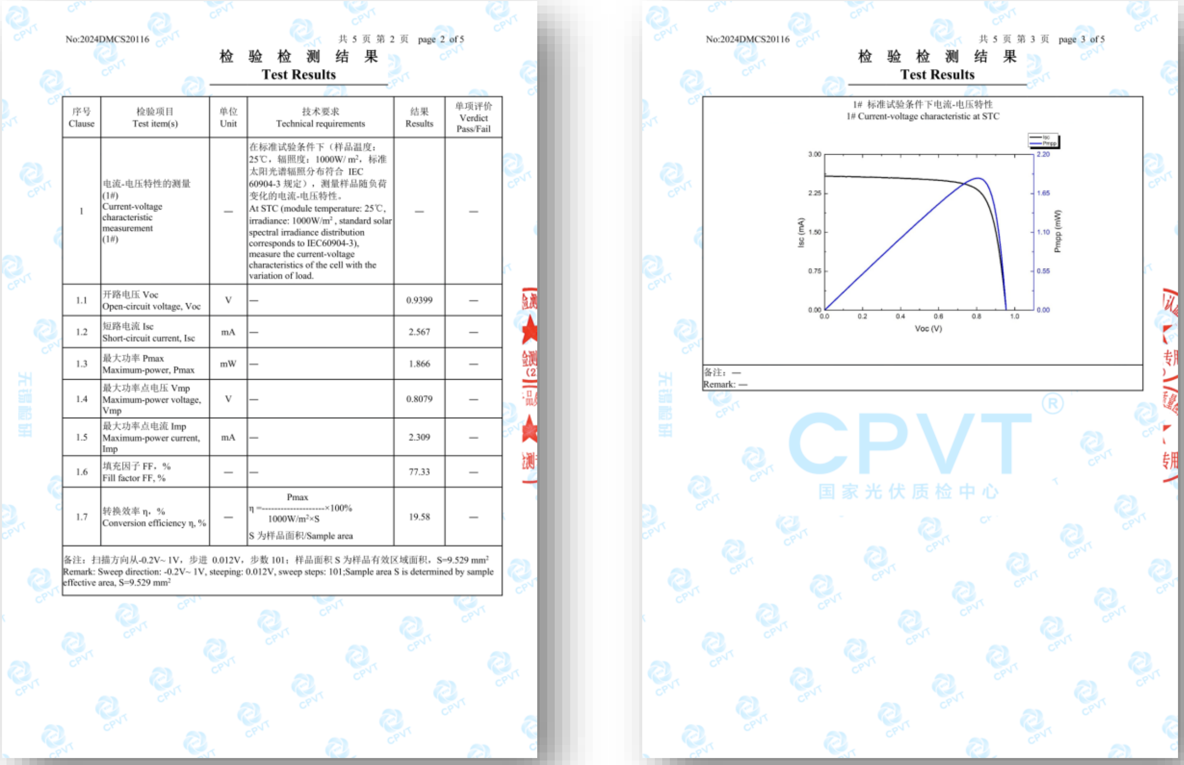
No:2024DMCS20116 共 5 页 第 2 页 page 2 of 5

检 验 检 测 结 果

Test Results

序号 Clause	检验项目 Test item(s)	单位 Unit	技术要求 Technical requirements	结果 Results	单项评价 Verdict Pass/Fail
1	电流-电压特性的测量 (1#) Current-voltage characteristic measurement (1#)	—	在标准试验条件下（样品温度：25℃，辐照度：1000W/ m²，标准太阳光谱辐照分布符合 IEC 60904-3 规定），测量样品随负荷变化的电流-电压特性。 At STC (module temperature: 25℃, irradiance: 1000W/m², standard solar spectral irradiance distribution corresponds to IEC60904-3), measure the current-voltage characteristics of the cell with the variation of load.	—	—
1.1	开路电压 Voc Open-circuit voltage, Voc	V	—	0.9399	—
1.2	短路电流 Isc Short-circuit current, Isc	mA	—	2.567	—
1.3	最大功率 Pmax Maximum-power, Pmax	mW	—	1.866	—
1.4	最大功率点电压 Vmp Maximum-power voltage, Vmp	V	—	0.8079	—
1.5	最大功率点电流 Imp Maximum-power current, Imp	mA	—	2.309	—
1.6	填充因子 FF，% Fill factor FF, %	—	—	77.33	—
1.7	转换效率 η，% Conversion efficiency η, %	—	$\eta = \frac{Pmax}{1000W/m^2 \times S} \times 100\%$ S 为样品面积/Sample area	19.58	—

备注：扫描方向从-0.2V~ 1V，步进 0.012V，步数 101；样品面积 S 为样品有效区域面积，S=9.529 mm²
Remark: Sweep direction: -0.2V~ 1V, steeping: 0.012V, sweep steps: 101;Sample area S is determined by sample effective area, S=9.529 mm²

No:2024DMCS20116 共 5 页 第 3 页 page 3 of 5

检 验 检 测 结 果

Test Results

1# 标准试验条件下电流-电压特性

1# Current-voltage characteristic at STC

备注：—
Remark: —

CPVT®
国家光伏质检中心

Supplementary Fig. 18 | The certification report from CPVT, China, as for PM6:4Y-BO device.

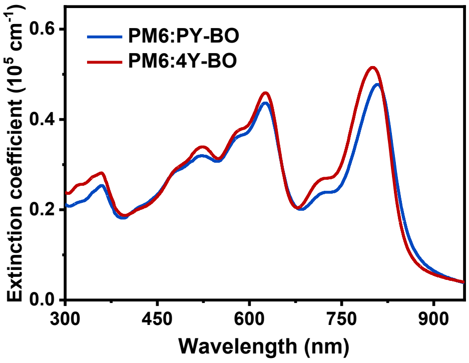

Supplementary Fig. 19 | The extinction coefficient spectra of PM6:PY-BO and PM6:4Y-BO neat blend films.

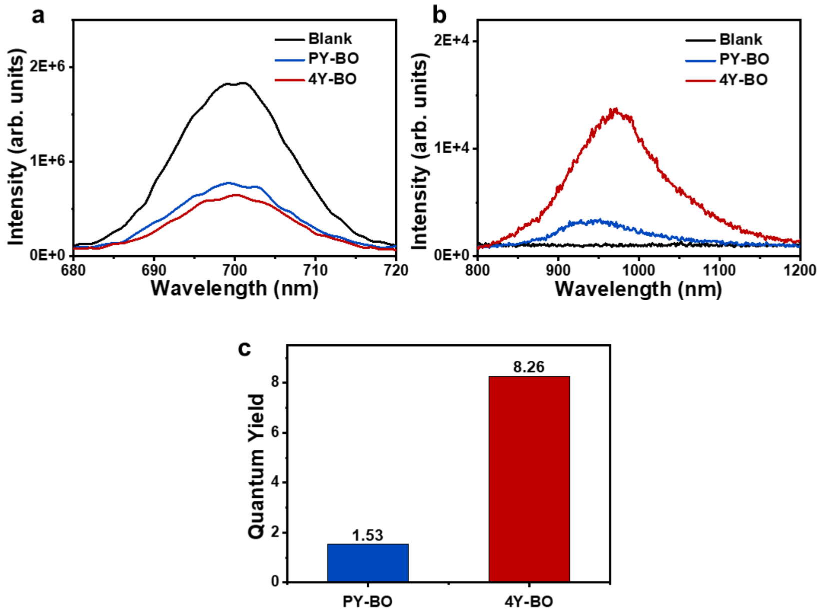

Supplementary Fig. 20 | a-b Excitation and emission spectra of blank sample, PY-BO and 4Y-BO, **c** Quantum Yield of PY-BO and 4Y-BO.

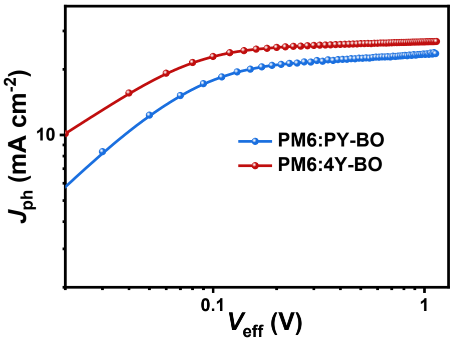

Supplementary Fig. 21 | The J_{ph} versus V_{eff} plots of the two optimal solar cells.

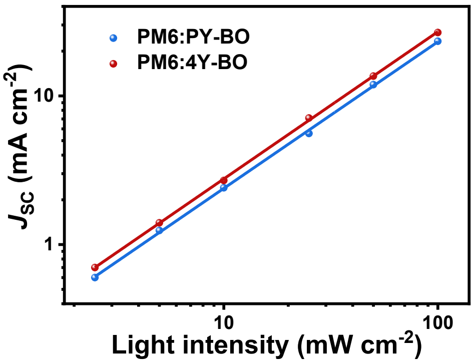

Supplementary Fig. 22 | The J_{SC} versus light intensity plots of the two optimal solar cells.

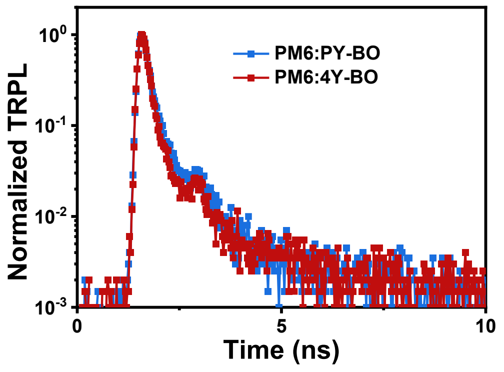

Supplementary Fig. 23 | The TRPL spectra of PM6:PY-BO and PM6:4Y-BO blend films.

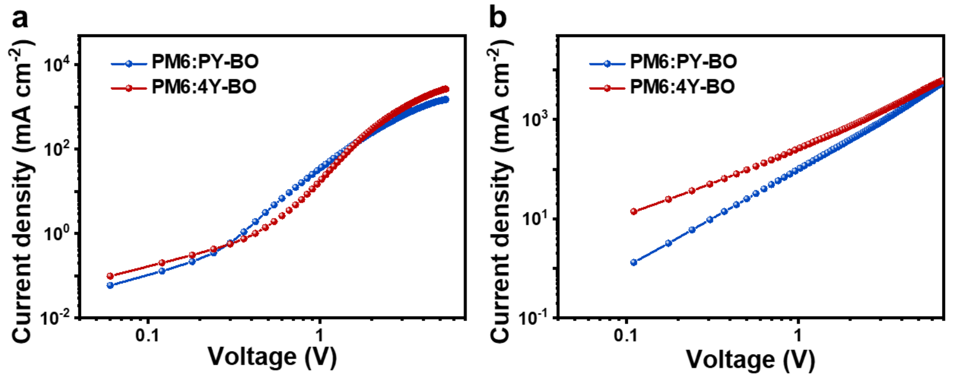

Supplementary Fig. 24 | The SCLC curves of PM6:PY-BO and PM6:4Y-BO based (a) hole-only and (b) electron-only devices.

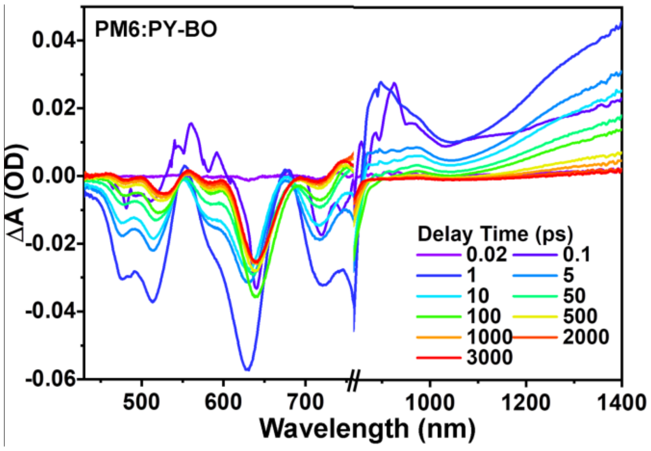

Supplementary Fig. 25 | TA spectra of PM6:PY-BO blend film with indicated decay times.

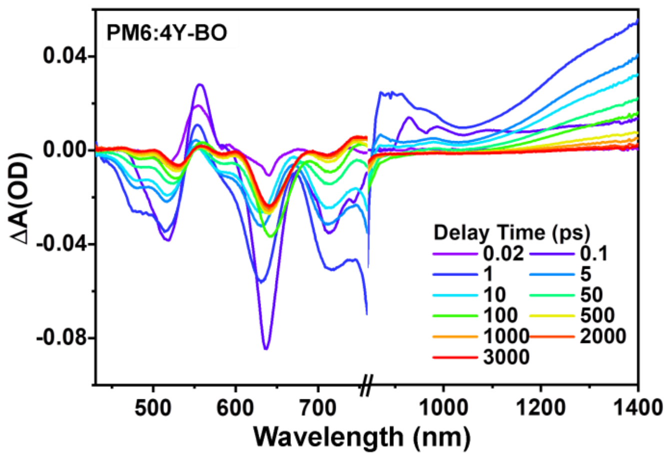

Supplementary Fig. 26 | TA spectra of PM6:4Y-BO blend film with indicated decay times.

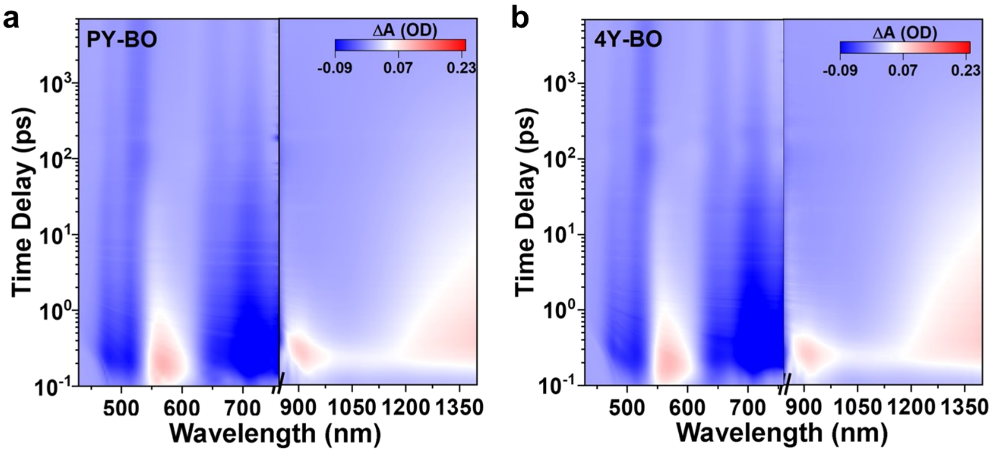

Supplementary Fig. 27 | 2D fs-TA patterns of (a) PY-BO and (b) 4Y-BO neat films pumped at 780 nm.

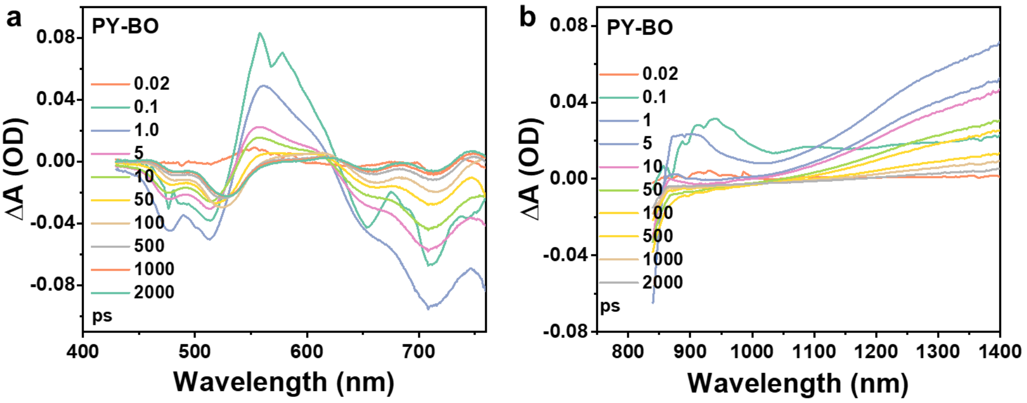

Supplementary Fig. 28 | TA spectra of PY-BO neat film with indicated decay times at (a) visible and (b) near infrared wavelength rang.

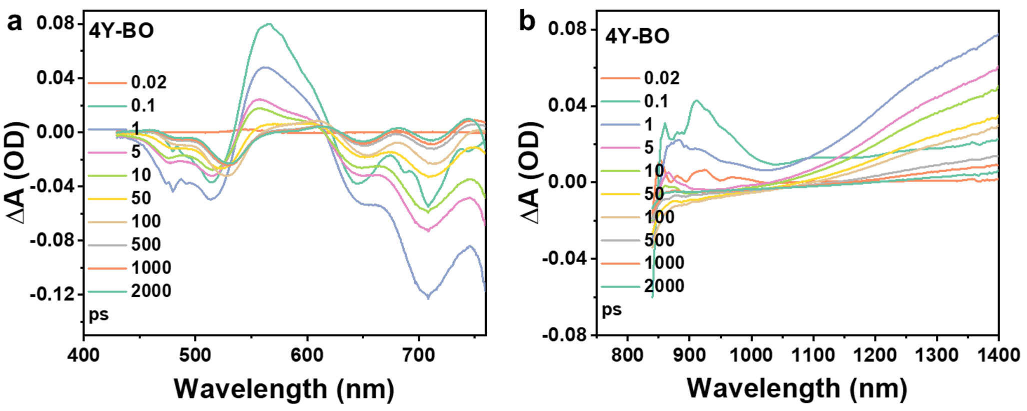

Supplementary Fig. 29 | TA spectra of 4Y-BO neat film with indicated decay times at (a) visible and (b) near infrared wavelength rang.

.

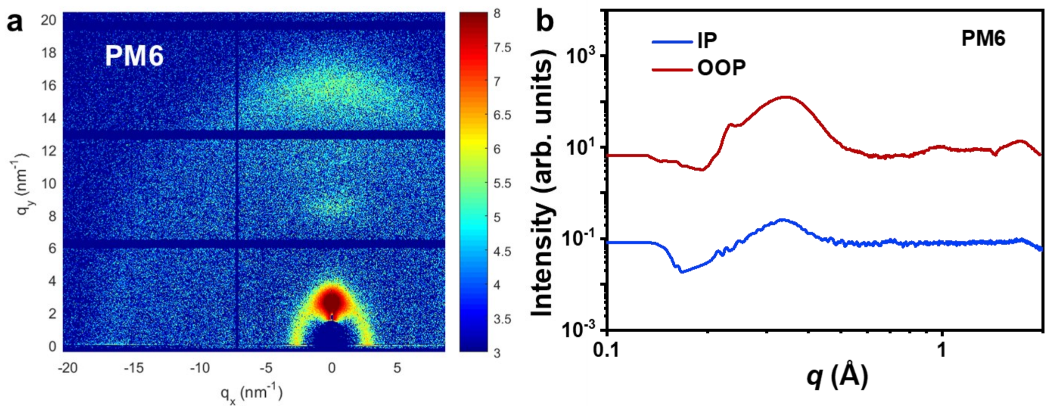

Supplementary Fig. 30 | (a) 2D-GIWAXS pattern and (b) line-cut profiles of neat PM6.

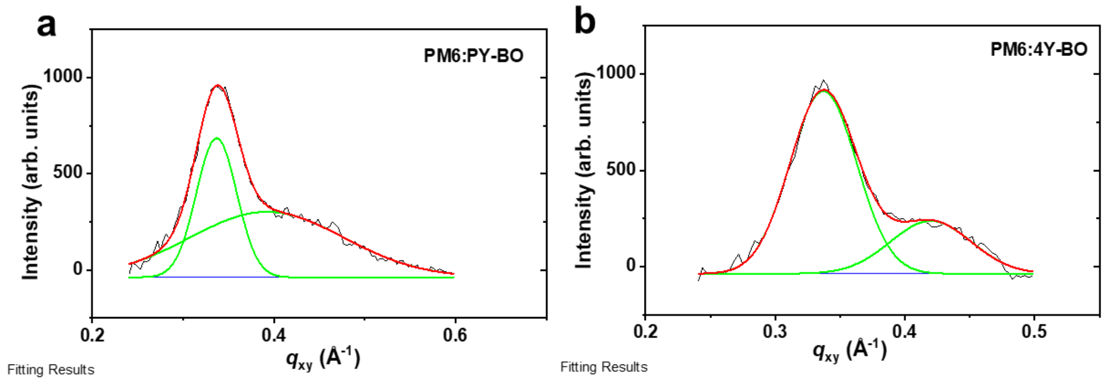

Supplementary Fig. 31 | The resolved (100) diffractions along IP direction based on the GIWAXS studies of the (a) PM6:PY-BO and (b) PM6:4Y-BO blend films.

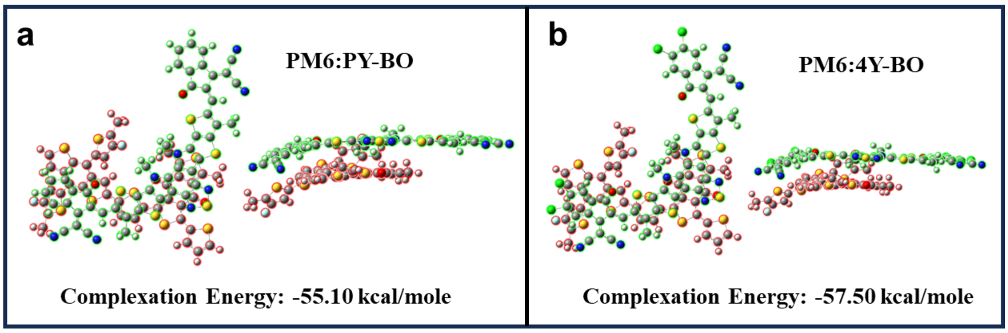

Supplementary Fig. 32 | Optimized intermolecular packing diagrams by density functional theory simulation at the lowest energy steady-state of (a) PM6:PY-BO and (b) PM6:4Y-BO molecular pairs.

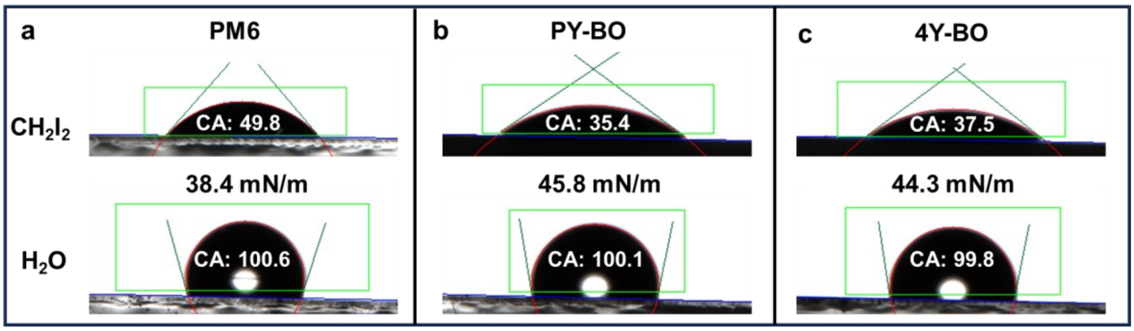

Supplementary Fig. 33 | Contact angles of water and diiodomethane droplets of (a) PM6, (b) PY-BO and (c) 4Y-BO neat films.

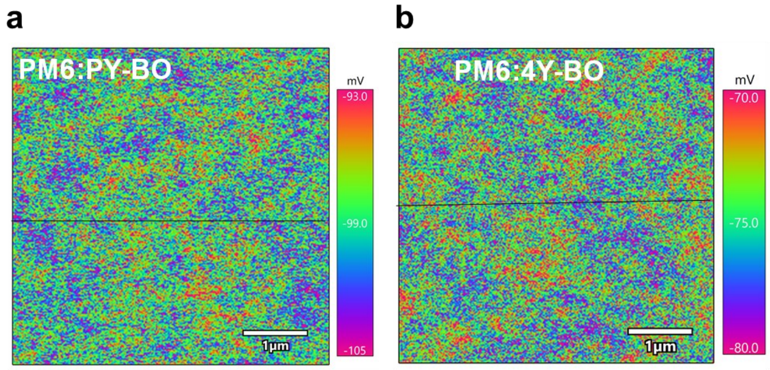

Supplementary Fig. 34 | KPFM images of (a) PM6:PY-BO and (b) PM6:4Y-BO blend films.

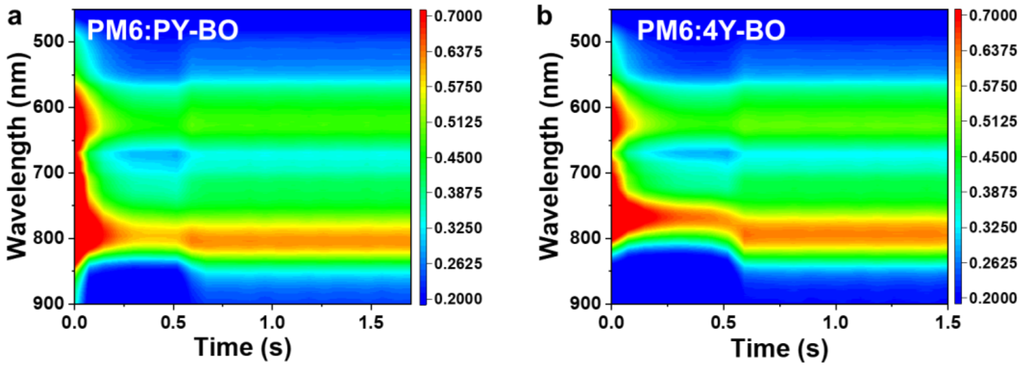

Supplementary Fig. 35 | 2D in-situ absorption patterns of (a) PM6:PY-BO and (b) PM6:4Y-BO blend systems casted from chloroform solutions.

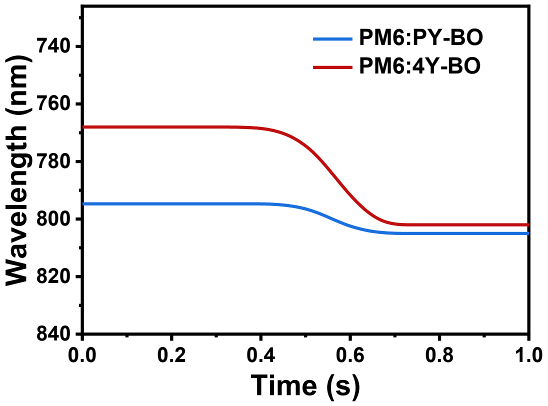

Supplementary Fig. 36 | Maximum peak variations of PM6:PY-BO and PM6:4Y-BO blend systems of *in-situ* absorptions.

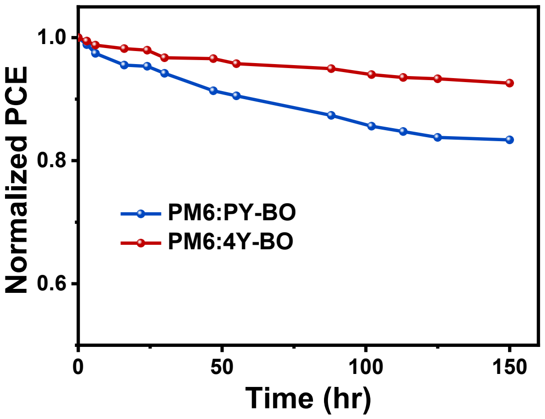

Supplementary Fig. 37 | The photostability of PM6:PY-BO and PM6:4Y-BO based devices.

Supplementary Tables

Supplementary Table 1 | The frontier molecular orbitals distribution for PY-BO and 4Y-BO.

	PY-BO	4Y-BO
L+3	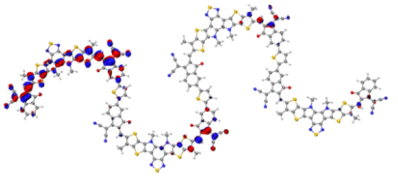 -3.57 eV	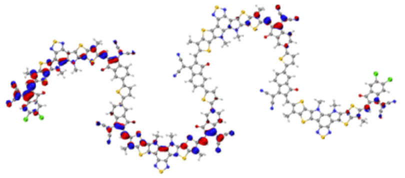 -3.63 eV
L+2	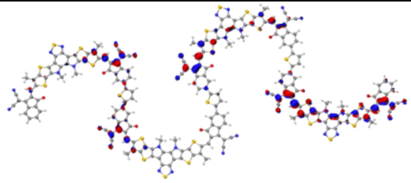 -3.64 eV	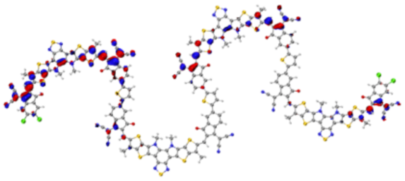 -3.68 eV
L+1	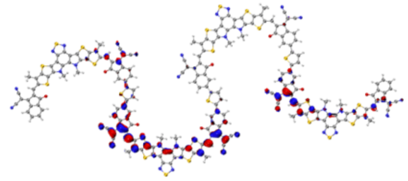 -3.69 eV	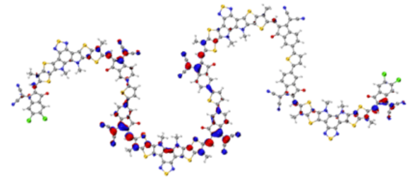 -3.72 eV
L	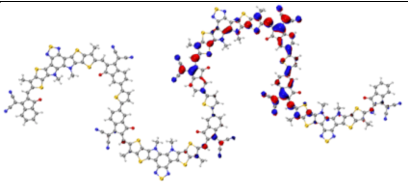 -3.74 eV	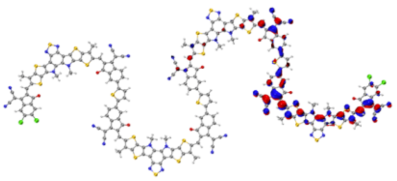 -3.77 eV
H	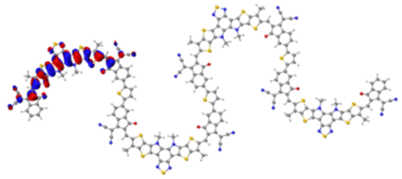 -5.69 eV	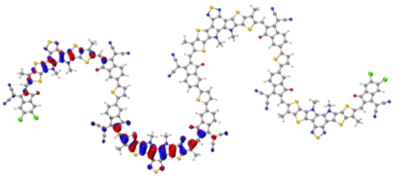 -5.66 eV
H-1	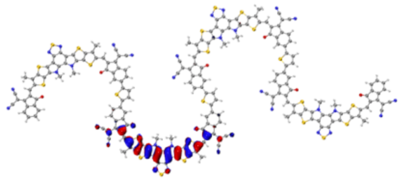 -5.68 eV	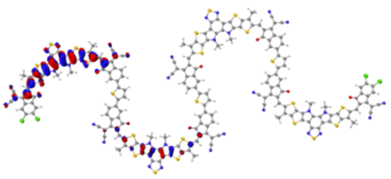 -5.68 eV
H-2	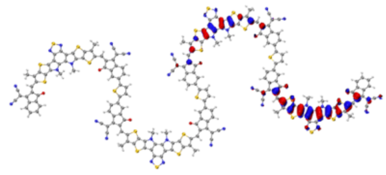 -5.66 eV	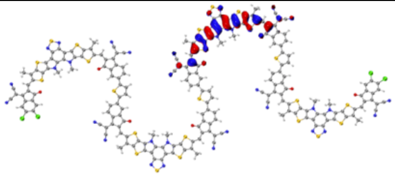 -5.69 eV

H-3	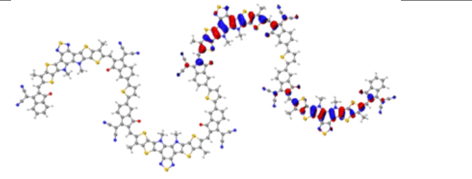<br>-5.60 eV	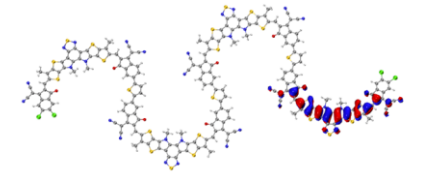<br>-5.75 eV

Supplementary Table 2 | The electronic transition energy E, major orbital transition contribution, Oscillator strength (f), and Sr index for S0→S1 transition calculated.

Systems	Excited State	E (eV)	Orbital transition	Orbital transition contribution (%)	*f*	Sr
PY-BO	S0→S1	2.0127	H-1 → L	19.2076	7.7890	0.7431
			H-2 → L+1	9.4987		
			H-3 → L+2	9.1002		
			H-3 → L+3	8.0923		
			H-2 → L	4.9524		
			H-1 → L+1	4.7002		
			H-3 → L	4.0516		
			H → L+1	3.2737		
			H-1 → L+3	3.1536		
			H-2 → L+3	2.2663		
4Y-BO	S0→S1	2.0083	H → L+1	12.9215	8.7248	0.7445
			H-1 → L+3	9.1199		
			H-1 → L+2	7.8900		
			H-1 → L	7.8456		
			H-3 → L	7.5420		
			H-2 → L+3	4.7629		
			H → L	4.5030		
			H → L+3	3.5511		
			H-2 → L+2	3.5107		
			H-3 → L+2	3.3354		
			H → L+2	2.8494		
			H-2 → L+1	2.4152		

Supplementary Table 3 | The resolved absorptions between 560-900 nm of PY-BO and 4Y-BO in dilute chlorobenzene solutions with identical mass concentration of 1×10^{-3} mg ml.

Acceptor	Component I			Component II			Component III		
	λ_{max} (nm)	Intensity (arb.units)	Area percent (%)	λ_{max} (nm)	Intensity (arb.units)	Area percent (%)	λ_{max} (nm)	Intensity (arb.units)	Area percent (%)
PYBO	799	1.18	24.56	753	0.80	29.03	650	0.75	46.41

4YBO	765	1.91	53.93	699	0.52	10.30	631	0.70	35.77

Supplementary Table 4 | The diffraction peak locations (q), stacking distances (d) full width at half maximum (FWHM) and crystal coherence length (CCL) of the neat acceptor films based on GIWAXS studies.

	q_{xy} (Å^{-1})	d (Å)	FWHM (Å)	CCL (Å)	q_z (Å^{-1})	d (Å)	FWHM (Å)	CCL (Å)
	IP (100)				OOP (010)			
PYBO	0.432	14.54	0.152	41.34	1.647	3.81	0.328	19.16
4YBO	0.429	14.64	0.116	54.17	1.643	3.82	0.258	24.35

CCL obtained using Scherrer's equation (CCL=2π/fwhm) for the peaks in OOP direction.

Supplementary Table 5 | The hole mobility (μ_h) and electron mobility (μ_e) of neat acceptor films and blend films based on SCLC model.

**Film**	**μ_h (cm^2 V^{-1} s^{-1})**	**μ_e (cm^2 V^{-1} s^{-1})**	**μ_h/μ_e**
PY-BO	-	0.69×10^{-4}	-
4Y-BO	-	1.02×10^{-4}	-
PM6:PY-BO	3.40×10^{-4}	2.22×10^{-4}	1.53
PM6:4Y-BO	4.48×10^{-4}	3.45×10^{-4}	1.29

Supplementary Table 6 | The open circuit voltage (V_{OC}), short-circuit current density (J_{SC}), fill factor (FF) and power conversion efficiency (PCE) of PM6:PY-BO and PM6:4Y-BO based solar cells under different optimization conditions.

Blend film	Condition	V_{OC} (V)	J_{SC} (mA cm^{-2})	FF (%)	PCE (%)
PM6:PY-BO	As cast	0.932	22.16	63.73	13.17
	CN	0.929	23.33	72.26	15.66
	2-EN	0.928	23.83	70.00	15.48
PM6:4Y-BO	As cast	0.947	25.85	72.26	17.68
	CN	0.944	26.68	77.57	19.54
	2-EN	0.944	26.59	78.63	19.75

Supplementary Table 7 |The comparison of photovoltaic parameters of high-performance OSCs in previous literatures and this study based on oligomer acceptors.

Number	V_{OC} (V)	J_{SC} (mA cm^{-2})	FF (%)	PCE (%)	Ref.
PM6:2BTP-2F-T	0.911	25.50	78.28	18.19	4
PM6:DY2	0.87	26.60	76.85	17.85	5

PM6:DY3	0.87	26.20	76.21	17.33	5
PM6:DYT	0.94	24.08	76	17.30	6
PM6:DYV	0.93	25.02	78	18.60	6
PM6:DYTVT	0.95	24.82	74	17.68	6
PM6:MYBO	0.877	25.35	77.1	17.123	7
PM6:DYBO	0.968	24.623	75.8	18.082	7
PM6:CH8-1	0.923	24.89	74.2	17.05	8
PM6:EV-i	0.897	26.60	76.56	18.27	9
PM6:TYT	0.964	25.07	75	18.15	10
PM6 : DIBP3F-Se	0.917	25.92	76.1	18.09	11
D18:DYA-I	0.938	25.67	78	18.83	12
PM6 : Tri-Y6-OD	0.916	25.30	77.8	18.03	13
PM6:CH8-4	0.894	26.5	75.5	17.58	14
PM6:DYV	0.910	25.972	76.215	18.013	15
PM6: G-Trimer	0.896	26.75	79.30	19.01	16
PM6:TDY-α	0.864	26.9	78.0	18.1	17
PM6:DY-P2EH	0.905	24.03	78.58	17.09	18
PM6: Dimer-2CF	0.900	26.39	80.03	19.02	19
PBQ*x*-H-TF:dBTIC-*γ*V-BO	0.91	24.52	76.58	17.14	20
PM6:Tet-1	0.919	24.53	76.8	17.32	21
D18:TYT-S	0.964	25.18	77	18.61	22
D18:2Y-wing	0.850	27.66	75.4	17.73	23
PM6:T0	0.923	24.06	77.1	17.12	24
D18:DYF-E	0.938	24.23	75	17.02	25
PM6:CH8-6	0.891	26.23	77.8	18.2	26
PM6:Tri-BT	0.933	25.49	74.9	17.81	27
PM6:Tri-Qx	0.935	25.28	77.5	18.33	27
PM6:TQT	0.944	25.78	76.1	18.52	27
PM6:DYSe-1	0.885	27.51	76.6	18.56	28
PM6:DYSe-2	0.884	27.45	75.2	18.22	28
PM6:Tri-V	0.909	26.13	77.8	18.48	29
PM6:4Y-BO	0.944	26.59	78.63	19.75	This work

Supplementary Table 8 | Key parameters for energy loss of the PM6:PY-BO and PM6:4Y-BO-based devices.

Active layer	E_g (eV)	V_{OC} (V)	E_{loss} (eV)	V_{OC}^{SQ} (V)	ΔE_1 (eV)	ΔE_2 (eV)	ΔE_3 (eV)	EQE_{EL} (%)	E_U (meV)
PM6:PY-BO	1.485	0.929	0.556	1.214	0.271	0.058	0.227	1.5×10^{-2}	24.3
PM6:4Y-BO	1.478	0.944	0.534	1.208	0.270	0.057	0.207	3.2×10^{-2}	23.3

E_g is bandgap of devices. E_{loss} is the total energy loss of devices. V_{OC}^{SQ} is the V_{OC} at the Shockley queisser limit. ΔE_1 is constrained by the Shockley-Queisser limit. ΔE_2 is the inevitable radiative energy loss. ΔE_3 is the non-radiative energy loss. EQE_{EL} is electroluminescence quantum efficiency. E_U is Urbach energy.

Supplementary Table 9 | The exciton dissociation probability (P_{diss}) and charge collection probability (P_{coll}) values of the solar cells.

Active layer	J_{sat} (mA cm^{-2})	J_{sc} (mA cm^{-2})	J_{max} (mA cm^{-2})	P_{diss} (%)	P_{coll} (%)
PM6:PY-BO	24.52	23.33	20.27	95.12	82.70
PM6:4Y-BO	27.31	26.59	24.05	97.36	88.11

J_{sat} is saturated photocurrent density. J_{max} is the current density at maximum output power.

Supplementary Table 10 | GIWAXS data of the blend films based on the resolved (100) diffraction profiles in IP direction.

	PM6 component				Acceptor component			
	q_{xy} (Å^{-1})	d (Å)	FWHM (Å)	CCL (Å)	q_{xy} (Å^{-1})	d (Å)	FWHM (Å)	CCL (Å)
PM6:PY-BO	0.337	18.64	0.053	118.55	0.392	16.03	0.200	31.42
PM6:4Y-BO	0.337	18.64	0.060	104.72	0.420	14.96	0.073	86.07

Supplementary Table 11 | Contact angles and surface energies (γ_s) of the neat films, and the calculated Flory–Huggins interaction parameters (χ) between donor and acceptors.

Film	Contact angle (°)		γ_s (mN m^{-1})	$\chi_{PM6/acceptor}$ (K)
	H_2O	CH_2I_2		
PM6	100.6	49.8	38.4	-
PY-BO	100.1	35.4	45.8	0.33
4Y-BO	99.8	37.5	44.3	0.21

Supplementary references

1. Lu T, Chen F. Multiwfn: A multifunctional wavefunction analyzer. *J Comput Chem* **33**, 580-592 (2012).
2. Lu T, Chen Q. Independent gradient model based on Hirshfeld partition: A new method for visual study of interactions in chemical systems. *J Comput Chem* **43**, 539-555 (2022).
3. Humphrey W, Dalke A, Schulten K. VMD: Visual molecular dynamics. *J Mol Graphics* **14**, 33-38 (1996).
4. Zhang L, Zhang Z, Deng D, Zhou H, Zhang J, Wei Z. "N-π-N" Type Oligomeric Acceptor Achieves an OPV Efficiency of 18.19% with Low Energy Loss and Excellent Stability. *Adv Sci* **9**, 2202513 (2022).
5. Li S, *et al.* Tethered Small-Molecule Acceptors Simultaneously Enhance the Efficiency and Stability of Polymer Solar Cells. *Adv Mater* **35**, 2206563 (2023).
6. Lee J-W, *et al.* Linker Engineering of Dimerized Small Molecule Acceptors for Highly Efficient and Stable Organic Solar Cells. *ACS Energy Lett* **8**, 1344-1353 (2023).
7. Sun C, *et al.* Dimerized small-molecule acceptors enable efficient and stable organic solar cells. *Joule* **7**, 416-430 (2023).
8. Chen H, *et al.* 3D acceptors with multiple A–D–A architectures for highly efficient organic solar cells. *Energy Environ Sci* **16**, 1773-1782 (2023).
9. Zhuo H, *et al.* Giant Molecule Acceptor Enables Highly Efficient Organic Solar Cells Processed Using Non-halogenated Solvent. *Angew Chem Int Ed* **62**, e202303551 (2023).
10. Lee J-W, *et al.* Trimerized small-molecule acceptors enable high-performance organic solar cells with high open-circuit voltage and prolonged life-time. *Energy Environ Sci* **16**, 3339-3349 (2023).
11. Wu J, *et al.* On the Conformation of Dimeric Acceptors and Their Polymer Solar Cells with Efficiency over 18 %. *Angew Chem Int Ed* **62**, e202302888 (2023).
12. Sun C, *et al.* Regiospecific Incorporation of Acetylene Linker in High-Electron Mobility Dimerized Acceptors for Organic Solar Cells with High Efficiency (18.8%) and Long 1-Sun Lifetime (> 5000 h). *Adv Energy Mater* **13**, 2301283 (2023).
13. Zhang C, *et al.* Facile, Versatile and Stepwise Synthesis of High-Performance Oligomer Acceptors for Stable Organic Solar Cells. *Angew Chem Int Ed Engl* **62**, e202308595 (2023).
14. Chen H, *et al.* Terminally Chlorinated and Thiophene-linked Acceptor-Donor-Acceptor Structured 3D Acceptors with Versatile Processability for High-efficiency Organic Solar Cells. *Angew Chem Int Ed* **62**, e202307962 (2023).
15. Fu H, *et al.* Modular-Approach Synthesis of Giant Molecule Acceptors via Lewis-Acid-Catalyzed Knoevenagel Condensation for Stable Polymer Solar Cells. *Angew Chem Int Ed* **62**, e202306303 (2023).
16. Wang C, *et al.* Unique assembly of giant star-shaped trimer enables non-halogen solvent-fabricated, thermal stable, and efficient organic solar cells. *Joule* **7**, 2386-2401 (2023).
17. Bai Y, *et al.* Geometry design of tethered small-molecule acceptor enables highly stable and efficient polymer solar cells. *Nat Commun* **14**, 2926 (2023).
18. Zhang M, *et al.* Tethered Small-Molecule Acceptor Refines Hierarchical Morphology in Ternary Polymer Solar Cells: Enhanced Stability and 19% Efficiency. *Adv Mater* **36**, 2308606 (2024).
19. Lv M, *et al.* Strengthening the Hetero-Molecular Interactions in Giant Dimeric Acceptors Enables Efficient Organic Solar Cells. *Adv Mater* **36**, 2310046 (2024).
20. Tan P, *et al.* Enhanced Performance and Stability of Q-PHJ Devices through Strategic Placement of Dimerized Acceptors. *Adv Funct Mater* **34**, 2305608 (2024).
21. Zhang C, *et al.* Simple and Efficient Synthesis of Novel Tetramers with Enhanced Glass Transition Temperature for High-Performance and Stable Organic Solar Cells. *Angew Chem Int Ed Engl* **63**, e202316295 (2024).

22. Lee J-W*, et al.* Design of Star-Shaped Trimer Acceptors for High-Performance (Efficiency > 19%), Photostable, and Mechanically Robust Organic Solar Cells. *Adv Energy Mater* **14**, 2303872 (2024).
23. Yi F*, et al.* Non-Fully Conjugated Dimerized Giant Acceptors with Different Alkyl-Linked Sites for Stable and 19.13 % Efficiency Organic Solar Cells. *Angew Chem Int Ed* **63**, e202319295 (2024).
24. Li Y*, et al.* Conjugation-Broken Dimer Acceptors Enable High-Efficiency, Stable, and Flexibility-Robust Organic Solar Cells. *Adv Mater* **36**, 2403890 (2024).
25. Oh S*, et al.* Impact of Linker Engineering in Core-Linked Dimeric Acceptors for High-Performance Organic Solar Cells. *Adv Funct Mater* **34**, 2406501 (2024).
26. Zhang Z*, et al.* Rational design of flexible-linked 3D dimeric acceptors for stable organic solar cells demonstrating 19.2% efficiency. *Energy Environ Sci* **17**, 5719-5729 (2024).
27. Liu J*, et al.* A quinoxaline–benzothiadiazole heterotrimer for organic solar cells with extraordinary efficiency and stability. *Energy Environ Sci* **17**, 3641-3650 (2024).
28. Bai Y*, et al.* Multi-Selenophene Strategy Enables Dimeric Acceptors-Based Organic Solar Cells with over 18.5% Efficiency. *Adv Energy Mater* **14**, 2400938 (2024).
29. Song J*, et al.* Non-halogenated Solvent-Processed Organic Solar Cells with Approaching 20 % Efficiency and Improved Photostability. *Angew Chem Int Ed* **63**, e202404297 (2024).
