# Supplementary material for: Polymer-like tetramer acceptor enables stable and 19.75% efficiency binary organic solar cells
Source: Nat Commun. 2025 Feb 20;16:1784. doi: 10.1038/s41467-025-57118-9 (PMC11840019; doi:10.1038/s41467-025-57118-9)
Supplement: Supplementary file 2 — Solar Cells Reporting Summary [file 41467_2025_57118_MOESM2_ESM.pdf]

nature portfolio

Corresponding author(s): Xichang Bao

Solar Cells Reporting Summary

Nature Portfolio wishes to improve the reproducibility of the work that we publish. This form is intended for publication with all accepted papers reporting the characterization of photovoltaic devices and provides structure for consistency and transparency in reporting. Some list items might not apply to an individual manuscript, but all fields must be completed for clarity.

For further information on Nature Research policies, including our data availability policy, see Authors & Referees.

Experimental design

Please check the following details are reported in the manuscript, and provide a brief description or explanation where applicable.

1. Dimensions

Area of the tested solar cells

☒ Yes — Active area of the tested solar cells is 0.0936 cm-2.

☐ No — *Explain why this information is not reported/not relevant.*

Method used to determine the device area

☒ Yes — she active area was determined by the crossed area of counter electrode and ITO stripe.

☐ No — *Explain why this information is not reported/not relevant.*

2. Current-voltage characterization

Current density-voltage (J-V) plots in both forward and backward direction

☐ Yes

☒ No — Organic solar cells do not have hysteresis problem. And we only scan the device inforward direction.

Voltage scan conditions

☒ Yes — Section "Supplementary Methods".

☐ No — *Explain why this information is not reported/not relevant.*

Test environment

☒ Yes — Section "Supplementary Methods".

☐ No — *Explain why this information is not reported/not relevant.*

Protocol for preconditioning of the device before its characterization

☐ Yes — *Provide a description of the protocol.*

☒ No — No preconditioning protocol.

Stability of the J-V characteristic

☐ Yes — *Provide a description of the method used. The stability of the J-V characteristic can be verified with time evolution of the maximum power point or with the photocurrent at maximum power point; see ref. 5 for details.*

☒ No — Organic solar cells are rarely tested for stability of the J-V characteristics. The long-term and bending stability of the devices are measured.

3. Hysteresis or any other unusual behaviour

Description of the unusual behaviour observed during the characterization

☐ Yes — *Provide a description of hysteresis or any other unusual behaviour observed during the characterization.*

☒ No — No hysteresis or any other unusual behavior was observed during the characterization, organic solar cells do not have hysteresis problems.

Related experimental data

☐ Yes — *Provide a description of the related experimental data.*

☒ No — No hysteresis or any other unusual behavior was observed during the characterization.

4. Efficiency

External quantum efficiency (EQE) or incident photons to current efficiency (IPCE)

☒ Yes — See Fig. 3c.

☐ No — *Explain why this information is not reported/not relevant.*

A comparison between the integrated response under the standard reference spectrum and the response measure under the simulator

☒ Yes — See Table 2.
☐ No — *Explain why this information is not reported/not relevant.*

For tandem solar cells, the bias illumination and bias voltage used for each subcell

☐ Yes — *Provide a description of the measurement conditions.*
☒ No — No tandem solar cells was reported

5. Calibration

Light source and reference cell or sensor used for the characterization

☒ Yes — Section "Supplementary Methods".
☐ No — *Explain why this information is not reported/not relevant.*

Confirmation that the reference cell was calibrated and certified

☒ Yes — Section "Supplementary Methods".
☐ No — *Explain why this information is not reported/not relevant.*

Calculation of spectral mismatch between the reference cell and the devices under test

☐ Yes — *Provide a value of the spectral mismatch and/or a description of how it has been taken into account in the measurements.*
☒ No — No spectral mismatch calculation was performed in our lab.

6. Mask/aperture

Size of the mask/aperture used during testing

☒ Yes — Section "Supplementary Methods".
☐ No — *Explain why this information is not reported/not relevant.*

Variation of the measured short-circuit current density with the mask/aperture area

☐ Yes — *Report the difference in the short-circuit current density values measured with the mask and aperture area.*
☒ No — We didn't measure the solar cells with apertures of different areas.

7. Performance certification

Identity of the independent certification laboratory that confirmed the photovoltaic performance

☒ Yes — The certification report from CPVT, China.
☐ No — *Explain why this information is not reported/not relevant.*

A copy of any certificate(s)

☒ Yes — See Fig. S18.
☐ No — *Explain why this information is not reported/not relevant.*

8. Statistics

Number of solar cells tested

☒ Yes — See Fig.3b, Table 2.
☐ No — *Explain why this information is not reported/not relevant.*

Statistical analysis of the device performance

☒ Yes — See Fig.3b, Table 2.
☐ No — *Explain why this information is not reported/not relevant.*

9. Long-term stability analysis

Type of analysis, bias conditions and environmental conditions

☒ Yes — Section "Supplementary Methods" and Fig. 6 and Fig. S37.
☐ No — *Explain why this information is not reported/not relevant.*
