# Supplementary material for: Polymer-like tetramer acceptor enables stable and 19.75% efficiency binary organic solar cells
Source: Nat Commun. 2025 Feb 20;16:1784. doi: 10.1038/s41467-025-57118-9 (PMC11840019; doi:10.1038/s41467-025-57118-9)
Supplement: Supplementary file 3 — Transparent Peer Review file [file 41467_2025_57118_MOESM3_ESM.pdf]

nature portfolio

Peer Review File

Polymer-Like Tetramer Acceptor Enables Stable and 19.75% Efficiency Binary Organic Solar Cells

Corresponding Author: Professor Xichang Bao

This file contains all reviewer reports in order by version, followed by all author rebuttals in order by version.

Version 0:

Reviewer comments:

Reviewer #1

(Remarks to the Author)
The manuscript reports a new linear tetramer oligomer acceptor, 4Y-BO. Compared to the polymer acceptor PY-BO, 4Y-BO demonstrates advantages of homogeneous crystallinity kinetics, enhanced molecular ordering, and more efficient exciton and charge transport properties. Furthermore, 4Y-BO-based solar cell achieved a notable efficiency of 19.75%, surpassing that of PM6:PY-BO. Although the efficiency is impressive, the manuscript lacks of significant finding and novelty. It is not suitable for publication in Nature Communications.
1.The manuscript introduces a new tetramer. However, similar tetramers have already been reported in the existing literature (Nat. Commun. 2023 14, 7996; Adv. Funct. Mater. 2024, 2409764). The authors have merely optimized the side chains and end groups, and the comparison materials are also quite similar. From these aspects, the novelty is of this work is quite limited.
2.The device based on 4Y-BO exhibited much higher efficiency than those similar tetramers-based devices reported in the literature. The authors should explain the reason.
3.An efficiency of 19.75% is the highest efficeicy for OSCs based on oligomer acceptors. Such a record-high efficiency should be certified in a third official party.
4.Generally, the up-shift in the LUMO level of the acceptor is expected to result in a higher open circuit voltage of the corresponding device. However, it is intriguing that the 4Y-BO-based device exhibits a higher voltage than the PY-BO-based device. What is the reason?
5.The literature has reported that the efficiency of PY-BO-based device has surpassed 19%, much higher than the15.66% efficiency reported in this work. The authors should explain the reason.
6.The author states that the tetramer 4Y-BO was obtained with a significant yield of 80%. To enhance the credibility, please provide supporting data, such as yield results obtained from HPLC or LC-MS analysis.
7.The article by Li et al. (Nat Commun 14, 7996 (2023).) shows when under the continuous thermal annealing at 90 °C, the T90 based on their tetramer acceptor OSC was just over ten hours, while in this manuscript the T80 based on the PM6:4Y-BO OSC was 2125h. How does the author comment on this difference?
8.Most literature report that the Tgs of trimers falls within the range of 150-180 °C (Adv. Funct. Mater. 2024, 2410092; Energy Environ. Sci., 2023,16, 3339; Nat Energy 2022, 7, 1180) Why are the Tg values for 4Y-BO and PY-BO mentioned in the main text significantly lower?

Reviewer #2

(Remarks to the Author)
Wang et al reported a polymer tetramer acceptor, applying the corresponding polymer as a control, they deeply investigated the advantages of the tetramer with various characterizations, and also reported an excellent efficiency of 19.75%. However, there are still the following questions that should be clearly explained before acceptance:
1) As in the paper, the MW of polymer acceptor is quite low (almost 4 monomers), and hence, the comparison between polymer and tetramer in this paper is indeed a combination of purity (or molecular distribution) in aggregation and fluorination in structure. So, in your opinion, what would the comparison happen, especially for the stability and mechanical flexibility between the tetramer and a higher MW (e.g. > 20K)
2)For the absorption spectrum, compared the polymers (ca. four acceptors) with tetramer, why would polymers result in serious interchain entanglements or serious pre-aggregation, but tetramer could lead to a uniform distribution in the dilute solution? Is it just because of their different aggregation (such H and J)? The reasons for the redshifts of the polymer’s

spectrum in solution or different redshifts from solution to films should be investigated more clearly.
3) In the stability part, both of the Tg exceed much above the thermal test temperature. What’s your opinion on the much better thermal stability of tetramer blends, especially its much -lower burn-in loss?
4)In small molecules, fluorinating the end-capped group normally leads to a larger non-radiative energy loss, but the fluorinated tetramer could obtain a reduced non-radiative energy loss in this study. What’s your explanation?

Reviewer #3

(Remarks to the Author)
Indefinite structure and excessive entanglement of polymers result in large batch differences and limited photovoltaic performance of all-polymer solar cells (all-PSCs), which is a long-term challenge restricting its industrialization. Developing high molecular-weight giant molecule acceptors with definite chemical structures would be a potential alternative approach. In this manuscript, the authors reported a linear giant molecule acceptor 4Y-BO for constructing efficient and long-term stable organic solar cells (OSCs). A control polymeric acceptor namely PY-BO carrying identical main backbone and side groups, is synthesized in order to reach a comprehensive comparison in term of molecular aggregation, photovoltaic properties, as well as device thermal/mechanical stabilities. Based on measurements and analysis, the author disclosed that 4Y-BO based OSCs have achieved higher PCE of up to 19.75% with more preferred narrow batch-to-batch variations than the polymer PY-BO based device. In addition, the 4Y-BO exhibits high thermal and mechanical stability of corresponding devices. This work comprehensively compared the performance differences between the giant molecule acceptor and polymer acceptor with comparable molecular weights, fully affirming the advantages and enormous development potential of high molecular-weight giant molecule acceptors. Therefore, I recommend the acceptance of this manuscript for the publication in Nature Communications after some minor revisions addressing following technique concerns and comments.

1. Fig. S8 shows that the HOMO and LUMO orbitals of the 4Y-BO are distributed on different units with less overlap and coupling, which typically gave small extinction coefficient for absorption. In addition, the HOMO electron cloud density of PY-BO is located on one Y nucleus, while the HOMO of 4Y-BO is located on 2Y cores. Please further confirm the simulation results and provide some possible explanations.

2. The authors should include color bars in Fig. S4-S6 indicating the negative and positive values, and provide a detailed description of the methods and boundary conditions used for density functional theory simulations.

3. In order to more intuitively and clearly compare the intermolecular differences between the donor and the giant molecule acceptor or polymer acceptor in the blend film, it will be very convincing to perform density functional theory simulations on them and incorporate corresponding discussions about the results.

4. The author compared the pre-aggregation characteristics of 4Y-BO and PY-BO by temperature-dependent absorption measurements, why the 4Y-BO shows mildly enlarged variations of wavelength and intensity, but the pre-aggregation characteristics reduced? Please provide some possible explanations.

5. The authors emphasize that the PM6:4Y-BO blend film forms a more uniform crystalinity and clear fiber structure, which is one critical factor to its outstanding exciton/charge properties and photovoltaic performance. Supplementing the contact angles of the employed materials and Flory-Huggins interaction parameters should be helpful to provide an in-depth illustration about the morphology landscape.

6. Please clarify if the external quantum efficiency (EQE) spectra align with the absorption spectra of the blend film, and provide the absorption spectra of the blend films.

7. The solid and dashed lines in the line-cut profiles (Fig. 2i and 5c) should be clearly labeled for better comparisons.

Version 1:

Reviewer comments:

Reviewer #1

(Remarks to the Author)
In the revised manuscript, the authors added more results, including the certified efficiency to address my concerns. Before publication, two more questions should be clarified.
1. As shown in Figure 2i and Table R1, compared to QY, 4Y-BO shows improved molecular ordering and better crystallinity. However, as shown in Figure 3f and Table R2, 4Y-BO has higher EQEel than that of QY. The authors should do more experiments to explain the reason.
2.4Y-BO shows much better thermal stability than QY. How about the photostability?

Reviewer #2

(Remarks to the Author)
The manuscript has been revised carefully, which could be accepted for publication.

Reviewer #3

(Remarks to the Author)
The authors have revised the manuscript according to the reviewers' comments and revision opinions. Now the revised manuscript can be accepted for publication on Nature Communications.

Version 2:

Reviewer comments:

Reviewer #1

(Remarks to the Author)
My concern has been well addressed by the authors and this paper can be accepted for publication.

Open Access This Peer Review File is licensed under a Creative Commons Attribution 4.0 International License, which permits use, sharing, adaptation, distribution and reproduction in any medium or format, as long as you give appropriate credit to the original author(s) and the source, provide a link to the Creative Commons license, and indicate if changes were made.
In cases where reviewers are anonymous, credit should be given to 'Anonymous Referee' and the source.
The images or other third party material in this Peer Review File are included in the article's Creative Commons license, unless indicated otherwise in a credit line to the material. If material is not included in the article's Creative Commons license and your intended use is not permitted by statutory regulation or exceeds the permitted use, you will need to obtain permission directly from the copyright holder.
To view a copy of this license, visit https://creativecommons.org/licenses/by/4.0/

Our point-by-point responses to reviewers' comments are listed as follows:

THE REVIEWER 1#'S REPORT

= = = = = = = = = = = = = = = = = = =

The manuscript reports a new linear tetramer oligomer acceptor, 4Y-BO. Compared to the polymer acceptor PY-BO, 4Y-BO demonstrates advantages of homogeneous crystallinity kinetics, enhanced molecular ordering, and more efficient exciton and charge transport properties. Furthermore, 4Y-BO-based solar cell achieved a notable efficiency of 19.75%, surpassing that of PM6:PY-BO. Although the efficiency is impressive, the manuscript lacks of significant finding and novelty. It is not suitable for publication in Nature Communications.

Response: We are sorry for our manuscript not satisfying to you, but still sincerely appreciate your valuable comments and suggestions. In the following, we have tried our best to address your concerns/comments, and extensively revise our manuscript by providing additional experimental results (Include: *(i)* a third official party certification about our reported efficiency of PM6:4Y-BO solar cell (certified efficiency: 19.58%), *(ii)* density functional theory simulations about D/A and A/A interactions and corresponding analysis, *(iii)* energy loss analysis with highly-sensitive external quantum efficiency (sEQE), electroluminescence (EL) spectra, and electroluminescence quantum efficiency (EQE_{EL}) measurements, etc.) and more in-depth discussions. In the following, we provide detailed responses to the comments point-by-point. We hope all the revisions have addressed your concerns and comments.

Q1. The manuscript introduces a new tetramer. However, similar tetramers have already been reported in the existing literature (Nat. Commun. 2023 14, 7996; Adv. Funct. Mater. 2024, 2409764). The authors have merely optimized the side chains and end groups, and the comparison materials are also quite similar. From these aspects, the novelty is of this work is quite limited.

Response: Thanks for your comment. As we know, high molecular-weight oligomers with defined chemical structures are good alternatives to avoid the batch differences and chaotic stacking of polymers. Although there have been some studies about tetramer acceptors as you have suggested, most of them were equipped unremarkable photovoltaic performance (below 18%). Unfortunately, the lack of efficiency breakthrough in the tetramer or greater oligomer-based devices over a long period of time may raise concerns about their potential.

Encouragingly, the device efficiency based on tetramer acceptor has been greatly enhanced (19.75% (certified to 19.58%)). Meanwhile, the stability and flexibility of the devices have been significantly improved and we also systematically revealed their key mechanisms. The fruitful findings we disclosed in this manuscript will undoubtedly bring renewed attention to the tetramer or larger molecular-weight oligomers. Our detail contributions can be concluded as follows:

(1) As is well known, the biggest breakthrough and innovation in the photovoltaic field is the improvement of efficiency. Here, we reported a highly efficient tetramer acceptor by simple endcap and sidechain modification, and realized an excellent PCE of 19.75% (certified to 19.58%), which greatly refreshed the efficiencies of oligomer acceptors (including dimer, trimer, tetramer, and larger oligomers). Furthermore, the outstanding efficiency of 4Y-BO can be attributed to the synergistic effect of oligomer endcaps (chlorination) and sidechains (shortened), which also suggests the influence of key factors on device performance.

(2) We rationally conducted a detailed and systematic comparison about the tetramer and polymer acceptors with identical backbones, and fully reveal their distinctions on aggregation characteristics, photovoltaic performance, thermal stability, and mechanical stability. It can be considered that high molecular weight oligomeric acceptors have distinctive advantages in achieving efficient and stable organic solar cells.

(3) Flexibility is the most important characteristic of organic photovoltaics. We reported, for the first time, the promising relationship between resilience characteristics and mechanical stability of flexible device performance with respect to the stress-strain curve, and excellent flexible devices require high elastic deformation (mechanical stability capability) and elastic modulus (rebound rate). These new findings have great guiding significance on the development of state-of-the-art flexible photovoltaics.

Q2. The device based on 4Y-BO exhibited much higher efficiency than those similar tetramers-based devices reported in the literature. The authors should explain the reason.

Response: Thank you very much for this concern, which allows us to revisit our material design strategy. Based on the molecular structures of tetramers in our work and literature, the outstanding efficiency of 4Y-BO is probably attributed to the synergistic effect of oligomer endcaps (chlorination) and sidechains (shortened), which collectively refine the molecular self-assembly, donor/acceptor interactions, heterojunction textures, and thereby charge transport and recombination.

Taking the endcap as an example, we employed chlorination to terminate the largely extended tetramer backbone. The halogen modification has been well-recognized to promote molecular packing and crystallinity (such as ITIC *vs* IT-4F, and PBDB-T *vs* PBDB-TF material systems). As **Table R1** suggests, the comparison of 4Y-BO and literatures based on molecular distances (d-spacing) and crystal coherence lengths (CCLs) again confirm the above results. In the following, we further disclosed the impacts of chlorination on donor/acceptor intermolecular interactions based on DFT simulations. As **Fig. R1** depicts, compared to PM6/QY and PM6/TetA pairs in literatures, the PM6/4Y-BO demonstrates mildly stronger intermolecular interactions, which are favored to enhance blend compatibility, and inhibit the excessive entanglement of single component to form suitable phase-separations and fibrous interpenetrating networks. Perhaps the combined effects of these multiple improvements have afforded the PCE breakthrough of 4Y-BO based solar cells.

Table R1 Molecular packing parameters for neat films derived from GIWAXS fitting.

Acceptor	d-spacing (010) (Å)	CCL (010) (Å)	Ref.
QY	3.832	20.86	*Nat. Commun.* **14**, 7996 (2023)
TetA	3.9	18	*Adv. Funct. Mater.* **34**, 2409764 (2024)
4Y-BO	3.82	24.35	This study

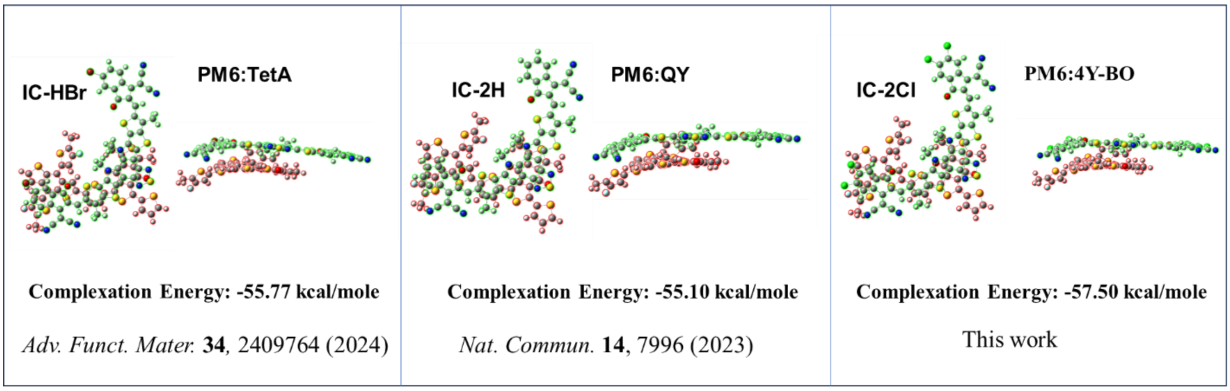

Fig. R1 Optimized intermolecular packing diagrams by density functional theory simulation. To simplify the calculation, we extracted the repeating unit of PM6 and single Y units of the tetramers (with Cl or H modified endcaps) to perform the simulations.

Q3. An efficiency of 19.75% is the highest efficeicy for OSCs based on oligomer acceptors. Such a

record-high efficiency should be certified in a third official party.

Response: Thanks for your kind suggestion and we agree with you. According to your suggestion, we have conducted the efficiency certification at the National Center of Inspection on Solar Photovoltaic Product Quality (CPVT), China. The certified PCE of the unencapsulated PM6:4Y-BO device reaches 19.58%, with open-circuit voltage (V_{OC}) of 0.940 V, short-circuit current density (J_{SC}) of 26.94 mA cm^{-2}, and fill factor (FF) of 77.33%. The certified PCE reaches 99.14% of the reported efficiency in the manuscript. The mild efficiency loss is ascribed to the device storage (15 hrs between preparation and test), transportation, and measurement under ambient atmosphere for the unencapsulated device. The certification report was provided below as **Fig. R2**, which was also collected into the revised Supporting Information as **Supplementary Fig. 15.** ***(Page S13)***

According to the certification report, we have incorporated the certified efficiency of PM6:4Y-BO into the revised manuscript and updated the **Table 2** ***(Page 12, revised manuscript)*** as follows:

Table 2. Photovoltaic parameters of PM6:PY-BO and PM6:4Y-BO based OSCs.

Active layer	V_{OC} (V)	J_{SC} (mA cm^{-2})	J_{EQE} (mA cm^{-2})	FF (%)	PCE[a] (%)	E_{loss} (eV)
PM6:PY-BO	0.929 (0.926±0.003)	23.33 (23.19±0.41)	22.47	72.26 (70.25±0.73)	15.66 (15.16±0.57)	0.556
PM6:4Y-BO	0.944 (0.943±0.003)	26.59 (26.58±0.21)	25.62	78.63 (77.96±0.52)	19.75 (19.55±0.23)	0.534
PM6:4Y-BO[b]	0.940	26.94	-	77.33	19.58	-

[a] Average parameters with standard deviations obtained from at least ten devices. [b] Certificated by National Center of Inspection on Solar Photovoltaic Products Quality (CPVT).

We have also incorporated some relevant discussions into the revised manuscript as "Notably, the champion efficiency of PM6:4Y-BO device has been certified by National Center of Inspection on Solar Photovoltaic Products Quality (CPVT), China. The certified PCE of PM6:4Y-BO solar cell reached 19.58%, with V_{OC} of 0.940 V, J_{SC} of 26.94 mA cm^{-2}, and FF of 77.33% (Table 2, Supplementary Fig. 15)". ***(Page 12, revised manuscript)***

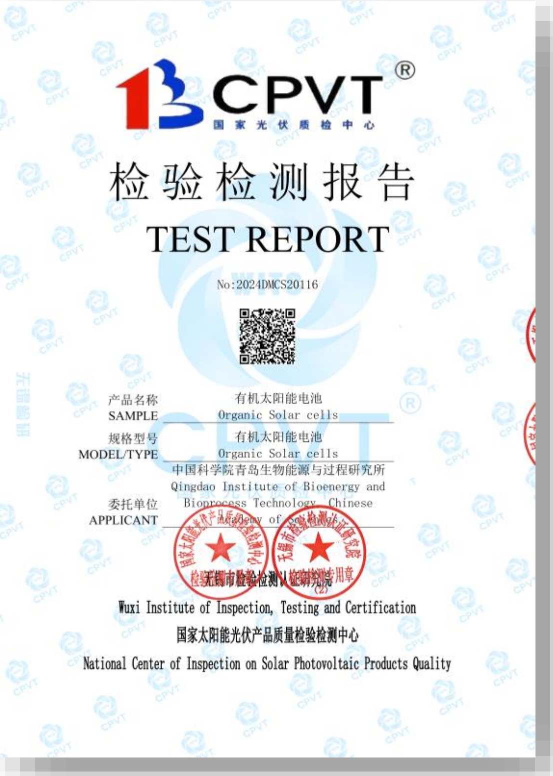

CPVT®

国家光伏质检中心

检验检测报告

TEST REPORT

No:2024DMCS20116

产品名称 SAMPLE	有机太阳能电池 Organic Solar cells
规格型号 MODEL/TYPE	有机太阳能电池 Organic Solar cells
委托单位 APPLICANT	中国科学院青岛生物能源与过程研究所 Qingdao Institute of Bioenergy and Bioprocess Technology, Chinese Academy of [illegible]

无锡市检验检测认证研究院

Wuxi Institute of Inspection, Testing and Certification

国家太阳能光伏产品质量检验检测中心

National Center of Inspection on Solar Photovoltaic Products Quality

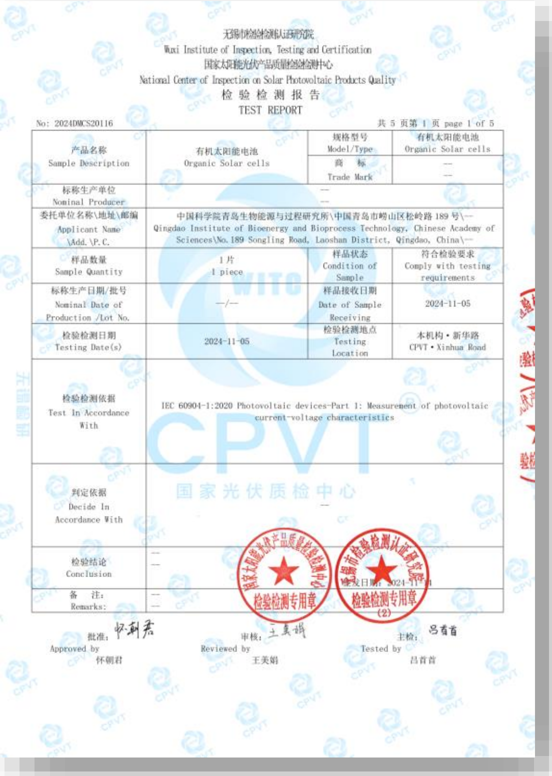

无锡市检验检测认证研究院

Wuxi Institute of Inspection, Testing and Certification

国家太阳能光伏产品质量检验检测中心

National Center of Inspection on Solar Photovoltaic Products Quality

检验检测报告

TEST REPORT

No: 2024DMCS20116 共 5 页第 1 页 page 1 of 5

产品名称 Sample Description	有机太阳能电池 Organic Solar cells	规格型号 Model/Type	有机太阳能电池 Organic Solar cells
		商标 Trade Mark	— —
标称生产单位 Nominal Producer	— —		
委托单位名称\地址\邮编 Applicant Name \Add.\P.C.	中国科学院青岛生物能源与过程研究所\中国青岛市崂山区松岭路 189 号\-- Qingdao Institute of Bioenergy and Bioprocess Technology, Chinese Academy of Sciences\No.189 Songling Road, Laoshan District, Qingdao, China\--		
样品数量 Sample Quantity	1 片 1 piece	样品状态 Condition of Sample	符合检验要求 Comply with testing requirements
标称生产日期/批号 Nominal Date of Production /Lot No.	—/—	样品接收日期 Date of Sample Receiving	2024-11-05
检验检测日期 Testing Date(s)	2024-11-05	检验检测地点 Testing Location	本机构 • 新华路 CPVT • Xinhua Road
检验检测依据 Test In Accordance With	IEC 60904-1:2020 Photovoltaic devices-Part 1: Measurement of photovoltaic current-voltage characteristics		
判定依据 Decide In Accordance With	—		
检验结论 Conclusion	— —		
备注: Remarks:	— —		

签发日期: 2024-11-[illegible]

批准: Approved by 怀朝君 审核: Reviewed by 王美娟 主检: Tested by 吕首首

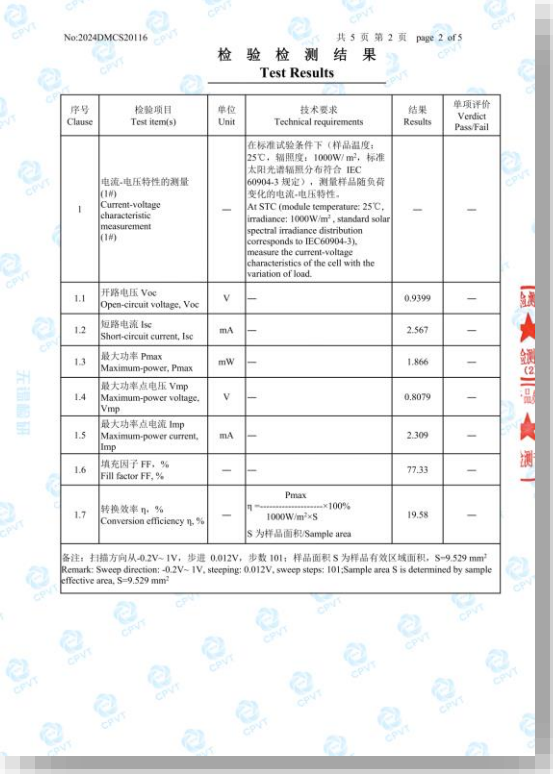

No:2024DMCS20116 共 5 页 第 2 页 page 2 of 5

检验检测结果

Test Results

序号 Clause	检验项目 Test item(s)	单位 Unit	技术要求 Technical requirements	结果 Results	单项评价 Verdict Pass/Fail
1	电流-电压特性的测量 (1#) Current-voltage characteristic measurement (1#)	—	在标准试验条件下（样品温度：25℃，辐照度：1000W/ m²，标准太阳光谱辐照分布符合 IEC 60904-3 规定），测量样品随负荷变化的电流-电压特性。At STC (module temperature: 25℃, irradiance: 1000W/m², standard solar spectral irradiance distribution corresponds to IEC60904-3), measure the current-voltage characteristics of the cell with the variation of load.	—	—
1.1	开路电压 Voc Open-circuit voltage, Voc	V	—	0.9399	—
1.2	短路电流 Isc Short-circuit current, Isc	mA	—	2.567	—
1.3	最大功率 Pmax Maximum-power, Pmax	mW	—	1.866	—
1.4	最大功率点电压 Vmp Maximum-power voltage, Vmp	V	—	0.8079	—
1.5	最大功率点电流 Imp Maximum-power current, Imp	mA	—	2.309	—
1.6	填充因子 FF，% Fill factor FF, %	—	—	77.33	—
1.7	转换效率 η，% Conversion efficiency η, %	—	$\eta = \frac{P_{max}}{1000W/m^2 \times S} \times 100\%$ S 为样品面积/Sample area	19.58	—

备注：扫描方向从-0.2V~ 1V，步进 0.012V，步数 101；样品面积 S 为样品有效区域面积，S=9.529 mm²
Remark: Sweep direction: -0.2V~ 1V, steeping: 0.012V, sweep steps: 101;Sample area S is determined by sample effective area, S=9.529 mm²

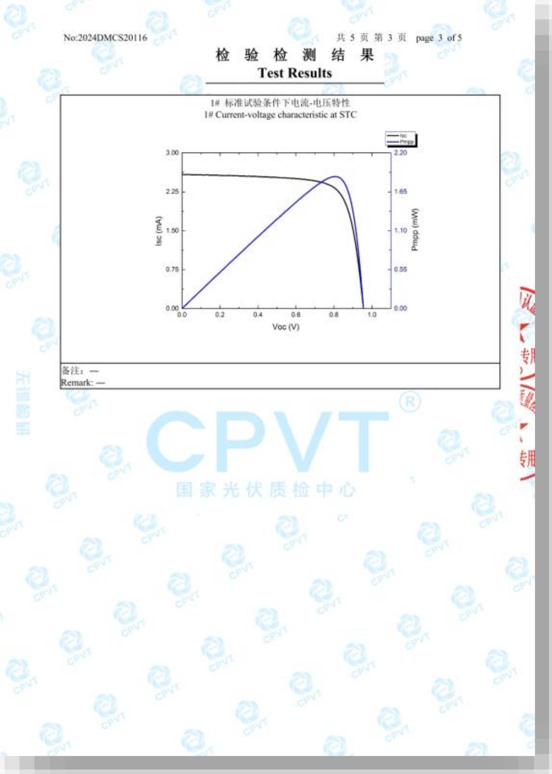

No:2024DMCS20116 共 5 页 第 3 页 page 3 of 5

检验检测结果

Test Results

1# 标准试验条件下电流-电压特性

1# Current-voltage characteristic at STC

备注：—

Remark: —

Fig. R2 The certification report from CPVT, China, as for PM6:4Y-BO device.

Q4. Generally, the up-shift in the LUMO level of the acceptor is expected to result in a higher open circuit voltage of the corresponding device. However, it is intriguing that the 4Y-BO-based device exhibits a higher voltage than the PY-BO-based device. What is the reason?

Response: Thanks for noticing this and sharing your opinion. As we know, the open-circuit voltages (V_{OC}) of OSCs are influenced by multiple factors, including the dielectric constant of materials, energy gap between HOMO level of donor and LUMO level of acceptor, heterojunction textures, charge generation and recombination, et al. In order to clarify this point, we further investigated the energy loss (E_{loss}) of these acceptors-based devices. The highly-sensitive external quantum efficiency (sEQE) and electroluminescence (EL) spectra were measured as depicted in **Fig. R3** below, with detailed parameters collected in **Table R2**. The E_gs of PM6:PY-BO and PM6:4Y-BO based devices are determined to be 1.485 eV and 1.478 eV, respectively. According to well-documented methods in the literature (*Energy Environ. Sci.* **17**, 6844-6855 (2024)), the E_{loss} can be described as three parts based on the detailed balance theory: $E_{loss}=\Delta E_1+\Delta E_2+\Delta E_{nr}$, where ΔE_1 is constrained by the Shockley-Queisser limit, ΔE_2 is the inevitable radiative energy loss, and ΔE_3 is the non-radiative energy loss. Based on the measurements, the ΔE_1 of PM6:PY-BO and PM6:4Y-BO-based devices are determined to be 0.271 and 0.270 eV, and ΔE_2 are 0.058 and 0.057 eV, respectively. It is worth noting that the ΔE_{nr} of the two solar cells, obtained from EQE_{EL} measurements, are markedly different with 0.227 and 0.207 eV for PM6:PY-BO and PM6:4Y-BO-based devices, respectively. The lower ΔE_{nr} of PM6:4Y-BO should be partly attributed to the improved molecular ordering and more appropriate phase separation, which promote charge transport, reduce charge accumulation and recombination at interfaces. As a result, the overall E_{loss} of PM6:4Y-BO device (0.534 eV) is smaller than that of PM6:PY-BO (0.556 eV), which well explains the greater V_{OC} of PM6:4Y-BO solar cells.

Based on the above supplementary studies, we have incorporated the **Fig. R3** into the revised manuscript as **Figs. 3d-3f** ***(Page 11, revised manuscript)***. The **Table R2** was collected in **Supplementary Table 8** ***(Page S24, Supplementary Information)***

Additionally, we have incorporated some discussions about the energy loss studies into the revised manuscript as "In addition, we highlight the greater V_{OC} of PM6:4Y-BO device in spite of the slightly deeper LUMO level of 4Y-BO (Table 1). In order to clarify this point, we move to explore the energy loss (E_{loss}) of solar cells. Highly-sensitive external quantum efficiency (sEQE) and electroluminescence (EL) spectra were measured as depicted in Fig. 3d-3f, with detailed parameters

collected in Supplementary Table 8. The E_gs of PM6:PY-BO and PM6:4Y-BO devices are determined to be 1.485 eV and 1.478 eV, respectively. The E_{loss} can be described as three parts based on the detailed balance theory: $E_{loss}=\Delta E_1+\Delta E_2+\Delta E_{nr}$, where ΔE_1 is constrained by the Shockley-Queisser limit, ΔE_2 is the inevitable radiative energy loss, and ΔE_3 is the non-radiative energy loss.[50] In this scenario, the ΔE_1 of PM6:PY-BO and PM6:4Y-BO-based devices are 0.271 and 0.270 eV, and ΔE_2 are 0.058 and 0.057 eV, respectively. It is worth noting that the ΔE_{nr} of the two solar cells, obtained from EQE_{EL} spectra, are markedly different with 0.227 and 0.207 eV for PM6:PY-BO and PM6:4Y-BO devices, respectively. In order to further expound the reduced non-radiative energy loss of PM6:4Y-BO device, we measured the photoluminescence quantum yield (PLQY) of PY-BO and 4Y-BO neat films. As shown in Supplementary Fig. 17, the PLQY of PY-BO and 4Y-BO are determined to be 8.26% and 1.53%, respectively. As previously reported,[51] the increased PLQY of 4Y-BO should be one important factor for the reduction of ΔE_{nr}. Besides, the lower ΔE_{nr} of PM6:4Y-BO device should be partly attributed to the more ordered molecular stacking and appropriate phase-separations, which promote charge transfer and separation, reduce charge accumulation and recombination at the interfaces. As a result, the overall E_{loss} of PM6:4Y-BO device (0.534 eV) is smaller than that of PM6:PY-BO (0.556 eV), well supporting the greater V_{OC} of PM6:4Y-BO solar cells." ***(Page 13, revised manuscript)***.

50. Gong Y., *et al.* C-shaped ortho-benzodipyrrole-based acceptors with different electronic effects of top substituents for as-cast green-solvent processed high-performance organic solar cells. *Energy Environ. Sci.* **17**, 6844-6855 (2024) ***(Page 26, revised manuscript)***

51. Qian D., *et al.* Design rules for minimizing voltage losses in high-efficiency organic solar cells. *Nat. Mater.* **17**, 703-709 (2018).***(Page 26, revised manuscript)***

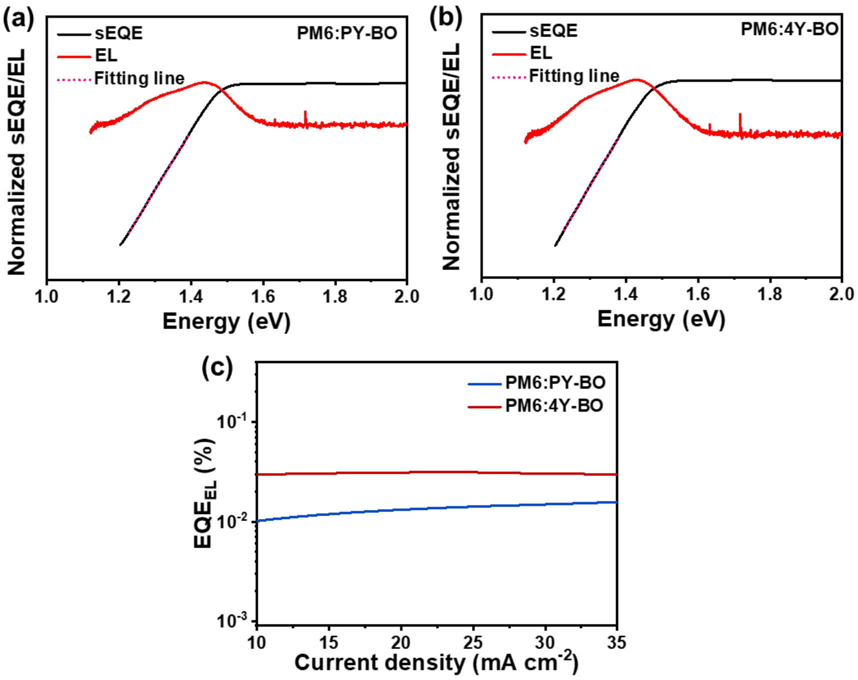

Fig. R3 (a-b) Normalized sEQE and EL spectra, and (c) electroluminescence quantum efficiency (EQE_{EL}) of the PM6:PY-BO and PM6:4Y-BO-based devices.

Table R2. Key parameters for energy loss of the PM6:PY-BO and PM6:4Y-BO-based devices.

Active layer	Eg (eV)	V_{OC} (V)	E_{loss} (eV)	V_{OC}^{SQ} (V)	ΔE_1 (eV)	ΔE_2 (eV)	ΔE_3 (eV)	EQE_{EL} (%)
PM6:PY-BO	1.485	0.929	0.556	1.214	0.271	0.058	0.227	1.5×10^{-2}
PM6:4Y-BO	1.478	0.944	0.534	1.208	0.270	0.057	0.207	3.2×10^{-2}

Q5. The literature has reported that the efficiency of PY-BO-based device has surpassed 19%, much higher than the 15.66% efficiency reported in this work. The authors should explain the reason.

Response: Thank you for your comment. In order to further assess the level of data we measured, we urgently and carefully searched for literatures about PY-BO based solar cells. With all due respect, but we have not found any photovoltaic efficiencies exceeding 19% or even 17% based on PY-BO acceptor. The following two references (*Adv. Mater.* **34**, 2110155 (2022) (PCE of 15.3%); *Adv. Funct. Mater.* **33**, 2215095 (2023) (PCE of 16.53%)) are the ones we searched based on PY-BO (PY-OD), and the polymer performance we recorded are at the same level in previous literatures.

Although several articles have reported that the finely optimized efficiency of all polymer OSCs can

reach 19%, the significant distinctions of side chains and/or skeletons between the employed polymer acceptors and PY-BO, and the device technology cannot be ignored. In addition, significant batch-to-batch differences of polymer acceptors is another critical factor and constraint on the device performance and application. Promisingly, as we reported in this manuscript, the high-performance tetramer 4Y-BO with partial polymer-like properties and defined chemical structure has greater potential to fabricate highly efficient and long-term stable OSCs.

Q6. The author states that the tetramer 4Y-BO was obtained with a significant yield of 80%. To enhance the credibility, please provide supporting data, such as yield results obtained from HPLC or LC-MS analysis.

Response: Thanks for your concern. In this study, the yield of 4Y-BO was calculated based on the mole amount of compound **2** instead of compound **4**. In order to obtain sufficient 4Y-BO, we have repeated several times of the synthetic procedure, and comparable results were obtained. As depicted in **Fig. R4**, the starting material of compound **2** (0.027 mmol feedstock) completely disappeared after the reaction, while a portion of compound **4** (3 equivalents of feed ratio of compound **2**) remained. After silica gel column chromatography, 4Y-BO was obtained by 158±10 mg (0.02~0.023 mmol), which corresponds to a molar yield of about 79±5% based on three repeat synthesis.

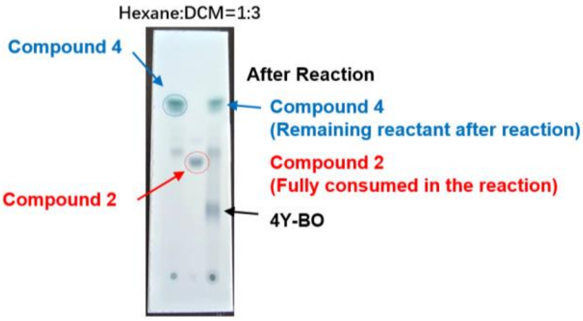

Fig. R4 Thin layer chromatography (TLC) of 4Y-BO.

Q7. The article by Li et al. (Nat Commun 14, 7996 (2023).) shows when under the continuous thermal annealing at 90 °C, the T90 based on their tetramer acceptor OSC was just over ten hours, while in this manuscript the T80 based on the PM6:4Y-BO OSC was 2125h. How does the author comment on this difference?

Response: Thank you very much for sharing this detail. In our study, the thermal stability was performed under 80 °C in a nitrogen filled glove box, which possesses oxygen content below 0.1 ppm,

and water content below 0.1 ppm. The device was placed under dark to exclude the light factor, and the solar simulator was only turned on when measuring the *J-V* plots. Every data point was averaged from at least five independent devices (***Page S4, revised Supplementary Information***). However, in the suggested report (*Nat Commun* **14**, 7996 (2023)), we failed to find the details about thermal stability measurements. Therefore, in addition to the different annealing temperatures (80 ºC *vs* 90 ºC), other uncertain measurement distinctions between our study and the literature may result in differences of device stability. In spite of the different device architectures, satisfactory long-term thermal stabilities of high molecular-weight oligomeric acceptors-based OSCs have been widely proven, such as *Angew. Chem. Int. Ed.* **62**, e202302888 (2023) (85 °C, T80=22 days); *Adv. Sci.* **11**, 2406772 (2024) (65 °C, T75=28 days); *Joule*, **7**, 2386-2401 (2023) (80 °C, T90=4500h); *Adv. Energy Mater.* 13, 2300301 (2023) (65 °C, T80=350 h) and so on.

Nevertheless, we would like to sharing some other opinions. As well-acknowledged, the thermal stability of OSCs is closely correlated with the device structure, hole/electron transport layers, and heterojunction morphology robustness, of which the latter is related to the distributions of metastable regions and defects. For bulk-heterojunction blend, robust morphology should correspond to more orderly and compact molecular packing in pure phases, and stronger heterojunction interfaces. Thus, we compared the aggregation behaviors of QY in literature and 4Y-BO in this study. As **Table. R3** suggests, compared to QY, 4Y-BO has smaller packing distance and much greater CCL, indicating greatly improved molecular ordering and thus reduced amorphous structures.

Table R1. Molecular packing parameters for neat films derived from GIWAXS fitting.

Acceptor	*d*-spacing (010) (Å)	CCL (010) (Å)	Ref.
QY	3.832	20.86	*Nat. Commun.* **14**, 7996 (2023)
4Y-BO	3.82	24.35	Our work

Furthermore, we conducted density functional theory calculations to study the A/A (between acceptor and acceptor) interactions and D/A (between donor and acceptor) interactions of PM6/4Y-BO and PM6/QY pairs. To simplify the calculation, we extracted the repeating unit of PM6 and single Y units of 4Y-BO or QY (with Cl or H end groups) to perform the simulations. As shown in **Fig. R5**, it can be seen that 4Y-BO exhibits stronger A/A interactions (-39.92 kcal/mole *vs*. -35.50 kcal/mole) than QY, which suggest the greater crystallinity habit of 4Y-BO and is well consistent with the data obtained

from GIWAXS (**Table. R3**). In addition, we can find that the D/A interaction between PM6 and 4Y-BO (-57.50 kcal/mole) is also stronger than that between PM6 and QY (-55.10 kcal/mole), which enhances the heterojunction interfaces, reduces the molecular peristalsis below *Tg*, and thereby achieves higher thermal stability and mechanical stability as we have disclosed.

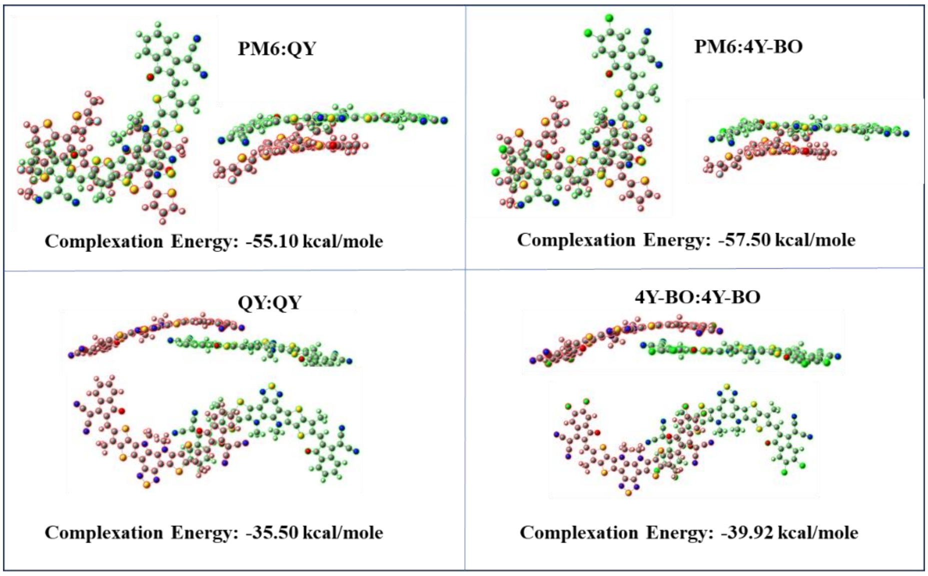

Fig. R5 Optimized intermolecular packing diagrams by density functional theory simulation.

Q8. Most literature report that the Tgs of trimers falls within the range of 150-180 °C (Adv. Funct. Mater. 2024, 2410092; Energy Environ. Sci., 2023,16, 3339; Nat Energy 2022, 7, 1180) Why are the Tg values for 4Y-BO and PY-BO mentioned in the main text significantly lower?

Response: Thanks for your comment and concern. In order to assess the level of data we measured, we carefully reviewed the recent articles on trimer acceptors. Compared to the suggested literature, significant differences of Tg of trimers can be found from different literatures, such as *Joule* **7**, (2023) 2386-2401 (Tg of trimer is 124 ºC); *Adv. Energy Mater.* **14**, 2303872 (2024) (Tg of trimer TYT-S is 131 ºC). *Angew. Chem. Int. Ed.* **63**, e202415141 (2024) (Tg of trimer 3BTT6Cl is 128 ºC). Therefore, we believe the Tg in our manuscript is reasonable. Moreover, considering the complicated influence of various external factors on Tg testing, it is recommended that the comparisons of different materials should be conducted under the identical preparation conditions and testing environment to improve reliability. Nevertheless, we have further repeated the Tg measurements for 4Y-BO and PY-BO films. As provided below in **Fig. R6**, the fitted Tg of 4Y-BO and PY-BO are determined to be 139 ºC and 127 ºC, respectively, both of which are comparable with our reported values (134 ºC, 123 ºC) and

further confirm the data reliability.

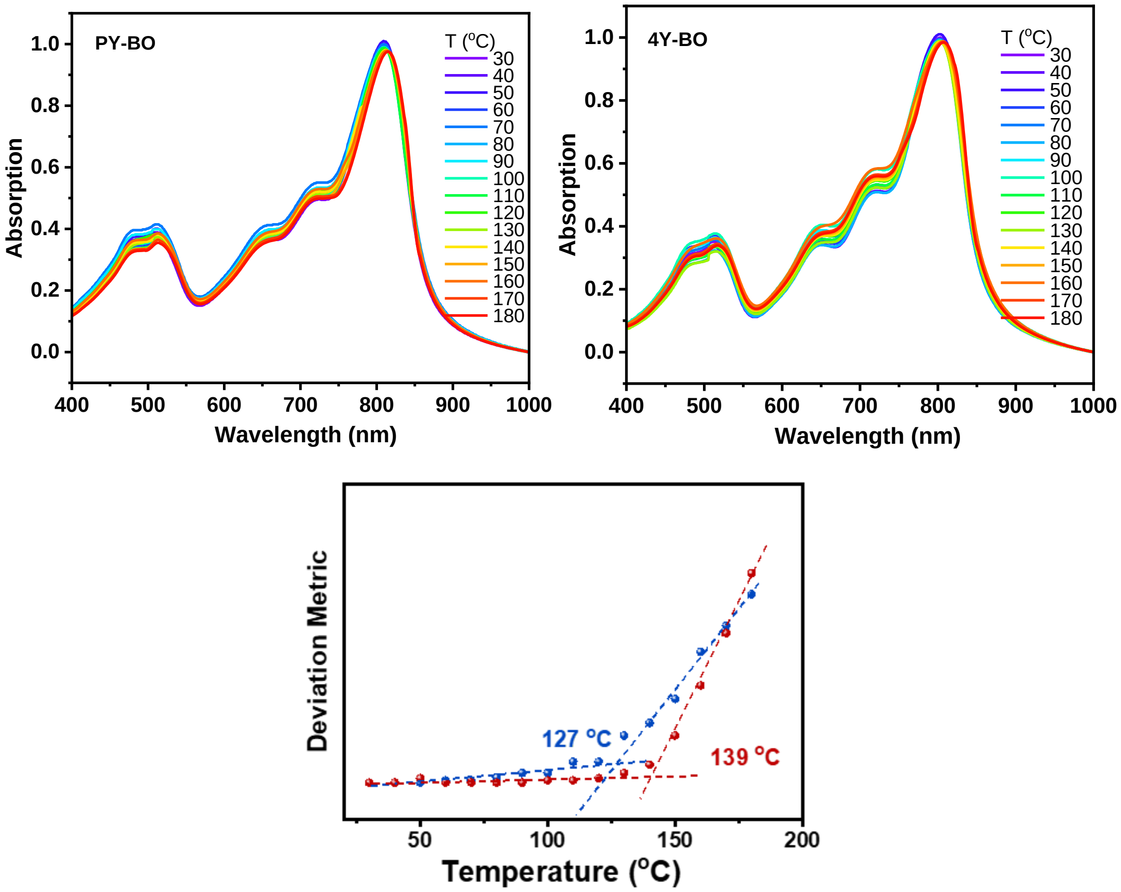

Fig. R6 Remeasured time-depended absorptions and deviation metric (DMT) plots of PY-BO and 4Y-BO neat films as a function of annealing temperature.

THE REVIEWER #2'S REPORT

= = = = = = = = = = = = = = = = = = =

Wang et al reported a polymer tetramer acceptor, applying the corresponding polymer as a control, they deeply investigated the advantages of the tetramer with various characterizations, and also reported an excellent efficiency of 19.75%. However, there are still the following questions that should be clearly explained before acceptance:

Response: We deeply thank you for recognizing the significance of our work and recommending it for publication. We are also very grateful for your insightful comments and constructive suggestions, which have helped us to improve this manuscript. In the following, we provide detailed responses to the comments and suggestions point-by-point.

Q1. As in the paper, the MW of polymer acceptor is quite low (almost 4 monomers), and hence, the comparison between polymer and tetramer in this paper is indeed a combination of purity (or molecular distribution) in aggregation and fluorination in structure. So, in your opinion, what would the comparison happen, especially for the stability and mechanical flexibility between the tetramer and a higher MW (e.g. > 20K)

Response: Thank you very much for sharing your opinion and your kind comment. As intensively reported, oligomers with larger molecular-weights usually have higher Tg, which is beneficial for stabilizing the blend morphology and achieving good device stability. Meanwhile, oligomers with defined chemical structures and ordered molecular packing can help reduce the metastable region and defects, and thus improve the morphology robust and device stability. Therefore, as we disclosed in this study, tetramer with significant molecular-weight and appropriate molecular aggregation is one promising candidate for stable organic solar cells. On the contrary, polymer acceptors with low MW may suffer from limited molecular ordering and significant metastable aggregation structures due to the disturbance of small molecular fractions. Assuming that the MW of polymer acceptors (or oligomers) significantly raise, the interchain entanglement can be further enhanced, which could improve the mechanical flexibility of active layers. Besides, greater Tg can be expected along with the increase of MW. Based on these considerations, it seems that both the thermal stability and mechanical flexibility solar cells can be concurrently improved for high MW polymer or oligomer acceptor-based OSCs. However, we should note that along with the increase of MW, the solubility of resultant acceptor

may decrease, negatively impacting miscibility and nanoscale phase-separations. At the same time, severe molecular entanglement may restrict the molecular stacking ordering, thereby adversely affecting device performance. Therefore, no matter oligomers or polymers, we think the MW should be rationally controlled for balancing the performance and long-term stability.

Q2. For the absorption spectrum, compared the polymers (ca. four acceptors) with tetramer, why would polymers result in serious interchain entanglements or serious pre-aggregation, but tetramer could lead to a uniform distribution in the dilute solution? Is it just because of their different aggregation (such H and J)? The reasons for the redshifts of the polymer's spectrum in solution or different redshifts from solution to films should be investigated more clearly.

Responses: Thank you for your critical comment. From **Fig. 2a-c** ***(Page 9, revised manuscript)***, we can discover that the absorptions of PY-BO and 4Y-BO in respective solutions maintained clear (0-0) peaks even at 120 °C, indicating strong aggregation characteristics for both the tetramer and polymer. However, due to the uneven MW distribution of the polymer and the presence of larger molecular-weights (average 4 repeated units, but have 5 or even more repeated units, **Fig. R7**) fractions, the polymer could exhibit more notable entanglement ability and pre-aggregation property, and result in less significant changes in the absorptions of PY-BO. On the contrary, the (0-0) absorption peak of 4Y-BO shows a more significant variation, indicating a moderate pre-aggregation. In addition, during the film-forming process from solution to film state, the intermolecular spacing decreases. Under this circumstance, the intermolecular interactions can have a more notable impact on film formation and crystallinity. The GIWAXS studies support the better crystallinity of 4Y-BO than that of PY-BO (**Fig. 2g-2i**, ***Page 9, revised manuscript***) and thus greater intermolecular interactions.

To further clarify this point, we have supplemented the density functional theory calculations on the interactions of the polymer and tetramer, with one single Y unit of 4Y-BO or PY-BO (with Cl or H end groups) employed for simplification. As depicted in **Fig. R7** below, the complexation energy between 4Y-BO and 4Y-BO (-39.92 kcal mol^{-1}) is notably higher than that between PY-BO and PY-BO (-35.50 kcal mol^{-1}), which confirms the stronger interactions for 4Y-BO and thus can provide greater driving force for molecule self-assembly and thus larger absorption redshift.

Additionally, we have incorporated some discussions into the revised manuscript as "During the film-forming process from solution to film state, the intermolecular spacing decreases. Under this

circumstance, intermolecular interactions can have a dominating impact on film formation and crystallinity. To further clarify this point, we calculated the intermolecular interactions for the polymer and tetramer. As depicted in Supplementary Fig. 13, the complexation energy between 4Y-BO (-39.92 kcal mol^{-1}) and 4Y-BO is notably higher than that between PY-BO and PY-BO (-35.50 kcal mol^{-1}), which could provide greater driving force for molecule self-assembly and thus larger absorption redshift." ***(Page 10, revised manuscript)***

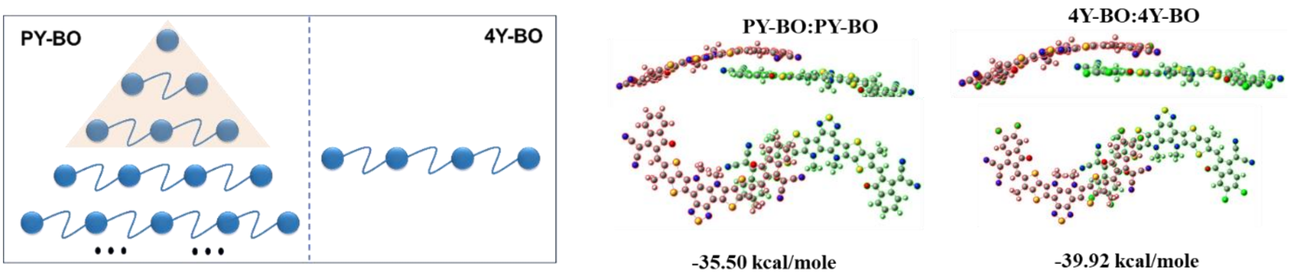

Fig. R7 Schematic diagram of molecular distribution and optimized intermolecular packing diagrams by density functional theory simulation.

Q3. In the stability part, both of the Tg exceed much above the thermal test temperature. What's your opinion on the much better thermal stability of tetramer blends, especially its much -lower burn-in loss?

Response: Thanks for your critical comment. As we know, during the film-forming process of bulk heterojunction blends, metastable aggregation phase is inevitably generated, which would move to thermodynamic steady state during the device operation. The evolution of molecular aggregation could result in degradation of blend morphology and disrupt the original charge transport pathways. As for the polymer acceptor PY-BO with limited MW, the presence of low MW polymer fractions increases the possibility of molecular peristalsis at temperatures below Tg, which can be indicated from the trend of Tg testing (**Fig. R8**, marked in red circle). In comparison, the tetramer 4Y-BO with unified and high molecular weight can effectively inhibit the molecular motion (below Tg) and thus reduce the burn-in loss during thermal stability measurements.

Based on your enlightenment and the above considerations, we have incorporated some relevant discussions into the revised manuscript as "It is worth noting that within the range of 75 °C to T_g, PY-BO film exhibits more significant state changes, which may be due to the aggregation variations caused by the peristaltic behavior of low molecular-weight fragments. With a uniform and high molecular

weight, the tetramer 4Y-BO effectively inhibited molecular motion (below T_g) and thus reduce the burn-in loss." ***(Pages 19, revised manuscript)***

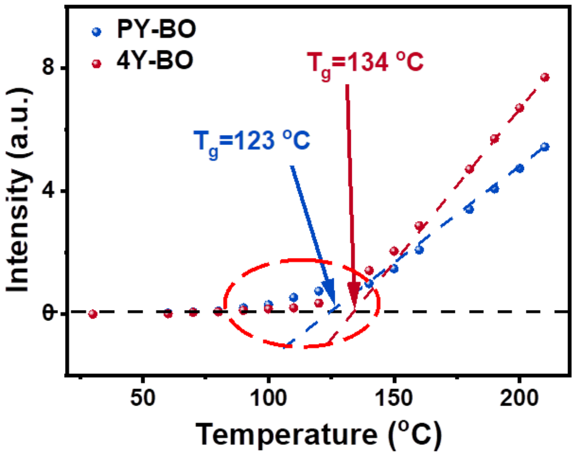

Fig. R8 Deviation metric (DMT) plots of PY-BO and 4Y-BO neat films as a function of annealing temperature.

Q4. In small molecules, fluorinating the end-capped group normally leads to a larger non-radiative energy loss, but the fluorinated tetramer could obtain a reduced non-radiative energy loss in this study. What's your explanation?

Response: Thanks for your critical comment and noticing this detail. As well-acknowledged, the non-radiative energy loss inside OSCs is controlled by complicated factors, such as chemical structures, morphology textures, domain purity and crystallinity, as well as donor/acceptor interfaces. Fluorinating the end-capped of acceptors usually afford deeper LUMO energy levels, which would narrow the energy gap between HOMO level of the donor and LUMO level of the acceptor for decreased open-circuit voltages.

To further clarify this point, we investigated the energy loss (E_{loss}) of the two optimal solar cells. The highly-sensitive external quantum efficiency (sEQE) and electroluminescence (EL) spectra were measured as depicted in **Fig. R9** below, with detailed parameters collected in **Table R3** below. The E_gs of PM6:PY-BO and PM6:4Y-BO based devices are determined to be 1.485 eV and 1.478 eV, respectively. According to well-documented methods in the literature (*Energy Environ. Sci.* **17**, 6844-6855 (2024)), the E_{loss} can be described as three parts based on the detailed balance theory: E_{loss}=ΔE_1 +ΔE_2+ΔE_{nr}, where ΔE_1 is constrained by the Shockley-Queisser limit, ΔE_2 is the inevitable radiative energy loss, and ΔE_3 is the non-radiative energy loss. Based on the measurements, the ΔE_1 of PM6:PY-

BO and PM6:4Y-BO-based devices are 0.271 and 0.270 eV, and ΔE_2 are 0.058 and 0.057 eV, respectively. However, as shown in **Fig. R9c**, the EQE_{EL} value of PM6:4Y-BO processed device is 3.2×10^{-2}, much higher than that of the PM6:PY-BO device (1.5×10^{-2}), corresponding to a decreased ΔE_{nr}. Obtained from EQE_{EL} measurements, ΔE_{nr} values are markedly different with 0.227 and 0.207 eV for PM6:PY-BO and PM6:4Y-BO-based devices, respectively.

In addition, to further expound the reduced non-radiative energy loss of PM6:4Y-BO device, we measured the photoluminescence quantum yield (PLQY) of PY-BO and 4Y-BO neat films. As shown in **Fig. R10**, the PLQY of PY-BO and 4Y-BO are calculated to be 8.26% and 1.53%, respectively. As previously reported (*Nat. Mater.* **17**, 703–709 (2018)), the significantly increased PLQY of 4Y-BO should be one important factor for the reduction of non-radiative voltage loss. Besides, the lower ΔE_{nr} of PM6:4Y-BO device should be partly attributed to the more ordered molecular stacking and appropriate phase-separations, which promote charge transfer and separation, reduces charge accumulation and recombination at the interface. As a result, the overall E_{loss} of PM6:4Y-BO device (0.534 eV) is smaller than that of PM6:PY-BO (0.556 eV), well supporting the greater V_{OC} of PM6:4Y-BO solar cells.

Based on the supplementary measurements and analysis, we have incorporated some discussion into the revised manuscript as "In addition, we highlight the greater V_{OC} of PM6:4Y-BO device in spite of the slightly deeper LUMO level of 4Y-BO (Table 1). In order to clarify this point, we move to explore the energy loss (E_{loss}) of solar cells. Highly-sensitive external quantum efficiency (sEQE) and electroluminescence (EL) spectra were measured as depicted in Fig. 3d-3f, with detailed parameters collected in Supplementary Table 8. The E_gs of PM6:PY-BO and PM6:4Y-BO devices are determined to be 1.485 eV and 1.478 eV, respectively. The E_{loss} can be described as three parts based on the detailed balance theory: $E_{loss}=\Delta E_1+\Delta E_2+\Delta E_{nr}$, where ΔE_1 is constrained by the Shockley-Queisser limit, ΔE_2 is the inevitable radiative energy loss, and ΔE_3 is the non-radiative energy loss.[50] In this scenario, the ΔE_1 of PM6:PY-BO and PM6:4Y-BO-based devices are 0.271 and 0.270 eV, and ΔE_2 are 0.058 and 0.057 eV, respectively. It is worth noting that the ΔE_{nr} of the two solar cells, obtained from EQE_{EL} spectra, are markedly different with 0.227 and 0.207 eV for PM6:PY-BO and PM6:4Y-BO devices, respectively. In order to further expound the reduced non-radiative energy loss of PM6:4Y-BO device, we measured the photoluminescence quantum yield (PLQY) of PY-BO and 4Y-BO neat films. As shown in Supplementary Fig. 17, the PLQY of PY-BO and 4Y-BO are determined to be 8.26% and

1.53%, respectively. As previously reported,[51] the increased PLQY of 4Y-BO should be one important factor for the reduction of ΔE_{nr}. Besides, the lower ΔE_{nr} of PM6:4Y-BO device should be partly attributed to the more ordered molecular stacking and appropriate phase-separations, which promote charge transfer and separation, reduce charge accumulation and recombination at the interfaces. As a result, the overall E_{loss} of PM6:4Y-BO device (0.534 eV) is smaller than that of PM6:PY-BO (0.556 eV), well supporting the greater V_{OC} of PM6:4Y-BO solar cells." ***(Page 13, revised manuscript)***

50. Gong Y., *et al.* C-shaped ortho-benzodipyrrole-based acceptors with different electronic effects of top substituents for as-cast green-solvent processed high-performance organic solar cells. *Energy Environ. Sci.* **17**, 6844-6855 (2024). ***(Page 26, revised manuscript)***

51. Qian D., *et al.* Design rules for minimizing voltage losses in high-efficiency organic solar cells. *Nat. Mater.* **17**, 703-709 (2018). ***(Page 26, revised manuscript)***

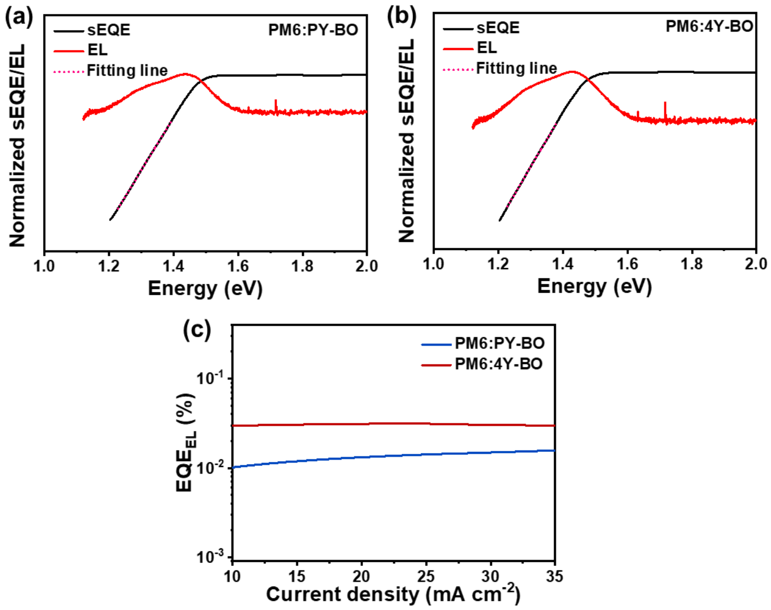

Fig. R9 (a-b) Normalized sEQE and EL spectra, and (c) the electroluminescence quantum efficiency (EQE_{EL}) of the PM6:PY-BO and PM6:4Y-BO-based devices.

Table R3. Key parameters for energy loss of the PM6:PY-BO and PM6:4Y-BO-based devices.

**Active layer**	***Eg* (eV)**	**V_{OC} (V)**	**E_{loss} (eV)**	**V_{OC}^{SQ} (V)**	**ΔE_1 (eV)**	**ΔE_2 (eV)**	**ΔE_3 (eV)**	**EQE_{EL} (%)**
**PM6:PY-BO**	1.485	0.929	0.556	1.214	0.271	0.058	0.227	1.5×10^{-2}
**PM6:4Y-BO**	1.478	0.944	0.534	1.208	0.270	0.057	0.207	3.2×10^{-2}

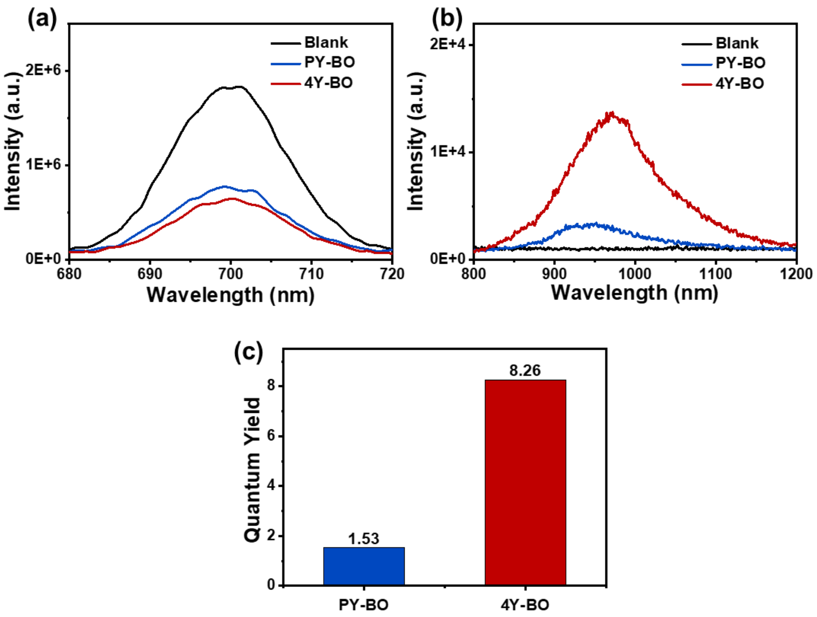

Fig. R10 (a-b) Excitation and emission spectra of blank sample, PY-BO and 4Y-BO, (c) Quantum Yield of PY-BO and 4Y-BO.

Based on the above supplementary studies, we have incorporated the **Fig. R9** into the revised manuscript as **Figs. 3d-3f** ***(Page 11, revised manuscript)***. **Table R3** was collected in **Supplementary Table 8** ***(Page S24).*** **Fig. R10** has been collected in the revised Supplementary Information as **Supplementary Fig. 17**. (***Page S14***)

THE REVIEWER #3'S REPORT

= = = = = = = = = = = = = = = = = = =

Indefinite structure and excessive entanglement of polymers result in large batch differences and limited photovoltaic performance of all-polymer solar cells (all-PSCs), which is a long-term challenge restricting its industrialization. Developing high molecular-weight giant molecule acceptors with definite chemical structures would be a potential alternative approach. In this manuscript, the authors reported a linear giant molecule acceptor 4Y-BO for constructing efficient and long-term stable organic solar cells (OSCs). A control polymeric acceptor namely PY-BO carrying identical main backbone and side groups, is synthesized in order to reach a comprehensive comparison in term of molecular aggregation, photovoltaic properties, as well as device thermal/mechanical stabilities. Based on measurements and analysis, the author disclosed that 4Y-BO based OSCs have achieved higher PCE of up to 19.75% with more preferred narrow batch-to-batch variations than the polymer PY-BO based device. In addition, the 4Y-BO exhibits high thermal and mechanical stability of corresponding devices. This work comprehensively compared the performance differences between the giant molecule acceptor and polymer acceptor with comparable molecular weights, fully affirming the advantages and enormous development potential of high molecular-weight giant molecule acceptors. Therefore, I recommend the acceptance of this manuscript for the publication in Nature Communications after some minor revisions addressing following technique concerns and comments.

Response: We deeply thank you for the highly positive comments about the significance of our manuscript and recommending it for publication in ***Nature Communications***. We are also very grateful for your insightful comments and constructive suggestions, which have helped us to improve this manuscript. In the following, we provide detailed responses to the comments and suggestions point-by-point.

Q1. Fig. S8 shows that the HOMO and LUMO orbitals of the 4Y-BO are distributed on different units with less overlap and coupling, which typically gave small extinction coefficient for absorption. In addition, the HOMO electron cloud density of PY-BO is located on one Y nucleus, while the HOMO of 4Y-BO is located on 2Y cores. Please further confirm the simulation results and provide some possible explanations.

Response: Thanks for your concerns. According to your kind suggestion, we further investigated the excitation properties of PY-BO and 4Y-BO based on CAM-B3LYP/6-311g(d,p)/polarizable continuum model method. As shown in **Fig. R11** below, both the absorption spectra of PY-BO and 4Y-BO are dominated by S0→S1 transition. The oscillator strength (*f*) of S0→S1 transition for PY-BO and 4Y-BO are 7.7890 and 8.8248, respectively, which indicate better light absorption of 4Y-BO than that of PY-BO. Sr (ranges from 0 to 1) represents the overlap between the electron and hole, and the Sr closer to 1 indicates greater overlap. As the results confirmed, the Sr of S0→S1 transition for 4Y-BO is slightly higher than that of PY-BO, and both of them are close to 0.75. The large overlap means there are high transition density between the involved molecular orbitals, thus the large transition oscillator strength. Therefore, the tetramer should have excellent light absorption ability, and consistent well with our experimental results (**Fig. 1c**, revised manuscript).

To analyze the essential characteristics of electronic transitions, we further examined the contribution of each pair of molecular orbital transitions to S0→S1 transition (**Fig. R12 and Table R4**). Results show that all the four occupied frontier orbital (H-3, H-2, H-1, H) and the four unoccupied frontier orbital (L, L+1, L+2, L+3) contribute to the electron transition from S0 to S1 state, and the contribution ranges from 2 to 20%. The total contribution accounts for over 70%. The detailed frontier orbital distribution was further investigated. As shown in **Table R5**, the tetramer contains four occupied frontier orbitals with similar energy, distributed on different Y-units. Similarly, four unoccupied frontier orbitals with similar energy are distributed on different units. The molecular orbital distribution shows that the frontier orbitals of the monomer mix with each other to form the new molecular orbitals following the monomer forms a tetramer.

Fig. R11 and **Fig. R12** have been collected into the revised Supplementary Information as **Supplementary Fig. 9** and **Supplementary Fig. 10,** respectively **(*Pages S10-S11*). Table R4** and **Table R5** have been collected into the revised Supplementary Information as **Supplementary Table 2** and **Supplementary Table 1,** respectively **(*Page S21-S22*).**

Based on the supplementary measurements and analysis, we have incorporated some discussion into the revised manuscript as "Subsequently, we used density functional theory to calculate the orbital distribution as shown in Supplementary Table 1. The tetramer contains four occupied frontier orbitals with similar energy, distributed on different Y-units. Similarly, four unoccupied frontier orbitals with similar energy are distributed on different repeat units. The molecular orbital distribution shows that

the frontier orbitals of the monomer mix with each other to form the new molecular orbitals following the formation of tetramer, which shows a consistent trend with the experimental results." ***(Page 7, revised manuscript)***

and "We further investigated the excitation properties of PY-BO and 4Y-BO based on CAM-B3LYP/6-311g(d,p)/polarizable continuum model method. As shown in Supplementary Fig. 9, both the absorption spectra of PY-BO and 4Y-BO are dominated by S0→S1 transition. The oscillator strength (*f*) of S0→S1 transition for PY-BO and 4Y-BO are 7.7890 and 8.8248, respectively, which indicates better light absorption of 4Y-BO than that of PY-BO. To analyze the essential characteristics of electronic transitions, we further examined the contribution of each pair of molecular orbital transitions to S0→S1 transition (Supplementary Fig. 10 and Supplementary Table 2). Results show that all the four occupied frontier orbital (H-3, H-2, H-1, H) and the four unoccupied frontier orbital (L, L+1, L+2, L+3) contribute to the electron transition from S0 to S1 state, and the contribution ranges from 2 to 20%. The total contribution accounts for over 70%. The detailed frontier orbital distribution was further investigated. Sr (ranges from 0 to 1) represents the overlap between the electron and hole, and the Sr closer to 1 indicates the greater overlap. As the results confirmed, the Sr of S0→S1 transition for 4Y-BO is slightly higher than that of PY-BO, and both of them are close to 0.75. The large overlap means high transition density between the involved molecular orbitals, thus the large transition oscillator strength. Therefore, the tetramer should have excellent light absorption ability, and consistent well with our experimental results." ***(Pages 7-8, revised manuscript)***

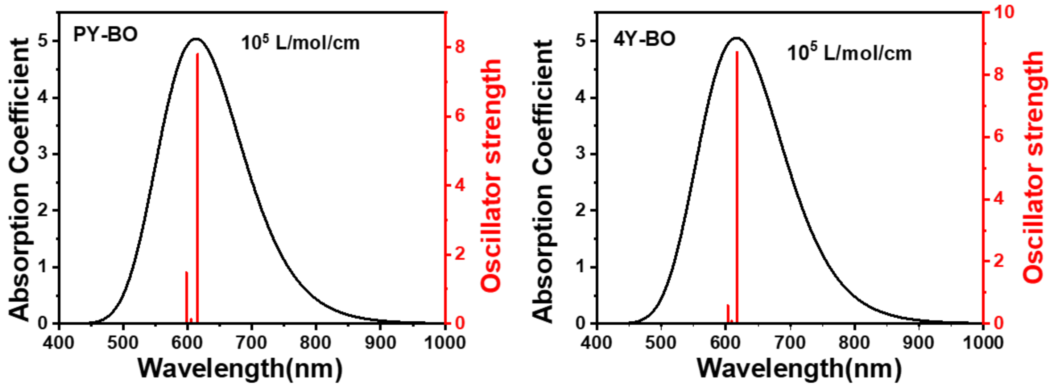

Fig. R11. Simulated absorption spectra of PY-BO and 4Y-BO in dibutylether solution (ε=3.0).

Table R4. The electronic transition energy E, major orbital transition contribution, Oscillator strength (f), and Sr index for S0→S1 transition calculated.

Systems	Excited State	E (eV)	Orbital transition	Orbital transition contribution (%)	*f*	Sr (a.u.)
PY-BO	S0→S1	2.0127	H-1 → L	19.2076	7.7890	0.7431
			H-2 → L+1	9.4987		
			H-3 → L+2	9.1002		
			H-3 → L+3	8.0923		
			H-2 → L	4.9524		
			H-1 → L+1	4.7002		
			H-3 → L	4.0516		
			H → L+1	3.2737		
			H-1 → L+3	3.1536		
			H-2 → L+3	2.2663		
4Y-BO	S0→S1	2.0083	H → L+1	12.9215	8.7248	0.7445
			H-1 → L+3	9.1199		
			H-1 → L+2	7.8900		
			H-1 → L	7.8456		
			H-3 → L	7.5420		
			H-2 → L+3	4.7629		
			H → L	4.5030		
			H → L+3	3.5511		
			H-2 → L+2	3.5107		
			H-3 → L+2	3.3354		
			H → L+2	2.8494		
			H-2 → L+1	2.4152		

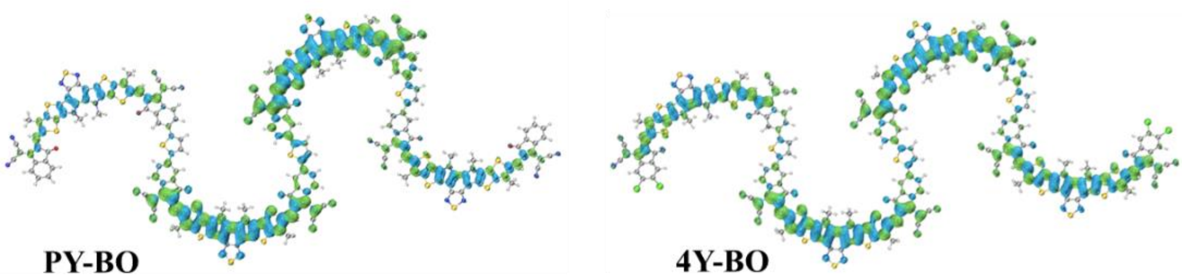

Fig. R12. Electron and hole distributions of S0→S1 for the studied systems level. The green and blue contours correspond to the distribution of electron and hole, respectively.

Table R5. The frontier molecular orbitals distribution for PY-BO and 4Y-BO.

	PY-BO	4Y-BO

L+3	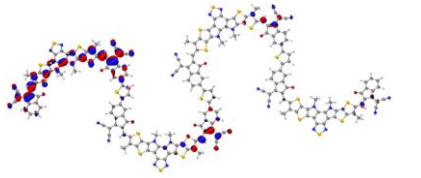 -3.57 eV	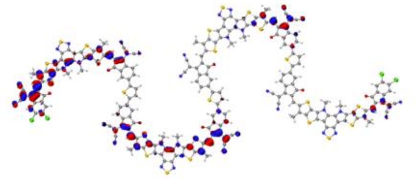 -3.63 eV
L+2	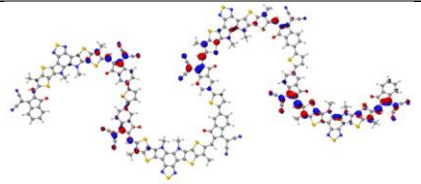 -3.64 eV	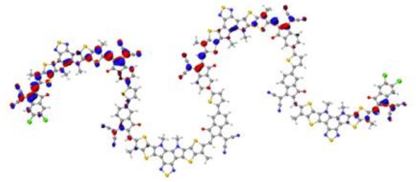 -3.68 eV
L+1	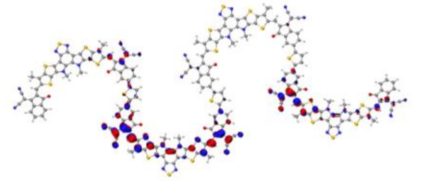 -3.69 eV	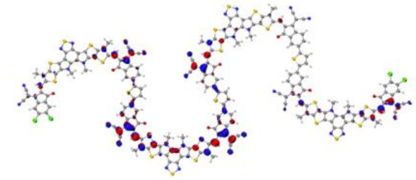 -3.72 eV
L	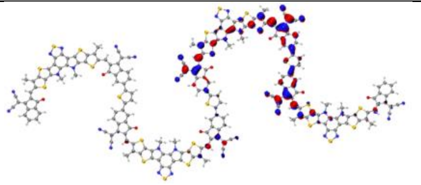 -3.74 eV	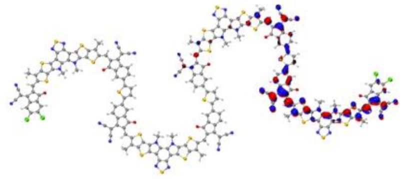 -3.77 eV
H	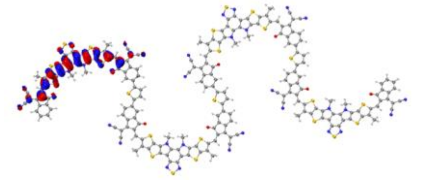 -5.69 eV	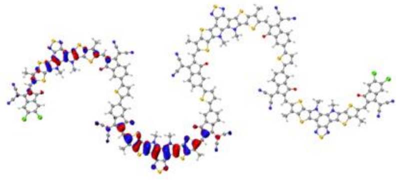 -5.66 eV
H-1	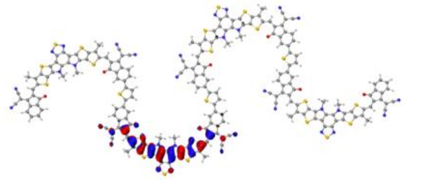 -5.68 eV	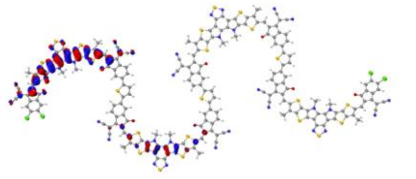 -5.68 eV
H-2	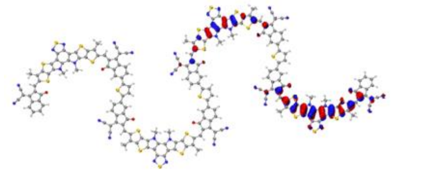 -5.66 eV	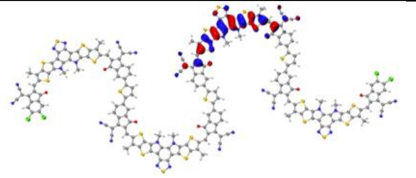 -5.69 eV
H-3	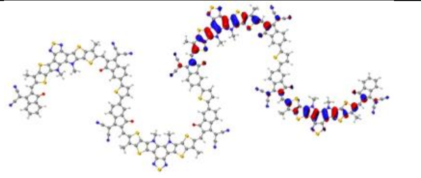 -5.60 eV	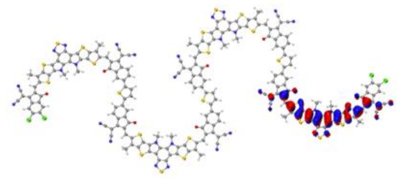 -5.75 eV

Q2. The authors should include color bars in Fig. S4-S6 indicating the negative and positive values,

and provide a detailed description of the methods and boundary conditions used for density functional theory simulations.

Response: Thank you for your kind suggestion. The color bars have been added in **Fig. S4-S6** indicating negative and positive values. (***Pages S9, Supplementary Information***)

Besides, the methods and boundary conditions used for density functional theory simulations are also described in detail in the revised Supplementary Information. (***Pages S3, Supplementary Information***)

Q3. In order to more intuitively and clearly compare the intermolecular differences between the donor and the giant molecule acceptor or polymer acceptor in the blend film, it will be very convincing to perform density functional theory simulations on them and incorporate corresponding discussions about the results.

Response: Thanks very much for your critical comment. According to your kind suggestion, we have performed density functional theory calculations on the interactions between the donor PM6 and 4Y-BO or PY-BO (**Fig. R13**). To simplify the calculation, we extracted the repeating units of PM6 as donor calculation unit and a single Y unit of 4Y-BO or PY-BO (with Cl or H end groups) as acceptor calculation units. As shown in **Fig. R13,** the complexation energy between PM6 and giant tetramer 4Y-BO was significantly greater than that between PM6 and PY-BO (-57.50 kcal mol^{-1} *vs*. -55.10 kcal mol^{-1}). The stronger interactions between donor and 4Y-BO are beneficial for good compatibility and phase separation in heterojunctions, thereby achieving good charge generation. In addition, we also calculated the complexation energy inside 4Y-BO or 4Y-BO molecules. The result show that 4Y-BO exhibits much greater complexation energy of -39.92 kcal mol^{-1} compared to that of PY-BO (-35.50 kcal mol^{-1}). The stronger interaction interactions of 4Y-BO would beneficial for ordered molecular assembly in neat films (***Fig. 2i, Page 9, revised manuscript***) and acceptor phases of the blend film (***Fig. 5c, Page 16, revised manuscript***), and facilitate the charge transport. Besides, the enhanced intermolecular interactions can also be an important factor for the more competitive mechanical flexibility of PM6:4Y-BO solar cells (***Fig. 6c-6f, Page 19, revised manuscript***).

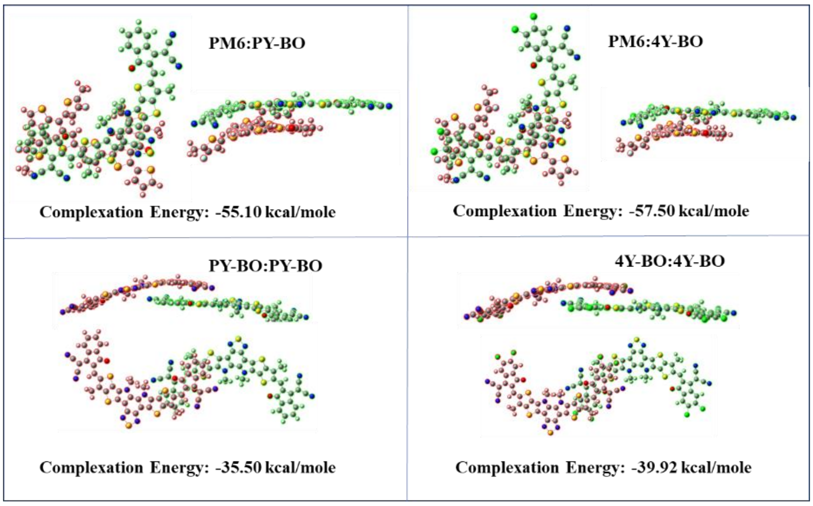

Fig. R13 Optimized intermolecular packing diagrams by density functional theory simulation at the lowest energy steady-state of different molecular pairs. Repeat unit of PM6 and a single Y-unit of 4Y-BO or PY-BO (with Cl or H endcaps) were employed to perform the simulations.

Based on your kind suggestion and the above results, we have incorporated some discussions into the revised manuscript as "In order to further understand the evolutions of molecular packing in blend films, we performed density functional theory (DFT) calculations on the intermolecular interactions of PM6/PY-BO and PM6/4Y-BO pairs. As shown in Supplementary Fig. 29, the PM6/4Y-BO pair shows a greater complexation energy of -57.50 kcal mol^{-1} than that of PM6/PY-BO (-55.10 kcal mol^{-1}), which should be beneficial for appropriate phase-separations and thus improved charge generation property. In addition, by combining the strong complexation energy within the 4Y-BO and 4Y-BO, the crystallinity of the donor and acceptor in blend film can be improved, which is consistent with the experimental results." (***Page 17, revised manuscript***)

and "The improved mechanical flexibility of PM6:4Y-BO should be partly attributed to the greater intermolecular interactions in acceptor phases and the stronger interfacial interactions between the donor and acceptor phases (Supplementary Fig. 13 and 29)" (***Page 19-20, revised manuscript***)

Additionally, the **Fig. R13** has been collected into the revised Supplementary Information as **Supplementary Fig. 13 and 29** (***Pages S12 and S18***)

Q4. The author compared the pre-aggregation characteristics of 4Y-BO and PY-BO by temperature-dependent absorption measurements, why the 4Y-BO shows mildly enlarged variations of wavelength and intensity, but the pre-aggregation characteristics reduced? Please provide some possible explanations.

Response: Thanks for your concern. Generally, the weaker the pre-aggregation of molecules in solution, the more likely they are to be de-aggregated by thermal stress during heating the solution, and thus more significant spectral changes (maximum wavelength and intensity) occur. On the contrary, when the molecules are pre-aggregated strongly in solution, they are more likely resistant to higher thermal stress and produce limited spectral evolutions. In this study, we can discover that during the temperature-dependent absorption measurements, the giant tetramer 4Y-BO receives mildly enlarged variations of wavelength and intensity than those of polymer PY-BO, corresponding to a slightly decreased pre-aggregation in dilute solution. In spite of the decent pre-aggregation characteristics of both 4Y-BO and PY-BO (0-0 peaks remain prominent even in hot solutions), the mildly stronger pre-aggregation of the latter one should be attributed to the stronger entanglements of the larger molecular-weight fractions. Based on this point, we have incorporated some discussion into the revised manuscript as "The slightly stronger pre-aggregation of PY-BO can be attributed to the excessive entanglement of larger molecular-weight components." ***(Page 8, revised manuscript)***

Q5. The authors emphasize that the PM6:4Y-BO blend film forms a more uniform crystalinity and clear fiber structure, which is one critical factor to its outstanding exciton/charge properties and photovoltaic performance. Supplementing the contact angles of the employed materials and Flory-Huggins interaction parameters should be helpful to provide an in-depth illustration about the morphology landscape.

Response: Thanks for your instructive comments. According to your kind suggestion, we have measured the contact angles of the employed materials and calculated the Flory-Huggins interaction parameters. As provided in **Fig. R14** and **Table R6** below, the surface energies (γ_s) of PM6, PY-BO, and 4Y-BO were determined to be 38.4, 45.8, and 44.3 mN m^{-1}, respectively. The Flory–Huggins interaction parameter (χ) can be calculated from the equation $\chi = K(\sqrt{\gamma_1} - \sqrt{\gamma_2})^2$. Therefore, the $\chi_{PM6/4Y\text{-}BO}$ (0.21 K) is smaller than that of $\chi_{PM6/4Y\text{-}BO}$ (0.33 K), suggesting improved compatibility between PM6 and 4Y-BO. Combined with the stronger intermolecular interactions between the donor and 4Y-BO (as **Fig. R13** discussed above), the PM6:4Y-BO blend can afford more uniform heterojunction textures and surface potential distributions (***Fig. 5d-5f, Page 16, revised manuscript***) and avoid the formation of excessive phase-separations.

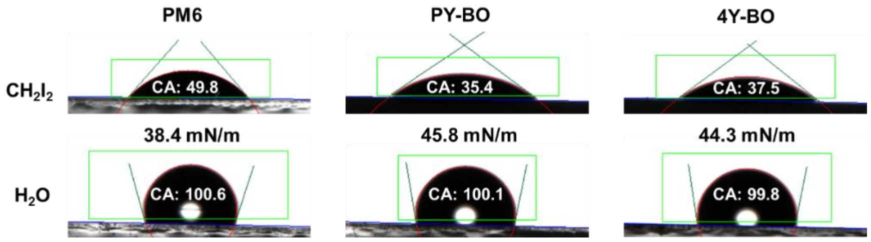

Fig. R14 Contact angles of water and diiodomethane droplets of neat and blend films.

Table R6 Contact angles and surface energies (γ_s) of the neat films, and the calculated Flory–Huggins interaction parameters (χ) between donor and acceptors.

	Contact angle (°)			
Film	H_2O	CH_2I_2	γ_s (mN m^{-1})	$\chi_{PM6/acceptor}$ (K)
PM6	100.6	49.8	38.4	-
PY-BO	100.1	35.4	45.8	0.33
4Y-BO	99.8	37.5	44.3	0.21

Based on the above supplementary measurements, we have incorporated some discussions into the revised manuscript as "Flory-Huggins interaction parameters (χ) are further calculated based on the surface energies (γ_s) of PM6, PY-BO, and 4Y-BO, obtained from contact angles measurements. As Supplementary Fig. 30 and Supplementary Table 11 suggest, the 4Y-BO receives improved compatibility with PM6 compared to that between PY-BO and PM6, which should be partly attributed to the greater intermolecular interactions of PM6/4Y-BO pair (Supplementary Fig. 29). The better compatibility between 4Y-BO and PM6 is beneficial for more uniform and appropriate heterojunction textures as disclosed above." (***Pages 17-18, revised manuscript***)

Additionally, the **Fig. R14** and **Table R6** have been incorporated into the revised Supplementary Information as **Supplementary Fig. 30** and **Supplementary Table 11**, respectively, (***Page S19 and S25***). The details of contact angle measurement have been collected in the revised Supplementary Information. (***Pages S2-S3***)

Q6. Please clarify if the external quantum efficiency (EQE) spectra align with the absorption spectra of the blend film, and provide the absorption spectra of the blend films.

Response: Thanks for your comment. As you kindly suggested, we have measured the absorption spectra and displayed the extinction coefficient spectra of PM6:PY-BO and PM6:4Y-BO blend films in **Fig. R15**. It is apparent that the PM6:4Y-BO blend film receives higher extinction coefficients at nearly all the wavelengths compared to that of PM6:PY-BO, particularly in the absorption range

assigned to acceptor. This improvement should be collectively afforded by the larger extinction coefficient of 4Y-BO than that of PY-BO, and the improved molecular ordering and crystallinity in blend film. Therefore, we can discover that the absorption profiles of blend films are globally consistent with the EQE responses of solar cells. Meanwhile, the differences between absorption and EQE spectra should be partly attributed to the distinct post-treatments of the active layers.

Based on your kind suggestion and the above results, we have incorporated some discussions into the revised manuscript as "External quantum efficiency (EQE) spectra (Fig. 3c) suggest a global enhancement of photon utilization in PM6:4Y-BO solar cell, well-supporting the more preferable J_{SC} compared to that of PM6:PY-BO, which is basically consistent with the absorption spectra of the blend films (Supplementary Fig. 16)." (***Page 12, revised manuscript***)

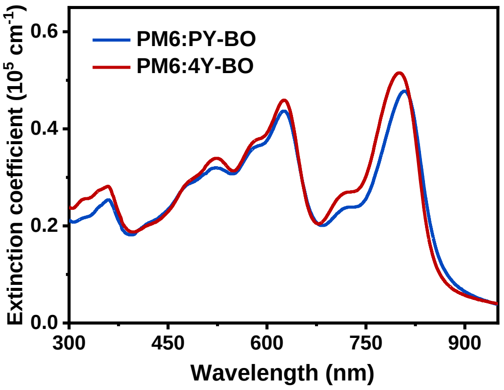

Fig. R15 The extinction coefficient spectra of PM6:PY-BO and PM6:4Y-BO neat blend films.

Q7. The solid and dashed lines in the line-cut profiles (Fig. 2i and 5c) should be clearly labeled for better comparisons.

Response: Thank you for your kind suggestion. The solid and dashed lines in the line-cut profiles (**Fig. 2i and 5c**) have been clearly labeled and modified in the revised manuscript. (***Pages 9 and 16***)

All responses to the comments are provided above. We are very grateful for all the critical comments and suggestions, and would like to thank all the reviewers again for taking the time to review our revised manuscript. We hope all the revisions have addressed the concerns.

<u>Our point-by-point responses to reviewer' comments are listed as follows:</u>

THE REVIEWER 1#'S REPORT

= = = = = = = = = = = = = = = = = = =

Reviewer #1 (Remarks to the Author):

In the revised manuscript, the authors added more results, including the certified efficiency to address my concerns. Before publication, two more questions should be clarified.

Response: We sincerely appreciate your renewed recognition of our work and recommend it for publication after some revisions. Your valuable suggestions are very important to further improve the quality of our work. In the following, we provide detailed responses to the comments point-by-point, and we hope all the revisions have addressed your concerns.

Q1. As shown in Figure 2i and Table R1, compared to QY, 4Y-BO shows improved molecular ordering and better crystallinity. However, as shown in Figure 3f and Table R2, 4Y-BO has higher EQEel than that of QY. The authors should do more experiments to explain the reason.

Response: Thanks very much for your critical comments. As we know, Urbach energy is interpreted as the width of the tail of the density of state and a measure of energetic disorder. Reducing energetic disorder facilitates to improve the emission and reduces E_{loss} of device. According to the previous reports (*Adv. Funct. Mater.* 32, 2111855 (2022); *Nat. Photon.* 14, 300 (2020); *J. Am. Chem. Soc.* 142, 18741 (2020)), reducing energetic disorder facilitates to enhance the emission of device and thus decrease non-radiative energy loss. Therefore, to better compare the differences between 4Y-BO and QY, we fitted the Urbach energy values based on the highly-sensitive external quantum efficiency (sEQE) spectra, as given in **Fig. R1** below**.** The Urbach energy of PM6:PY-BO and PM6:4Y-BO devices are determined to be 24.3 meV and 23.3 meV, respectively, both of which are significantly lower than that of PM6:QY based device (28.49 meV, *Nat. Commun*. 14, 7996 (2023)). This indicates that the better molecular ordering of 4Y-BO reduces electronic state disorder and result in fewer energy traps and scattering centers, which effectively suppressed the non-radiative recombination for the achievement of higher EQE_{EL}. Furthermore, it has been intensively reported that the introduction of chlorine atoms into non-fullerene acceptors can help to reduce Urbach energy (*Nat. Commun*.10, 2515 (2019); *Aggregate*, 5, e488 (2024); *Adv. Energy Mater.* 9, 1900041(2019); *Dyes Pigm.* 188, 109162

(2021); *Adv. Funct. Mater.* 32, 2111855(2022)). Moreover, the greater intermolecular interactions of 4Y-BO involved acceptor/acceptor and donor/acceptor pairs, as supported by the calculated results (**Fig. R2**), are also helpful to afford more ordered molecular stacking and uniform phase-separations, thereby reducing charge accumulation and improving the luminescence efficiency of injected charges.

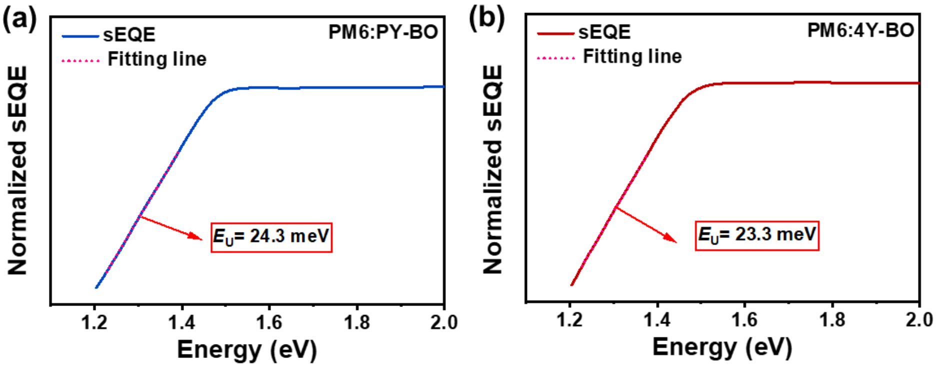

Fig. R1 Normalized sEQE spectra of the PM6:PY-BO and PM6:4Y-BO-based devices.

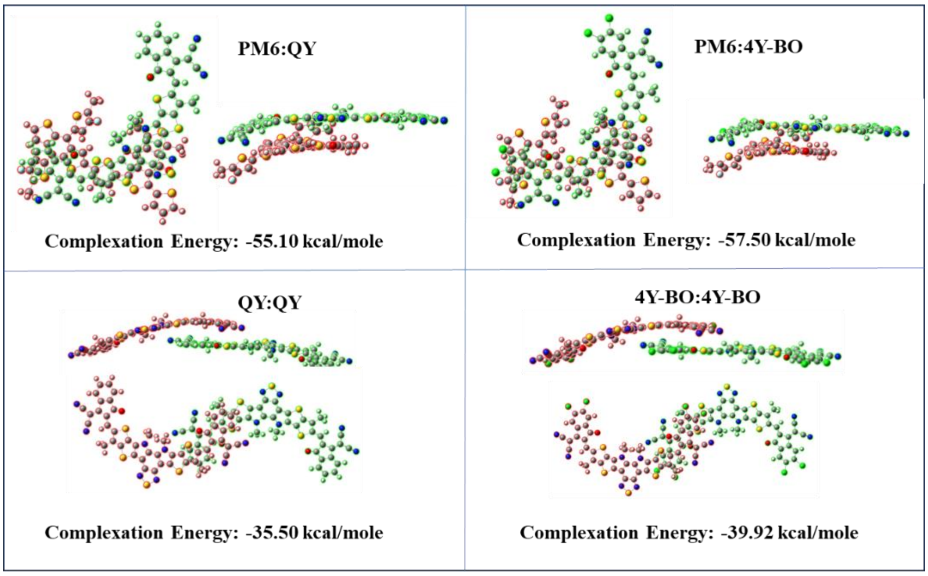

Fig. R2 Optimized intermolecular packing diagrams by density functional theory simulation.

Based on your critical comments and the above analysis, we have incorporated some discussions into the revised manuscript as "Urbach energy (E_U) is further calculated by fitting the sEQE spectra, yielding a smaller E_U of 23.3 meV for PM6:4Y-BO device than that of PM6:PY-BO (24.3 meV). This result uncovers that the improved molecular stacking of 4Y-BO ulteriorly suppresses energetic disorder, which affords fewer energy traps and scattering centers, and effectively reduces non-radiative recombination for higher EQE_{EL}." ***(Page 13, revised manuscript)***

Q2.4Y-BO shows much better thermal stability than QY. How about the photostability?

Response: Thanks for your concern about the photostability of the solar cells. According to your suggestion, we have performed the photostability for PM6:PY-BO and PM6:4Y-BO based devices, and provided the attenuation curves in **Fig. R3** (***incorporated into the revised Supporting Information as Supplementary Fig. 34, Page S20***). The unencapsulated photovoltaic devices were subjected to continuous illumination under solar simulator with light intensity of 100 mW cm^{-2} in the N_2-filled glove box. After illumination for 150 hrs, it can be seen that the PM6:4Y-BO based OSCs maintain 92.6% of its initial PCE, which are higher than that of the benchmark device (83.4%). The improvement in photostability should be partly attributed to the ordered molecular stacking, which enhances charge transport and reduces carrier recombination to suppress material aging and degradation caused by energy accumulation. Meanwhile, the stronger multiple intermolecular interactions, as we have disclosed in Supplementary Fig. 13 and Fig. 29 may help to stabilize the material structure and suppress the generation of detrimental free radicals (*Adv. Mater.* 36, 2412746 (2024); *Mater. Sci. Eng.: R: Rep.* 160, 100828 (2024)).

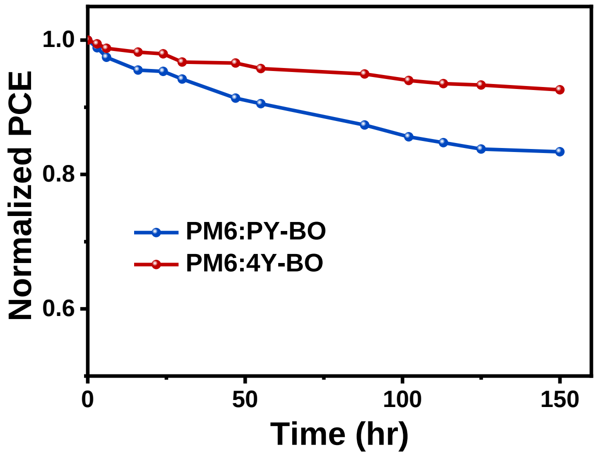

Fig. R3 The photo stability of PM6:PY-BO and PM6:4Y-BO based devices.

Based on your kind suggestion and the above analysis, we have incorporated some discussions into the revised manuscript as "Moreover, the photostability of PM6:PY-BO and PM6:4Y-BO based devices were performed and provided in Supplementary Fig. 34. After continuous illumination for 150 hrs under solar simulator, it can be seen that the PM6:4Y-BO based OSC maintains 92.6% of its initial PCE, significantly higher than that of the PM6:PY-BO device (83.4%). The improvement in photostability should be correlated with the ordered molecular stacking of PM6:4Y-BO blend, which enhances charge transport and reduces carrier recombination to retard material aging and degradation caused by energy accumulation. Meanwhile, stronger intermolecular interactions may help to stabilize

the material structure and suppress the generation of free radicals.[60, 61]" ***(Pages 19-20, revised manuscript)***

and "Besides of superior photovoltaic performance, the PM6:4Y-BO devices also demonstrate notably enhanced thermal stability ($T_{80\%}$ lifetime of 2125 hrs) after continuous heated at 80 °C, and photostability ($T_{92.6\%}$ lifetime of 150 hrs) after continuous illumination." ***(Page 3, revised manuscript)***

In addition, two related references have been cited as **refs. 60, 61** and incorporated into the revised manuscript. ***(Page 28, revised manuscript)***
